# Walking and Daily Affect Among Sedentary Older Adults Measured Using the StepMATE App: Pilot Randomized Controlled Trial

**DOI:** 10.2196/27208

**Published:** 2021-12-01

**Authors:** Alycia N Bisson, Victoria Sorrentino, Margie E Lachman

**Affiliations:** 1 Psychology Department Brandeis University Waltham, MA United States; 2 Psychiatry Department Brigham and Women’s Hospital Boston, MA United States

**Keywords:** physical activity, fitness technology, intervention, behavioral science, aging, mobile phone

## Abstract

**Background:**

Although fitness technology can track and encourage increases in physical activity, few smartphone apps are based on behavior change theories. Apps that do include behavioral components tend to be costly and often do not include strategies to help those who are unsure of how to increase their physical activity.

**Objective:**

The aim of this pilot study is to test the efficacy of a new app, StepMATE, for increasing daily walking in a sample of inactive adults and to examine daily relationships between walking and self-reported mood and energy.

**Methods:**

The participants were middle-aged and older adults aged ≥50 years (mean 61.64, SD 7.67 years). They were randomly assigned to receive either a basic, pedometer-like version of the app or a version with supports to help them determine where, when, and with whom to walk. Of the 96 participants randomized to 1 of 2 conditions, 87 (91%) completed pretest assessments and 81 (84%) successfully downloaded the app. Upon downloading the app, step data from the week prior were automatically recorded. The participants in both groups were asked to set a daily walking goal, which they could change at any point during the intervention. They were asked to use the app as much as possible over the next 4 weeks. Twice per day, pop-up notifications assessed mood and energy levels.

**Results:**

Although one group had access to additional app features, both groups used the app in a similar way, mainly using just the walk-tracking feature. Multilevel models revealed that both groups took significantly more steps during the 4-week study than during the week before downloading the app (γ=0.24; *P*<.001). During the study, the participants in both groups averaged 5248 steps per day compared with an average of 3753 steps per day during the baseline week. Contrary to predictions, there were no differences in step increases between the two conditions. Cognition significantly improved from pre- to posttest (γ=0.17; *P*=.02). Across conditions, on days in which the participants took more steps than average, they reported better mood and higher energy levels on the same day and better mood on the subsequent day. Daily associations among walking, mood, and energy were significant for women but not for men and were stronger for older participants (those aged ≥62 years) than for the younger participants.

**Conclusions:**

Both groups increased their steps to a similar extent, suggesting that setting and monitoring daily walking goals was sufficient for an initial increase and maintenance of steps. Across conditions, walking had benefits for positive mood and energy levels, particularly for women and older participants. Further investigations should identify other motivating factors that could lead to greater and more sustained increases in physical activity.

**Trial Registration:**

ClinicalTrials.gov NCT03124537; https://clinicaltrials.gov/ct2/show/NCT03124537

## Introduction

### Background

The benefits of physical activity for lifelong health, well-being, and cognition are well-documented; yet, most American adults lead an inactive lifestyle [1-3]. According to the Centers for Disease Control and Prevention, only 53% of adults meet the guidelines for aerobic activity, and even fewer older adults meet these guidelines [4]. Fitness technologies such as Fitbit, Apple Watch, or smartphone apps can track and encourage physical activity without the need for additional equipment or a gym membership. Indeed, the *health and fitness* category is one of the most popular categories in the iTunes and Google Play app stores, with almost 230,000 apps available in 2017.

A recent review of 37 Fitbit-based interventions reported that studies were associated with increases in daily steps, moderate to vigorous physical activity, and decreases in body weight [5]. Participants took approximately 950 steps per day more than the controls who were not given a Fitbit. The behavioral components included in the given interventions were related to the success of the interventions [5]. Goal-setting was described as the most promising component; however, a combination of intervention tools may be necessary to encourage changes in physical activity [5]. Another recent review suggested that there is little to moderate evidence that mobile health or eHealth interventions are successful for increasing physical activity in older adults [6].

Although many devices and smartphone apps currently track physical activity, encourage users to meet step goals, and link with other personal data, few stand-alone smartphone apps include additional features that address barriers unique to inactive adults [7]. Furthermore, apps that do include behavior change strategies typically cost more money and do not provide features such as action planning and environmental supports. Focus groups have identified a need for physical activity apps to promote autonomy and self-regulation, while also providing adaptability and flexibility to accommodate individual needs [8].

Implementation intentions involve behavioral strategies such as creating a specific plan to reach a goal [9]. Using walking as an example, implementation intentions could include action planning, which involves creating a plan that includes the time and place that walking would occur [10]. Meta-analyses have shown that action planning is associated with increases in physical activity [11,12]. A recent study tested whether an implementation intentions intervention was more successful in increasing physical activity than just using a Fitbit [13]. The intervention group participants, who were given step goals, personalized walking routes, and a daily schedule to fill out, significantly increased their daily steps over 1 month compared with the control group participants who only wore a Fitbit [13]. Although action planning and environmental supports are rarely incorporated into fitness technology, such strategies may directly address common barriers that prevent adults from engaging in physical activity [7].

### Physical Activity and Affect

Along with the benefits of exercise to physical and cognitive health, many have shown the importance of physical activity for mood and affective states [14,15]. In fact, a recent meta-analysis reported that improved executive functioning and mood, along with decreases in stress, are among the most consistently reported outcomes after exercise [14]. These effects have been echoed in multiple populations and various activity domains, including vigorous activities such as cycling and lower-intensity activities such as yoga or walking. A study showed that patients with multiple sclerosis were more likely to report improved mood after a single 20-minute bout of walking or yoga than after an equivalent period of rest [16]. Others have also found that positive exercise experiences are linked to increases in motivational self-efficacy and exercise intentions, which then predict future exercise behavior [17].

New technologies have made it possible to examine the relationships among physical activity, mood, and affect in real time using accelerometry along with methods such as experience sampling or ecological momentary assessments (EMAs). A study used a newly developed smartphone app and EMAs to test whether self-reported happiness and physical activity are linked [18]. The results from >10,000 app users showed that more active individuals reported being happier than those who were inactive. Daily relationships also emerged; people were happier on more physically active days than on less active ones [18].

A review paper by Liao et al [19] summarized 14 studies that used EMAs to examine short-term relationships between physical activity and affect. The authors found evidence for reciprocal relationships; current positive affect predicted increased physical activity within the next few hours, and physical activity engagement predicted greater positive affect within the next few hours [19]. Thus, it seems that positive affect predicts subsequent physical activity, which also predicts future positive affect.

It is possible that men and women experience differential effects of exercise on mood; however, very little work has examined sex differences. A study found that in young adults, women were more likely to report improvements in mood after exercise than men [20]. The same study found that women were more likely than men to report reduced fatigue after a 30-minute bout of exercise [20]. It is possible that women are more sensitive to mood changes after exercise.

In sum, physical activity and affect have been linked at both the within-person and between-person levels. The effects are similar across various domains of physical activity and in healthy and nonhealthy adult populations. Affective changes can be seen from acute (20-minute) bouts of activity to regular activity over the course of months. Although prior studies have examined affective improvements in the context of structured exercise, no studies to our knowledge have tested whether the number of steps one takes per day is predictive of contemporaneous changes in affect. Furthermore, few studies have closely examined whether the effects differ between men and women.

### Physical Activity, Sleep, and Energy

Another consistent finding in the literature is the relationship between physical activity and sleep. When examining average levels of physical activity, people who are more active tend to sleep better than those who are less active [21]. Most of this work has focused on high-impact physical activity or on populations with sleep disorders or other health problems. Daily studies suggest that on the days in which people are more active, they sleep better and longer than on less active days [22-24]. Recent work found that women who average more steps per day over the course of a month reported better sleep quality than inactive women, not men [22].

A study examined the relationship among physical activity, affect, and insomnia symptoms in a sample of inactive adults with insomnia [25]. Those who were asked to engage in consistent walking reported significant decreases in insomnia symptoms, along with improved affect, over the 6-month intervention [25]. Taken together, the results suggest that even low-impact physical activity such as walking or yoga can improve sleep in adults. Those who sleep well will likely report higher energy levels during the day; however, self-reports of energy are also affected by other things that happen on any given day. Although the link between physical activity and sleep has been studied, less is known about how daily physical activity is related to self-reported daily energy levels.

This study aims to test whether an iPhone (Apple Inc) app—StepMATE—with behavioral supports was associated with increases in daily walking among a sample of inactive but otherwise healthy adults. We also aim to examine whether a version of the app with additional action planning strategies is more successful than a version with only step-tracking and daily step goals. Finally, we aim to assess within-person fluctuations in steps, mood, and energy and whether there are differences in these relationships based on demographic characteristics, including age and sex.

## Methods

### Study Details

**Figure 1:**
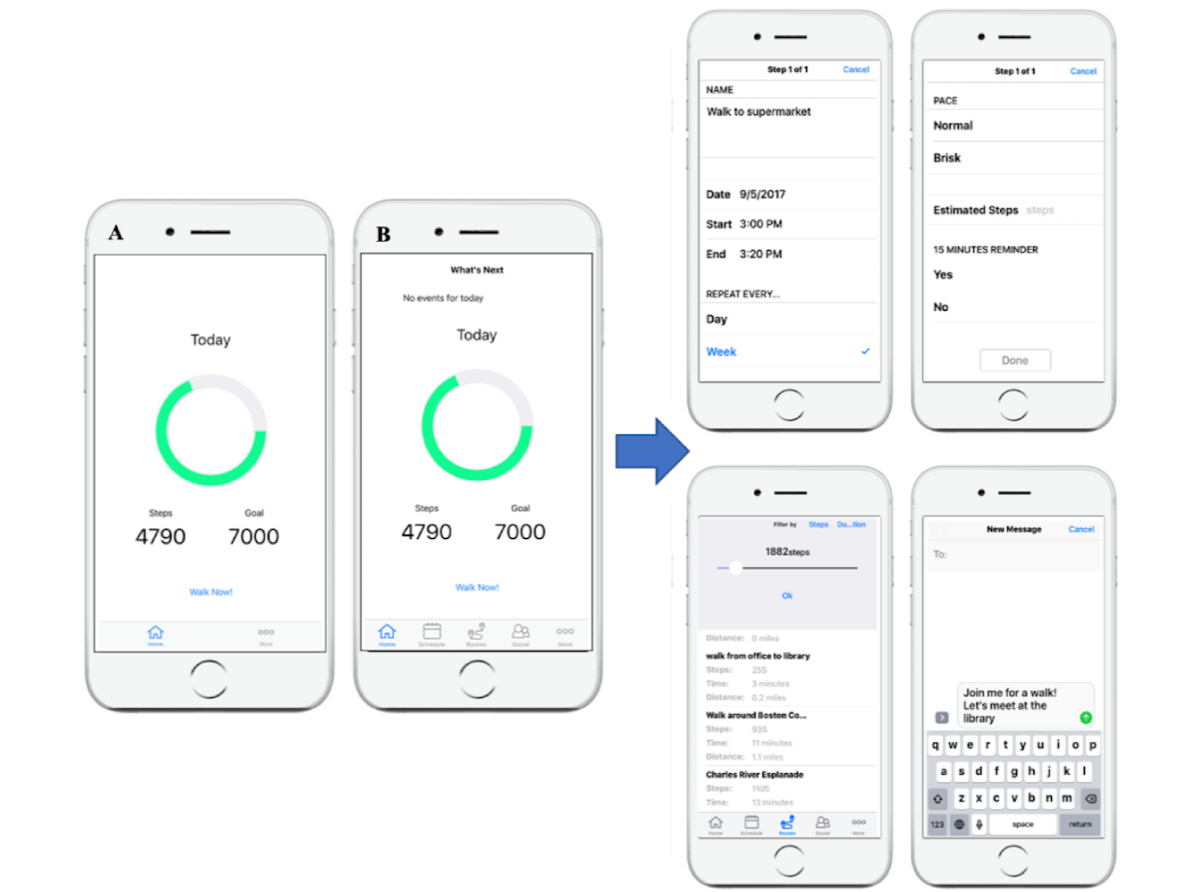
Screenshots of the StepMATE app. (A) The home screen for the control group. (B) The home screen for the treatment group members, who had access to the additional features shown in the screenshots on the right.

On the basis of our previous study on midlife adults that assessed barriers to being physically active [13], along with the findings from pilot interviews of 9 older adults, we found that perceived lack of time was a common barrier preventing people from getting enough exercise. Other barriers reported in these studies were not knowing where to exercise and not wanting to exercise alone. The StepMATE app (Figure 1) was developed by Beneufit using Apple ResearchKit, with feedback from university researchers. StepMATE is a fully automated app that includes behavioral supports to help people plan where and when to walk and social supports to help find others who might want to walk with them.

The participants in the StepMATE pilot randomized controlled trial were randomly assigned to receive 1 of 2 versions of the app. The control group was only given step-tracking and goal-setting functions, similar to those of a Fitbit, and the treatment group was given a version of the app with additional social and environmental supports. First, we tested whether the app was associated with increases in average daily steps over the 4 weeks and whether there were differences between the 2 conditions. It was hypothesized that the additional supports would result in greater increases in walking for the treatment group compared with the control group. Next, we examined whether there were changes over time or between-group differences in other outcomes, including sleep, exercise control, exercise self-efficacy, social engagement, and memory. We hypothesized that the participants would report improvements in these outcomes from pre- to posttest, with greater improvements in the treatment group. Finally, within-person relationships between daily steps and self-reported mood and energy were modeled. We hypothesized that on the days when the participants took more steps than average, they would report higher energy levels and better mood than on less active days. Drawing from prior findings on sex differences in daily relationships between exercise and other outcomes, the interactions between daily steps and sex on mood and energy were examined. It was hypothesized that daily steps would be more closely related to mood and energy in women than in men. Exploratory analyses examined whether there were interactions between daily steps and age in predicting mood and energy.

### Participants

**Figure 2:**
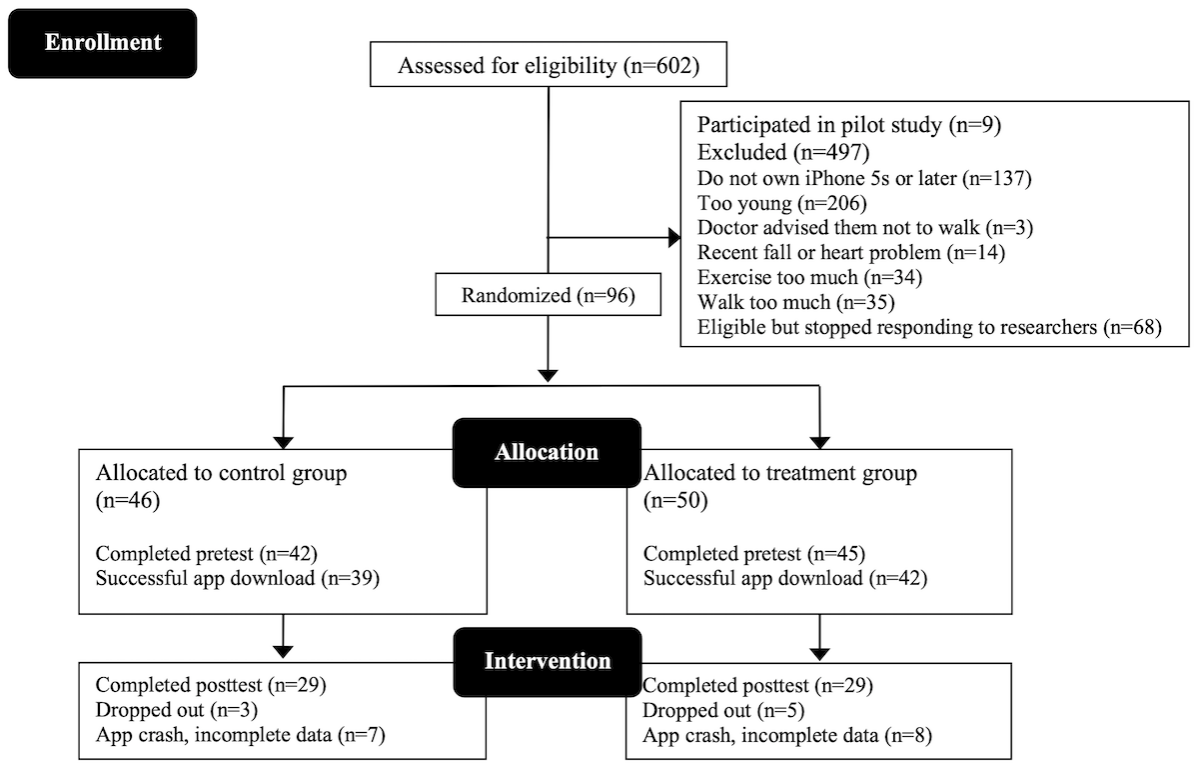
CONSORT (Consolidated Standards of Reporting Trials) diagram.

The participants were recruited on the web on a rolling basis between January 2018 and March 2019 using Facebook, Craigslist, and FindParticipants. Participants were also recruited locally in eastern and central Massachusetts through flyers at senior centers, libraries, cafes, and community events. As the study did not require an in-person meeting, participants were recruited from locations across the continental United States. The CONSORT table is presented in Figure 2, detailing recruitment, enrollment, and exclusion criteria for this study.

The participants were required to own an iPhone with a built-in accelerometer to measure steps (iPhone 5s or newer). Only those who reported exercising less than the Centers for Disease Control and Prevention guidelines of 150 minutes of moderate to vigorous exercise per week were eligible. The participants also needed to report walking for exercise no more than 30 minutes per day [26]. Participants were ineligible if a physician advised them not to walk because of health conditions or if they had had a cardiac event or fall within the last 6 months. Screening for cognitive impairment was conducted over the phone using a shortened version of the Short Portable Mental Status Questionnaire [27]. Participants were ineligible if they made ≥3 errors on this questionnaire.

All procedures were approved by the university institutional review board. An a priori power analysis for the primary outcome variable, number of steps, was conducted using G*Power (version 3.1; Heinrich Heine University) [28], which indicated that 31 participants per condition were required with an estimated effect size of *d*=0.10, with 95% power at *P*=.05.

After an iPhone software update, the StepMATE app crashed and did not work properly for approximately 2 weeks. Of the 81 participants who downloaded the app, 18 (22%) were affected, and daily step data were lost for these participants. Analyses were conducted on an intent-to-treat basis. For the questionnaire data, we analyzed all participants who completed the pretest measures (87/96, 91%), and for the daily step analyses, all participants with sufficient step data were included in the analyses (80/81, 99%). Sample sizes are included in all results tables for clarity.

### Pre- and Posttest Measures

#### Social Engagement

Social engagement was measured using the Lubben Social Network Scale [29]. This scale comprises 12 items (6 related to family and 6 to friends) that ask about the size of one’s social network (eg, How many friends or family members do you feel at ease with that you can talk about private matters?) and the closeness of the relationships (eg, How often do you see or hear from the friend or family member with whom you have the most contact?). A composite score was calculated by summing the responses of the 12 items, with a final score ranging from 0 to 60, where a higher score indicates more social engagement. The Cronbach α value for the internal consistency in this sample was .862.

#### Exercise Control

Control over exercise was measured using the 6-item Exercise Control Beliefs Scale [30]. The items assess one’s beliefs about one’s control over exercise (eg, I am confident in my ability to do an exercise routine), with answer choices ranging from strongly disagree (score=1) to strongly agree (score=5). The 6 items were averaged to create a mean exercise control score, with a higher score indicating greater control over exercise. The Cronbach α value for the internal consistency in this sample was .604.

#### Exercise Self-efficacy

A modified version of the Bandura Exercise Self-Efficacy Scale [31] was used in this study. This 9-item scale assesses how sure an individual is that they would exercise under different conditions or constraints (eg, How sure are you that you will exercise when you are feeling tired or under pressure to get things done?), with answer choices ranging from not sure at all (score=1) to very sure (score=4). The 9 items were averaged to create a composite score, where a higher score indicates greater exercise self-efficacy. The Cronbach α value for the reliability of this scale was .935.

#### Cognitive Performance

Cognition was assessed using a shortened version of the Brief Test of Adult Cognition by Telephone (BTACT) [32]. This version of the BTACT assesses 5 cognitive dimensions, including 2 measures of episodic verbal memory (immediate and delayed free recall of 15 words), working memory (backward digit span), verbal fluency (the number of words produced from a given category within 60 seconds), and processing speed (counting backward from 100 in 30 seconds). The primary outcome measure was a composite of all cognitive tests. The scores on both occasions (pre- and posttest) were standardized based on the scores at the pretest to create cognitive composites. The BTACT is a reliable assessment of cognitive functioning; its psychometric properties have been reported in another manuscript [32].

#### Sleep

Sleep duration and quality were measured using the Pittsburgh Sleep Quality Index (PSQI) [33]. The PSQI global score could range from 0 to 21, with a higher score indicating *worse* sleep. The Cronbach α value for the reliability of the 7 subscales of the PSQI was .77. In this study, we examined the PSQI global score, along with raw scores for duration (average number of hours slept during the past month) and latency (average number of minutes taken to fall asleep during the past month).

### Daily Measures

#### App Engagement

To assess the use of various app features, for each participant, the total number of SMS text messages sent to contacts, number of routes saved, number of scheduled events, and number of times the *Walk Now* feature was used were computed.

#### Physical Activity

Every day, over the course of a month, physical activity was assessed using the total number of steps taken each day. Daily steps were quantified using the iPhone’s built-in accelerometer and recorded through the StepMATE app. When the participants downloaded StepMATE at the beginning of the study, the app automatically and retroactively recorded daily steps from the week before the start of the study. During the 4-week intervention, the participants were asked to carry their phone with them during the day; however, they were not specifically instructed to do so during the baseline week before the intervention began.

Although data indicate that older adults typically average between 2000 and 9000 steps per day [34], there are likely times when the participants walked without carrying their iPhone. For the days when the iPhone recorded fewer than 500 steps, that day of steps was coded as missing. Weekly step averages were calculated for weeks with 4 or more days with 500 or more daily steps. Of the 81 participants included in this intent-to-treat analysis, 11 (14%) had missing or incomplete baseline data.

#### Daily Affect: Mood and Energy

Twice, at random times each day, mood and energy levels were assessed. A pop-up notification asked the participants to rate their current mood (unhappy, neutral, or happy) and energy (low, neutral, or high) on a slider scale. The scores were converted by using the StepMATE app to a 0-10 scale, with 0 indicating low mood or energy and 10 indicating high mood or energy. The 2 daily ratings were averaged to provide a daily average of the participants’ mood and energy.

#### Covariates

Age, sex, education, and health were covariates in the current set of analyses because they were expected to be related to the outcomes. In models where time×condition interactions were not estimated, condition was included as a covariate. Age was continuous, sex was coded as 1=male and 2=female, and education was number of years in school. Health was measured using the general health subscale from the Medical Outcomes Study 36-item Short-Form Health Survey [35]. Condition was coded as 0=control group and 1=treatment group.

### Design and Procedures

#### App

The StepMATE app was designed to help participants determine when, where, and with whom they would add physical activity to their day. Daily step goals were set by the participants in both the intervention and control groups, and all participants had the ability to change their step goal at any point throughout the study. For the *when* component, those in the intervention condition had a scheduling feature in the app. The participants could schedule a block of time to go for a walk, and they had the option to create a reminder, set recurring events, and estimate the number of steps they would get in that walk. Once an event was created, it appeared in both the StepMATE app and the iPhone’s built-in calendar.

For the *where* component, those in the intervention group were able to create, name, and save walking routes in the app. When the participants in the intervention group hit *Walk Now*, StepMATE began keeping track of their geographical location, distance, number of steps, and total time of the walk. This information was then saved after the walk was finished so that the time it took to walk a route could be compared if the same route was walked again. When a user created multiple routes, they could be filtered by number of steps or duration so that the user could easily find a walk that fit the amount of time they had or the number of steps they needed to achieve their daily walking goal. Those in the control group also had a *Walk Now* button; however, when the control participants hit *Walk Now*, the app would simply track the number of steps taken in that walk. These participants were not able to name or save their walks, nor could they view their walks on a map.

For the *with whom* (social feature) component, those in the intervention condition had the option to text one of their iPhone contacts through the app and invite them for a walk. Those in the control condition did not have access to this feature. Multimedia Appendix 1 includes screenshots of the app, video tutorials of the app features, and differences between the 2 versions, as well as descriptions of other app functions.

#### Procedures

A research assistant used Microsoft Excel for the block randomization procedures. Blocks of 10 consecutive ID numbers were randomly assigned to 1 of the 2 conditions. The app developer received the lists of ID numbers and associated treatment condition so that when an ID number was provided during the app download, the correct version of the app would install on the participant’s phone. Upon meeting the inclusion criteria and consenting to participate in the study, each participant was assigned an ID number that was paired to the condition generated from the block randomization. Next, the participants were administered a shortened version of the BTACT, and they completed the prestudy questionnaires on the web through Qualtrics, including self-assessed social engagement, exercise control, exercise self-efficacy, and sleep. The participants filled out their ID number at the beginning of the Qualtrics survey so that their self-report data could easily be linked to their step data.

Subsequently, the researchers scheduled a phone call to help the participants to download the app, set up their account, including daily walking goals, and thoroughly explain the app features. Those in the control condition downloaded a version with only the daily step goals and the ability to track time, distance, and steps within a walk. Those in the treatment condition had access to these and additional features, including schedules, maps, and social features. The participants were blinded to which condition they were assigned to receive. Although the researchers were aware of the condition assignment for the purposes of helping with app downloads and troubleshooting issues, all measures—except for the cognitive assessments—were carried out on the web without researcher involvement. Randomization was checked by comparing the covariates (age, sex, education, and general health) between the conditions using independent samples *t* tests (2-tailed). No significant differences were found between the conditions for any of these variables.

The participants in both groups were asked to use the app for 1 month and do their best to answer the daily mood and energy questions. All participants were sent a pouch to wear around their waists and were encouraged to use it to carry their phone with them as much as possible until they went to bed each night. After the first and third weeks, the participants received an email letting them know how many weeks had elapsed in the study and how many weeks remained. After the second week, the researchers called the participants to ask some open-ended feedback questions and ensure that there were no problems with the app. If any problems arose during the intervention, the participants had access to a *Help* section within the app that included a phone number and email address to contact the researchers. This information was also included in the paper intervention materials that were mailed to them and attached to all email communications.

At the completion of the 1-month study, the participants in both groups were again administered the shortened version of the BTACT and asked some open-ended feedback questions, after which they were asked to complete the poststudy questionnaires on the web through Qualtrics. After completing the questionnaires, the participants were sent a US $25 Amazon gift card through email. After the posttest, the participants in the control condition were given the opportunity to download the full version of the app, and all participants were encouraged to retain and use the app for their personal use.

### Data Analysis

Data analyses were conducted using RStudio (version 1.2.1335; RStudio, PBC) [36]. First, the difference in app engagement between the conditions was examined. We compared use of the *Walk Now* feature between the conditions using independent samples *t* tests. The use of the schedule and social functions was tallied for the intervention condition.

We tested the remainder of our hypotheses with multilevel mixed effects modeling with the lme4 package [37], controlling for age, sex, education, and health. Using the following model, we tested whether weekly average steps increased from the baseline week to the 4 intervention weeks. Sensitivity analyses tested whether this effect differed if the baseline week was excluded. Interactions were specified to determine whether the change in weekly step averages differed between the conditions.

Level 1: Step Average*_ij_* = β*_0j_* + β*_1j_* (Week)* *+ r*_ij_*

Level 2: β*_0j_* = γ*_00_* + γ*_01_* (Age*_j_*) + γ*_02_* (Sex*_j_*) + γ*_03_* (Condition*_j_*) + γ*_04_* (Education*_j_*) + γ*_05_* (Health*_j_*) + u*_0_ j *

β*_1j_* = γ*_10_* + γ*_11_* (Condition*_j_*)

Next, we used the following model to examine changes in the other outcome measures between pre- and posttest, including social engagement, exercise control and self-efficacy, memory, and sleep. Interactions were examined to determine whether the change in outcomes differed between the conditions.

Level 1: Outcome Measure*_ij_* = β*_0j_* + β*_1j_* (Time)* *+ r*_ij_*

Level 2: β*_0j_* = γ*_00_* + γ*_01_* (Age*_j_*) + γ*_02_* (Sex*_j_*) + γ*_03_* (Condition*_j_*) + γ*_04_* (Education*_j_*) + γ*_05_* (Health*_j_*) + u*_0j_*

β*_1j_* = γ*_10_* + γ*_11_* (Condition*_j_*)

Finally, within-person relationships among daily steps, mood, and energy levels were tested. The following models tested whether daily steps were associated with same-day mood and energy. Lagged analyses were used to determine whether steps predicted next-day mood and energy, controlling for previous day mood and energy. To parse out between-person and within-person effects, the models included both weekly average steps and daily deviation from average steps as predictors. Exploratory analyses examined whether sex or age moderated these effects. In instances when significant interactions with sex were found, separate models were run with men and women to probe the interaction. When significant age interactions were found, separate models were run by using a median split of age in our sample (62 years).

Level 1: Daily Mood or Energy*_ij_* = β*_0j_* + β*_1j_* (Daily Steps)* *+ r*_ij_*

Level 2: β*_0j_* = γ*_00_* + γ*_01_* (Age*_j_*) + γ*_02_* (Sex*_j_*) + γ*_03_* (Condition*_j_*) + γ*_04_* (Education*_j_*) + γ*_05_* (Health*_j_*) + γ*_06_* (Average Steps*_j_*) + u*_0j_*

β_1j =_
*γ_10_ + u_1j_*

## Results

### Participants

These analyses included adults aged ≥50 years (mean 61.87, SD 7.82 years). Of the 87 participants, 61 (70%) were women, 75 (86%) were White (86%), 2 (2%) reported being Asian, 9 (10%) reported being Black or African American, and 1 (1%) did not wish to report race. The participants were well-educated, with an average of 16.45 (SD 2.56) years of education. Health, on average, was 69.25 (SD 17.40; as reported on the 36-item Short-Form Health Survey general health subscale, with a 0-100 range). Of the 87 participants, 27 (31%) reported working full time, 17 (20%) reported working part time, 34 (39%) were retired, 6 (7%) reported that they were self-employed, and 3 (3%) reported being a homemaker.

### Correlations Among Primary Outcome Variables

Zero-order correlations were computed among all outcome variables and covariates at pre- and posttest (Multimedia Appendix 1, Table S1). The average number of steps taken throughout the intervention was positively correlated with exercise self-efficacy at posttest (*r*=0.33; *P*=.01). Average steps were also significantly correlated with sleep duration (*r*=–0.29; *P*=.03) and sleep latency (*r*=0.27; *P*=.04)*.* At pretest, average mood was significantly correlated with age (*r*=0.26; *P*=.02), health (*r*=0.29; *P*=.01), social engagement (*r*=0.30; *P*=.007), exercise control (*r*=0.32; *P*=.004), exercise self-efficacy (*r*=0.24; *P*=.03), PSQI global score (*r*=–0.41; *P*<.001), sleep latency (*r*=–0.36; *P*=.001), and average energy (*r*=0.67; *P*<.001)*.* At posttest, average mood was significantly correlated with social engagement (*r*=0.34; *P*=.008), PSQI global score (*r*=–0.27; *P*=.04), and average energy (*r*=0.70; *P*<.001)*.* At pretest, average energy was significantly correlated with health (*r*=0.40; *P*<.001), social engagement (*r*=0.30; *P*=.008), exercise control (*r*=0.36; *P*=.001), exercise self-efficacy (*r*=0.23; *P*=.04), PSQI global score (*r*=–0.42; *P*<.001), sleep latency (*r*=–0.26; *P*=.02), and average mood (*r*=0.67; *P*<.001)*.* At posttest, average energy was significantly correlated with health (*r*=0.40; *P*=.002), social engagement (*r*=0.27; *P*=.04), exercise control (*r*=0.39; *P*=.003), exercise self-efficacy (*r*=0.42; *P*=.01), PSQI global score (*r*=–0.33; *P*=.01), and average mood (*r*=0.70; *P*<.001)*.*

### App Use

The participants in both groups were able to use the *Walk Now* feature; however, only the treatment group participants were able to see their walking routes on a map and name and save them. In the control condition, of the 39 participants, 28 (72%) used the *Walk Now* feature, with an average of 2363 (SD 1616) steps per walk. In the treatment condition, of the 42 participants, 24 (57%) used the *Walk Now* feature, with an average of 1939 (SD 791) steps per walk. An independent samples *t* test showed that the group difference in average steps per walk was not significant (t_50_=1.17; *P*=.25). On the basis of the use of the *Walk Now* feature, those in the control group took an average of 9 (SD 11) walks, whereas those in the intervention group took an average of 11 (SD 24) walks over the course of the 1-month study. An independent samples *t* test showed that there was no significant group difference in the average number of walks taken (t_79_=–0.56; *P*=.58).

Of the participants in the treatment group, only 5 used the schedule feature at least once; 1 participant used the schedule feature 3 times, whereas the other 4 used it once. Only 4 of the treatment group participants used the social feature to text friends through the app; each of these participants used it once during the 1-month intervention.

In terms of correlations between the covariates and app use, those who were older used the *Walk Now* feature more often (*r*=0.26; *P*=.02). Age was not significantly correlated with use of the schedule or social features. Neither sex nor education was significantly correlated with use of the *Walk Now* feature, schedule, or social features.

### Weekly Steps

Table 1 shows the average daily steps by condition and week.

**Figure 3:**
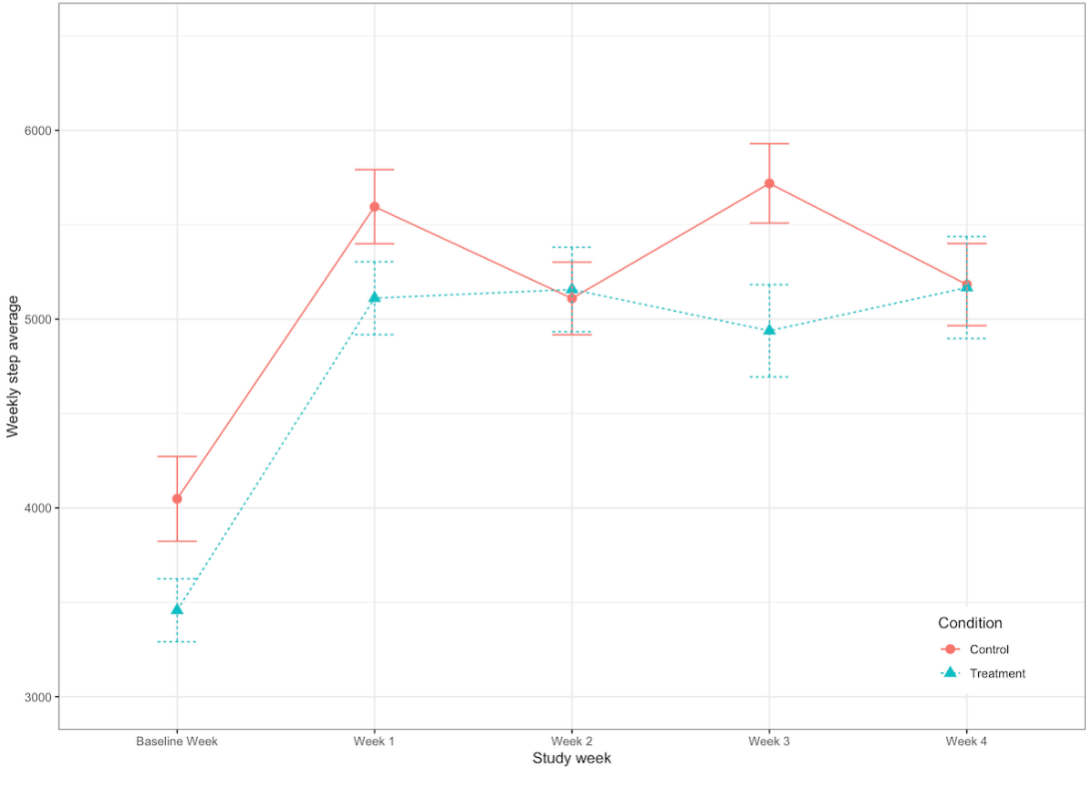
Weekly step averages by condition. The error bars refer to SE of the mean. There was a significant positive main effect of week; however, time×condition interactions were not significant.

After controlling for age, sex, education, health, and condition, we found that there was a significant main effect of time (including the baseline week) in predicting weekly average steps (γ=0.24; *P*<.001; Table 2; Figure 3). Average daily steps were significantly higher during the 4-week study than during the baseline week. Analyses were rerun with the baseline week excluded to determine whether average daily steps increased over the course of the 4-week study. There was no significant change in average steps over the 4 intervention weeks (γ= –0.12; *P*=.1; Table 2). Weekly step averages did not differ between the control and intervention groups, nor were there any significant time×condition interactions.

**Table 1:** Descriptive statistics of baseline measures by condition (N=87)^a^.

Characteristics	Control condition		Treatment condition		Combined	
	Values, n (%)	Values, mean (SD)	Values, n (%)	Values, mean (SD)	Values, N	Values, mean (SD)
Age (years)	42 (48)	61.64 (7.67)	45 (52)	61.51 (8.05)	87	61.57 (7.82)
Sex	42 (48)	71^b^	45 (52)	69^b^	87	70^b^
Education (years)	41 (48)	16.71 (2.21)	45 (52)	16.22 (2.85)	86	16.45 (2.56)
Health	42 (48)	66.31 (16.04)	45 (52)	72.00 (18.32)	87	69.25 (17.40)
Baseline steps	34 (49)	4043.68 (2872.70)	36 (51)	3411.67 (1631.32)	70	3718.64 (2323.34)
Week 1 steps	39 (49)	5530.37 (2286.37)	41 (51)	5046.48 (2426.88)	80	5282.37 (2357.09)
Week 2 steps	38 (48)	5036.25 (2231.26)	41 (52)	4958.95 (2913.55)	79	4996.13 (2606.02)
Week 3 steps	36 (51)	5667.82 (2408.19)	34 (49)	4897.45 (2813.94)	70	5293.64 (2622.80)
Week 4 steps	33 (41)	5175.53 (2501.43)	42 (52)	4570.65 (2425.45)	81	4800.66 (2328.22)
Average steps^c^	39 (48)	5082.83 (2242.57)	42 (52)	4570.65 (2425.45)	81	4813.94 (2339.82)
Average mood^c^	39 (48)	6.85 (1.99)	42 (52)	6.56 (2.10)	81	6.71 (2.06)
Average energy^c^	39 (48)	5.73 (2.07)	42 (52)	5.80 (2.24)	81	5.76 (2.16)

^a^There were no significant differences in age, sex, education, baseline steps, or health between the conditions at baseline.

^b^Percentage values.

^c^Averages across the 4-week intervention.

**Table 2:** Unstandardized coefficients from multilevel week×condition interaction on daily steps (N=80)^a^.

Outcome	Model 1^b^: steps with baseline				Model 2^c^: steps without baseline		
	β	SE	*P* value	β		SE	*P* value
Intercept	5.13	2.61	.05	5.47		2.78	.05
Week	.24	0.05	<.001	–.12		0.07	.1
Age (years)	–.02	0.03	.64	–.01		0.04	.77
Sex	–1.13	0.52	.03	–1.16		0.55	.04
Condition	–.51	0.55	.35	–.57		0.63	.37
Education	–.03	0.10	.72	–.03		0.10	.75
Health	.03	0.01	.07	.03		0.01	.04
Days of app use	.03	0.05	.52	.04		0.05	.4
Week×condition interaction	.02	0.07	.76	.02		0.10	.82

^a^Daily steps were rescaled by dividing the number of steps by 1000; therefore, the β value reflects change per 1000-step increase.

^b^Model 1: level 1 variance=4.44 (SD 2.11); level 2 variance=6.05 (SD 2.46). Akaike information criterion=11319.5; Bayesian information criterion=11383.1; log likelihood=–5648.8.

^c^Model 2: level 1 variance=4.98 (SD 2.23); level 2 variance=5.67 (SD 2.38). Akaike information criterion=9038.6; Bayesian information criterion=9099.8; log likelihood=–4508.3.

### Group Differences in Changes for Other Outcome Variables

Table 3 details the pre- and post-intervention outcomes by condition. After controlling for age, sex, education, and health, we found that there were no changes in the PSQI global score, sleep duration, or sleep latency between the pre- and posttests. There was a significant main effect of time in predicting cognitive performance (γ=0.17; *P*=.02; Multimedia Appendix 1, Table S2). Cognitive performance increased between the pre- and posttests. There were no significant main effects for time, condition, or significant time×condition interactions for other outcomes, including sleep, social engagement, exercise control, or exercise self-efficacy (Multimedia Appendix 1, Tables S2 and S3).

**Table 3:** Descriptive statistics for pre- and posttest variables by condition^a^.

	Pretest				Posttest		
	Control (n=42), mean (SD)	Treatment (n=45), mean (SD)	Combined (N=87), mean (SD)	Control (n=29), mean (SD)		Treatment (n=29), mean (SD)	Combined (N=58), mean (SD)
PSQI^b^ global score	5.71 (3.78)	5.27 (4.14)	5.48 (3.96)	5.45 (3.69)		5.59 (4.44)	5.52 (4.05)
Sleep duration	6.69 (1.13)	6.87 (1.13)	6.78 (1.13)	6.59 (1.03)		7.03 (1.21)	6.81 (1.13)
Sleep latency	18.08 (13.45)	24.47 (26.80)	21.39 (21.54)	19.35 (12.09)		26.37 (27.72)	22.86 (21.49)
Cognitive performance	0.05 (0.61)	0.02 (0.58)	0.03 (0.59)	0.18 (0.29)		0.18 (0.35)	0.18 (0.32)
Social engagement	36.14 (7.65)	33.07 (10.86)	34.55 (9.52)	35.10 (8.62)		30.55 (12.15)	32.83 (10.69)
Exercise control	4.32 (0.51)	4.30 (0.57)	4.31 (0.54)	4.22 (0.63)		3.98 (0.68)	4.10 (0.66)
Exercise self-efficacy	2.57 (0.69)	2.82 (0.81)	2.70 (0.76)	2.40 (0.85)		2.54 (0.97)	2.47 (0.90)

^a^There were no significant differences between the conditions for any of these variables: Pittsburgh Sleep Quality Index, cognitive performance (Brief Test of Adult Cognition by Telephone cognitive composite), and social engagement (Lubben Social Network Scale).

^b^PSQI: Pittsburgh Sleep Quality Index.

### Daily Affect

#### Overview

The relationships among steps, mood, and energy were examined next. As there were no condition differences in weekly steps and no significant time×condition interactions for any of the pre- and post-intervention outcomes, time×condition interactions were not estimated for subsequent analyses. Condition was, however, included as a covariate.

#### Mood

**Figure 4:**
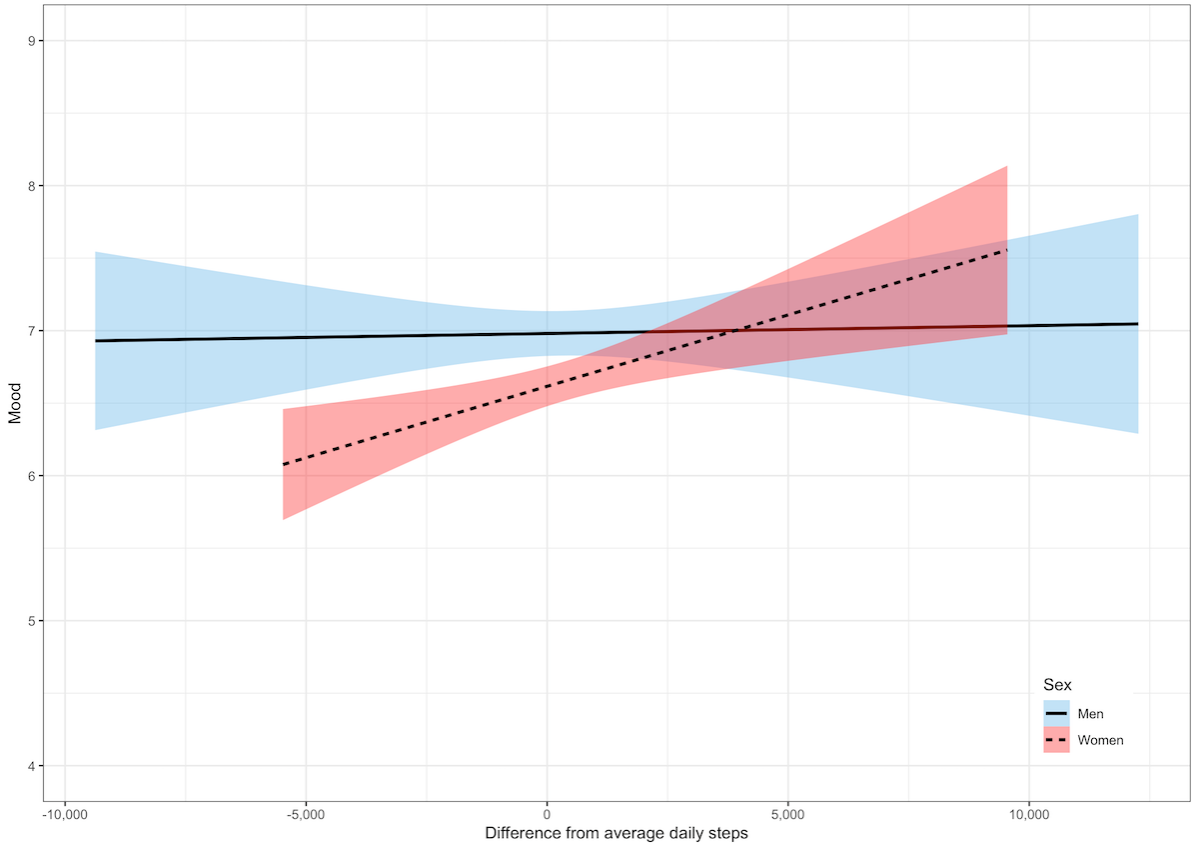
Within-person relationships between daily steps and mood by sex. A score of 0 denotes within-person average daily steps. Shaded areas represent 95% CIs.

**Figure 5:**
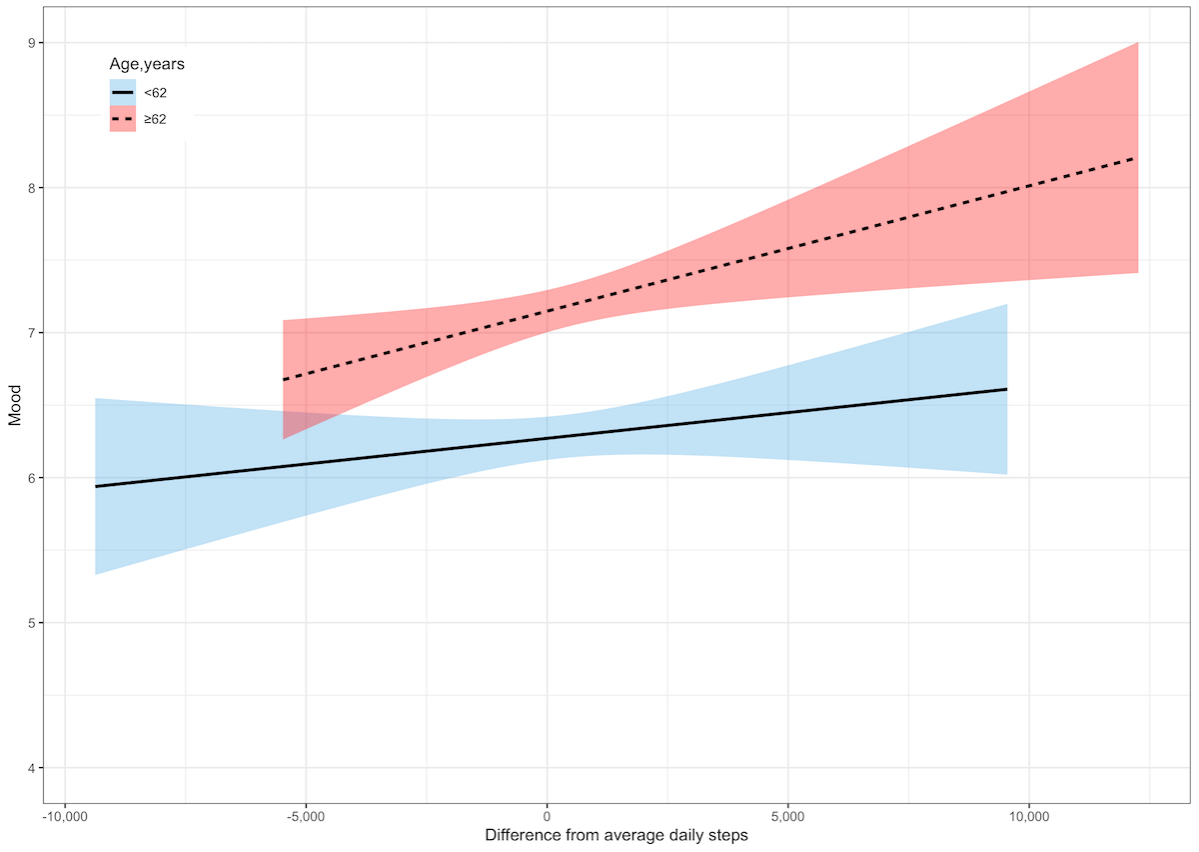
Within-person relationships between daily steps and mood by age group. A score of 0 denotes within-person average daily steps. Shaded areas represent 95% CIs.

After accounting for covariates, on the days when the participants took more steps than they did on average, they reported better mood (γ=0.06; *P*<.001; Table 4). There was a significant interaction between daily steps and sex (γ=0.08; *P*=.01; Table 4). The relationship between daily steps and mood was significant for women (γ=0.09; *P*<.001) but not for men (γ=0.009; *P*=.63; Figure 4). There was also a significant interaction between daily steps and age (γ=0.005; *P*=.02; Table 4). The relationship between daily steps and energy was stronger for adults aged ≥62 years (γ=0.08; *P*<.001) than it was for those aged ≤62 years (γ=0.04; *P*=.05; Figure 5).

**Table 4:** Unstandardized coefficients from multilevel effects of daily steps on same-day mood (N=79)^a^.

Outcome	Model 1^b^: mood				Model 2^c^: mood				Model 3^d^: mood		
	β	SE	*P* value	β		SE	*P* value	β		SE	*P* value
Intercept	3.18	1.90	.01	3.22		1.90	.09	3.22		1.90	.09
Daily steps	.06	0.02	<.001	–.07		0.05	.19	–.23		0.12	.06
Age (years)	.04	0.02	.13	.04		0.02	.13	.04		0.02	.14
Sex	–.47	0.40	.25	–.50		0.40	.22	–.47		0.40	.25
Condition	–.50	0.37	.18	–.49		0.37	.19	–.49		0.37	.18
Education	.05	0.07	.49	.05		0.07	.49	.05		0.07	.49
Health	.03	0.01	.02	.03		0.01	.02	.03		0.01	.02
Average steps	–.09	0.08	.29	–.09		0.08	.28	–.09		0.08	.29
Daily steps×sex	—^e^	—	—	.08		0.03	.01	—^e^		—	—
Daily steps×age	—^e^	—	—	—^e^		—	—	.005		0.002	.02

^a^Daily steps were rescaled by dividing the number of steps by 1000; therefore, the β value reflects change in mood per 1000-step increase.

^b^Model 1: level 1 variance=2.38 (SD 1.54); level 2 variance=1.60 (SD 1.27). Akaike information criterion=4981.2; Bayesian information criterion=5033.8; log likelihood=–2480.6.

^c^Model 2: level 1 variance=2.38 (SD 1.54); level 2 variance=1.60 (SD 1.26). Akaike information criterion=4976.8; Bayesian information criterion=5034.7; log likelihood=–2477.4.

^d^Model 3: level 1 variance=2.39 (SD 1.55); level 2 variance=1.60 (SD 1.26). Akaike information criterion=4977.7; Bayesian information criterion=5035.5; log likelihood=–2477.8.

^e^Outcome was not included in the model.

#### Energy

**Figure 6:**
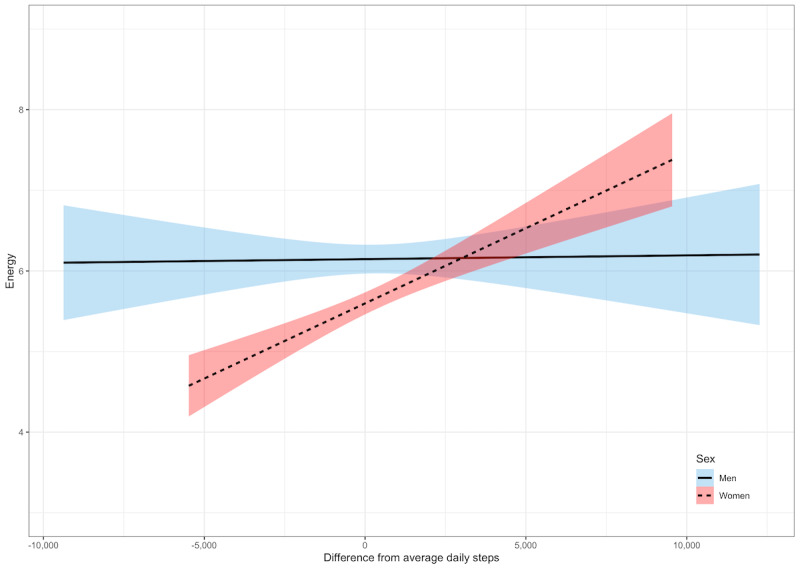
Within-person relationships between daily steps and energy by sex. A score of 0 denotes within-person average daily steps. Shaded areas represent 95% CIs.

**Figure 7:**
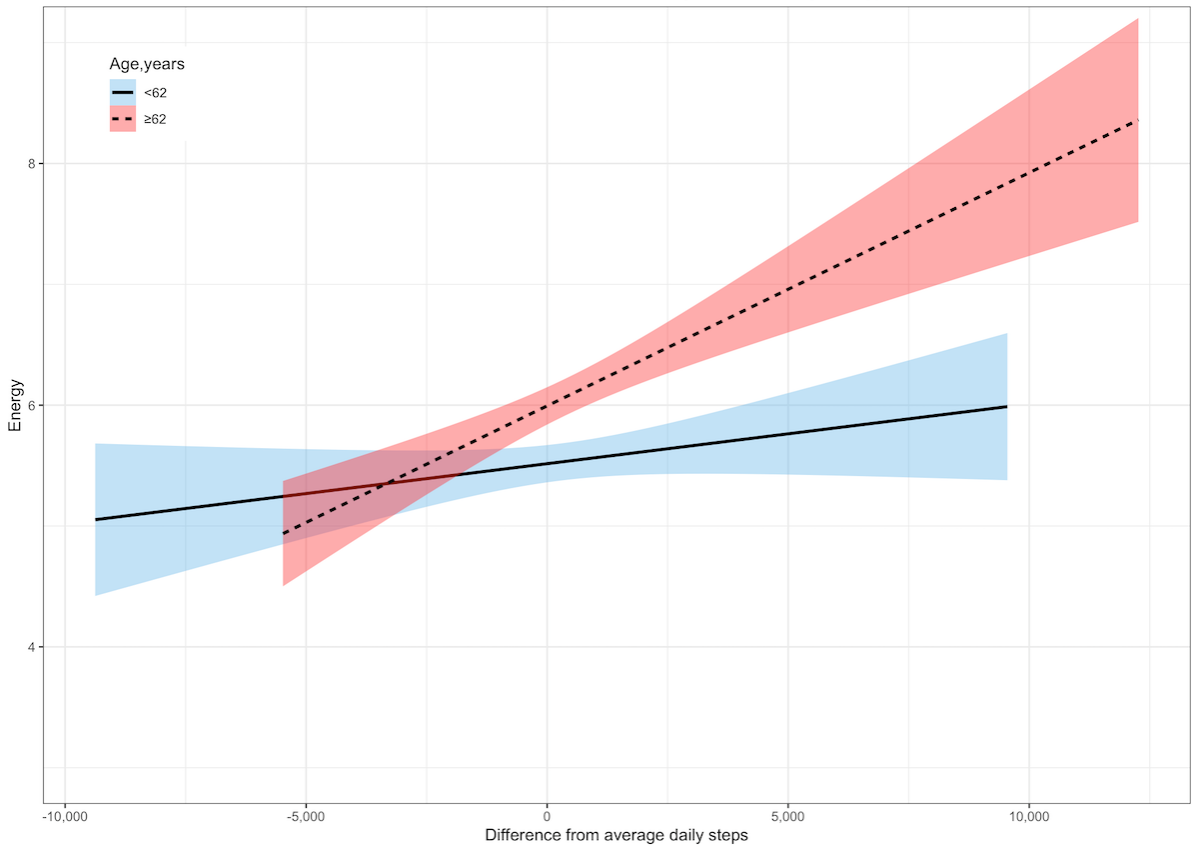
Within-person relationships between daily steps and energy by age. A score of 0 denotes within-person average daily steps. Shaded areas represent 95% CIs.

After adjusting for covariates, on the days the participants took more steps than average, they reported having more energy (γ=0.11; *P*<.001; Table 5). There was a significant interaction between daily steps and sex (γ=0.15; *P*<.001). The relationship between daily steps and energy was significant for women (γ=0.16; *P*<.001) but not for men (γ=0.009; *P*=.68; Figure 6). There was also a significant interaction between daily steps and age (γ=0.005; *P*=.01). The relationship between daily steps and energy was stronger for adults aged ≥62 years (γ=0.16; *P*<.001) than it was for those aged ≤62 years (γ=0.05; *P*=.02; Figure 7).

**Table 5:** Unstandardized coefficients from multilevel effects of daily steps on same-day energy (N=79)^a^.

Outcome	Model 1^b^: energy				Model 2^c^: energy				Model 3^d^: energy		
	β	SE	*P* value	β		SE	*P* value	β		SE	*P* value
Intercept	2.93	1.71	.1	3.02		1.71	.08	2.99		1.71	.08
Daily steps	.11	0.02	<.001	–.14		0.06	.02	–.25		0.14	.07
Age (years)	–.002	0.02	.94	–.0009		0.02	.97	–.003		0.02	.9
Sex	–.62	0.36	.09	–.67		0.36	.07	–.62		0.36	.1
Condition	–.13	0.33	.7	–.11		0.33	.73	–.12		0.33	.71
Education	.09	0.06	.15	.09		0.06	.16	.09		0.06	.15
Health	.04	0.01	<.001	.04		0.01	<.001	.04		0.01	<.001
Average steps	–.01	0.07	.88	–.01		0.07	.84	–.01		0.07	.89
Daily steps×sex	—^e^	—	—	.15		0.04	<.001	—^e^		—	—
Daily steps×age	—^e^	—	—	—^e^		—	—	.006		0.002	.01

^a^Daily steps were rescaled by dividing the number of steps by 1000; therefore, the β value reflects change in energy per 1000-step increase.

^b^Model 1: level 1 variance=1.88 (SD 1.37); level 2 variance=2.06 (SD 1.44). Akaike information criterion=5303.3; Bayesian information criterion=5355.8; log likelihood=–2641.6.

^c^Model 2: level 1 variance=1.87 (SD 1.37); level 2 variance=2.04 (SD 1.43). Akaike information criterion=5286.9; Bayesian information criterion=5344.7; log likelihood=–2638.4.

^d^Model 3: level 1 variance=1.88 (SD 1.37); level 2 variance=2.05 (SD 1.43). Akaike information criterion=5298.7; Bayesian information criterion=5356.6; log likelihood=–2638.4.

^e^Outcome was not included in the model.

#### Lagged Analyses

The effects of steps on next-day mood and energy were also tested. In these analyses, prior-day mood or energy was controlled to determine whether prior-day steps predicted the participants’ mood and energy on the following day above and beyond how they felt on the previous day. After controlling for age, sex, condition, education, health, prior-day mood, and average monthly steps, we found that prior-day steps significantly predicted next-day mood (γ=0.04; *P*=.01; Table 6). There was also a significant interaction between prior-day steps and sex (γ=0.08; *P*=.02). This relationship was significant for women (γ=0.06; *P*=.002) but not for men (γ=–0.007; *P*=.70). There was no significant interaction between prior-day steps and age in predicting next-day mood, unlike our findings in same-day relationships.

**Table 6:** Unstandardized coefficients from multilevel effects of daily steps on lagged (next-day) mood (N=75)^a^.

Outcome	Model 1^b^: next-day mood				Model 2^c^: next-day mood				Model 3^d^: next-day mood		
	β	SE	*P* value	β		SE	*P* value	β		SE	*P* value
Intercept	1.89	1.42	.19	1.99		1.43	.17	1.90		1.42	.19
Prior-day steps	.04	0.02	.01	–.08		0.06	.14	–.005		0.01	.97
Prior-day mood	.30	0.03	<.001	.30		0.03	<.001	.30		0.03	<.001
Age (years)	.04	0.02	.05	.04		0.02	.05	.04		0.02	.06
Sex	–.42	0.30	.17	–.44		0.30	.15	–.42		0.30	.17
Condition	–.44	0.27	.11	–.44		0.28	.12	–.44		0.28	.11
Education	.03	0.05	.58	.03		0.05	.61	.03		0.05	.58
Health	.02	0.01	.04	.02		0.01	.04	.02		0.01	.04
Average steps	–.05	0.06	.38	–.06		0.06	.37	–.05		0.06	.38
Prior-day steps×sex	—^e^	—	—	.08		0.03	.02	—^e^		—	—
Prior-day steps×age	—^e^	—	—	—^e^		—	—	.0007		0.002	.71

^a^Daily steps were rescaled by dividing the number of steps by 1000; therefore, the β value reflects change in mood per 1000-step increase.

^b^Model 1: level 1 variance=1.20 (SD 1.10); level 2 variance=1.48 (SD 1.22). Akaike information criterion=4042.8; Bayesian information criterion=4098.7; log likelihood=–2010.4.

^c^Model 2: level 1 variance=1.22 (SD 1.10); level 2 variance=1.47 (SD 1.21). Akaike information criterion=4039.5; Bayesian information criterion=4100.4; log likelihood=–2007.7.

^d^Model 3: level 1 variance=1.20 (SD 1.10); level 2 variance=1.48 (SD 1.22). Akaike information criterion=4044.7; Bayesian information criterion=4105.6; log likelihood=–2010.3.

^e^Outcome was not included in the model.

Prior-day steps did not predict next-day energy, nor was there a significant prior-day steps×sex or prior-day steps×age interactions in predicting next-day energy (Table 7). The alternative directional relationships were also tested, but prior-day mood and energy did not significantly predict next-day steps.

**Table 7:** Unstandardized coefficients from multilevel effects of daily steps on lagged (next-day) energy (N=75)^a^.

Outcome	Model 1^b^: next-day energy			Model 2^c^: next-day energy				Model 3^d^: next-day energy		
	β	SE	*P* value	β	SE	*P* value	β		SE	*P* value
Intercept	2.01	1.40	.16	1.99	1.40	.16	2.03		1.40	.15
Prior-day steps	.01	0.02	.75	.03	0.07	.71	–.11		0.15	.48
Prior-day energy	–.24	0.03	<.001	.24	0.03	<.001	.24		0.03	<.001
Age (years)	–.001	0.02	.93	–.002	0.02	.93	–.002		0.02	.92
Sex	–.47	0.30	.12	–.46	0.30	.12	–.47		0.30	.12
Condition	–.16	0.27	.55	–.17	0.27	.54	–.16		0.27	.55
Education	.07	0.05	.15	.07	0.05	.15	.07		0.05	.15
Health	.03	0.01	<.001	.03	0.01	<.001	.03		0.01	<.001
Average steps	.01	0.06	.81	.01	0.06	.80	.02		0.06	.80
Prior-day steps×sex	—^e^	—	—	–.01	0.04	.76	—^e^		—	—
Prior-day steps×age	—^e^	—	—	—^e^	—	—	.002		0.002	.45

^a^Daily steps were rescaled by dividing the number of steps by 1000; therefore, the β value reflects change in energy per 1000-step increase.

^b^Model 1: level 1 variance=1.11 (SD 1.06); level 2 variance=2.03 (SD 1.43). Akaike information criterion=4394.8; Bayesian information criterion=4450.7; log likelihood=–2186.4.

^c^Model 2: level 1 variance=1.11 (SD 1.05); level 2 variance=2.03 (SD 1.43). Akaike information criterion=4396.8; Bayesian information criterion=4457.7; log likelihood=–2186.4.

^d^Model 3: level 1 variance=1.12 (SD 1.06); level 2 variance=2.03 (SD 1.43). Akaike information criterion=4396.3; Bayesian information criterion=4457.2; log likelihood=–2186.1.

^e^Outcome was not included in the model.

## Discussion

### Principal Findings

This study was a pilot randomized controlled trial, which examined the effectiveness of StepMATE, a newly developed iPhone app aimed at increasing daily steps in a sample of middle-aged and older adults. The app included behavioral supports, including goal-setting and feedback, action planning, and social supports to encourage changes in behavior. Average daily steps were significantly higher during the 4-week intervention than during the baseline week, and these increases were maintained over the course of the study. The increase in steps, however, did not differ between the 2 groups.

Contrary to the hypotheses, there were no differences in physical activity outcomes between the control condition participants with the basic pedometer-like version of the StepMATE app and the treatment condition participants who had access to the app’s full behavioral strategies. This is likely because the participants in both conditions used the features to a similar extent. Despite having a different version of the app, the treatment condition participants rarely used the additional features available to them. It is possible that the extra supports were not needed or that the participants may have considered the extra features difficult or too time-consuming to use.

It is also possible that self-monitoring and goal-setting are enough to encourage increases in daily walking, as other studies have shown [5,6]. The qualitative feedback from the participants echoes this notion:

I love when I look at my steps for the day and see that I get close or exceed my daily step goals! I am a person who needs to exercise more, and this app reminds me to keep it moving!

Kept track of steps, spot on. Mood questions made me aware of steps, I am checking steps more often and more aware of reaching my goal.

These results are consistent with prior findings that goal-setting is among the most successful behavior change techniques for increasing physical activity [5]. Yet, there was only an initial increase in steps, with no further incremental change throughout the intervention period.

Although there was a significant increase in cognition from pre- to posttest, there were no significant changes for any of the other outcomes, including social engagement, exercise control, or exercise self-efficacy. Although this is consistent with other findings that exercise is associated with improvements in cognition [38,39], the increase in cognitive performance between pre- and posttest could be due to retest effects. The same version of the test was administered on both occasions. We also tested whether a change in steps between baseline and the end of the intervention was correlated with cognitive performance; no significant correlations emerged.

Contrary to the hypotheses, there were no significant improvements in sleep. The PSQI global score at pretest indicated that the participants in general were good sleepers, with an average sleep duration of just under 7 hours and average sleep latencies under 20 minutes. It is possible that a ceiling effect could explain the lack of change in sleep over the 4-week study. To determine whether sleep improved for those with poorer sleep at baseline, post hoc analyses were conducted with a median split of PSQI global scores. There were no changes from pre- to posttest for either good sleepers (PSQI global score of 4 or lower) or those with scores higher than 4. It is also possible that because our pilot study only lasted 4 weeks, it was not long enough to elicit changes in our outcome measures. Future work should assess physical activity and subsequent changes in outcomes over longer time periods, with longer baseline and follow-up periods. This would allow researchers to assess whether changes in physical activity are maintained even after the novelty of a behavior change intervention has worn off.

Although the differences between the conditions were not significant, there was within-person variability over time across the 2 conditions. The within-person hypotheses of daily steps predicting mood and energy were supported and add to prior literature on exercise and affect by suggesting that, similar to findings with more intensive or structured exercise, walking can also elicit mood and energy benefits [18,19]. Although others have shown that the effects of exercise on mood are similar across adulthood [15,40], our findings suggest that there are some differences by age. Furthermore, we provide additional support for prior findings that women seem to experience greater mood and energy benefits of exercise than men [20]. Prior work suggests that women may be more aware of internal states than men; therefore, it is possible that women are more sensitive to changes in mood or energy [41]. In our sample, variations in self-reported mood and energy were higher in women than in men. It is encouraging that these results show that even a low-impact activity such as daily walking can be associated with improvements in self-reported mood and energy, at least for women. It is also promising because those who get more enjoyment out of being active are more likely to continue being active [42]. Walking is an easily accessible form of daily activity, and daily steps are a metric that most American adults can track daily with a smartphone or pedometer.

### Limitations

This study includes some limitations that are worthy of consideration. The app was only available to users of iPhones with step-tracking capabilities; therefore, there may have been selection bias in only recruiting users who have a relatively new iPhone. The generalizability of the study is also limited by a relatively small sample consisting of mostly White, well-educated adults. According to the Pew Research Center, White individuals and those with higher education and higher household income are more likely to be smartphone owners [43]. Of smartphone owners, iPhone owners in particular are more likely to be White, with higher education and income [44]. Although we do have data on whether the participants were working full time or part time or retired, future work could address whether those in certain professions are more or less likely to engage in physical activity. This could aid in the development of targeted interventions for groups that are most inactive. Another limitation is that we did not assess whether the participants were using fitness technology or apps before enrolling in our study. We specifically recruited individuals who believed that they needed to increase their physical activity; therefore, it is likely that even if the participants used these devices in the past, they were not successful in changing long-term behaviors.

As this study was conducted on a rolling basis over the course of a year at different locations, it is possible that seasonal or geographical factors may have played a role in the findings. The validity of the baseline week steps is also unclear. It is possible that the participants’ steps during the week before the intervention may not be representative of their typical daily walking. During the 4-week intervention, the participants were given a pouch for their phone and were specifically asked to carry their phone with them during the day. They were not explicitly instructed to do so before the intervention began. Of the 87 participants, 11 (13%) did not have step-tracking enabled on their iPhone before the study; therefore, they did not have any baseline data. These participants were still included in all analyses because they had step data during the intervention. Post hoc sensitivity analyses revealed that the results did not change if these participants were excluded.

Future studies should aim to collect baseline data for longer periods to obtain a more accurate estimate of normative physical activity levels before an intervention. The study itself was short; 1 month may not be long enough to observe changes in physical activity. Future work could examine whether there is a threshold of intervention duration that must be met to observe physical activity increases. Follow-up assessments after the interventions are completed would also enable examination of long-term benefits and maintenance of any effects.

Measuring physical activity with a smartphone poses limitations. First, the accuracy of measurement may be a limitation. Although some studies and meta-analyses suggest that smartphones—and iPhones in particular—provide accurate and valid measures, especially in terms of differentiating walking from sedentary behaviors [45,46], others suggest that iPhones may be prone to underestimating steps [47]. There could also be accuracy differences based on the iPhone model. The participants may have forgotten to carry their phones with them at different points through the day. It is possible that the participants could have given their phones to others to increase their step counts. The qualitative feedback from the participants suggests that most of them kept their phones on their person for most of the day. As the participants kept their phones with them throughout the day, many of the steps may not have been taken with the intention of walking for exercise. The goal was to capture a full picture of daily activity in our study because walking is an exercise modality that can easily be incorporated into one’s regular routine throughout the day. Thus, we did not differentiate whether the steps were taken for exercise.

The intervention was personalized by allowing the participants to use the app at their convenience and to set and change their walking goals as often as desired. This was designed to mirror what would happen if an individual independently downloaded a new walking app and used it on their own. It is possible that the participants did not use the features of the app because they were not specifically asked to do so. In contrast, in another study that used similar behavioral features [13], the participants were reminded daily to use the calendars and maps and to set goals. In this study, the participants also may not have set their step goals high enough to challenge themselves or encourage increases in walking. Future studies should continue to examine which behavioral supports are most successful in increasing physical activity in older adult populations and find best practices for incorporating these supports into successful physical activity interventions.

Finally, the participants could have encountered some difficulties in using the app and might have preferred a lower-tech intervention for increasing steps. Future work should compare how different age groups can be motivated to increase their activity, especially by making technology more user-friendly and age appropriate. Technology has the potential to assess multiple outcomes (eg, health data and EMAs) in real time, such as through a smartphone, a device that most adults already carry around with them daily [48].

### Conclusions

This study tested whether a new walking app, StepMATE, increased daily walking in a sample of inactive older adults. Weekly step averages were significantly higher during the 4-week study than during the baseline week for both intervention groups, and increases were maintained over the course of the 4-week intervention. However, the treatment condition generally did not use the app’s additional behavioral strategies; thus, both conditions used similar app features. The components that were similar in both conditions, including self-monitoring of steps and daily walking goals, may be sufficient to encourage increases in walking without the need for additional supports. We also found a significant increase in cognition over the course of the study. Future studies should explore how to make apps more user-friendly and accessible to older adults. More daily steps were associated with better same-day mood and energy for women—but not for men—and were also associated with better next-day mood for women. Relationships among walking, mood, and energy were more apparent for older participants than for the younger ones. Future work could more closely examine sex and age differences in the relationship among walking, mood, and energy. Such research could uncover which features of apps are the most successful and motivating for both men and women across adulthood and could lead to the development of large-scale technology-based interventions for increasing physical activity.

## Appendix

Multimedia Appendix 1 Tables and figures from supplementary analyses. Descriptions of different versions of the app.

Multimedia Appendix 2 R codes for pre-post analyses.

Multimedia Appendix 3 R codes for daily analyses.

Multimedia Appendix 4 StepMATE demonstration: control group.

Multimedia Appendix 5 StepMATE demonstration: treatment group.

Multimedia Appendix 6 CONSORT-eHEALTH checklist (V 1.6.1).

## Abbreviations

BTACT: Brief Test of Adult Cognition by Telephone
EMA: ecological momentary assessment
PSQI: Pittsburgh Sleep Quality Index
