# Supplementary material for: Walking and Daily Affect Among Sedentary Older Adults Measured Using the StepMATE App: Pilot Randomized Controlled Trial
Source: JMIR Mhealth Uhealth. 2021 Dec 1;9(12):e27208. doi: 10.2196/27208 (PMC8686479; doi:10.2196/27208)
Supplement: Multimedia Appendix 1 [file mhealth_v9i12e27208_app1.docx]

Supplementary Tables and Figures


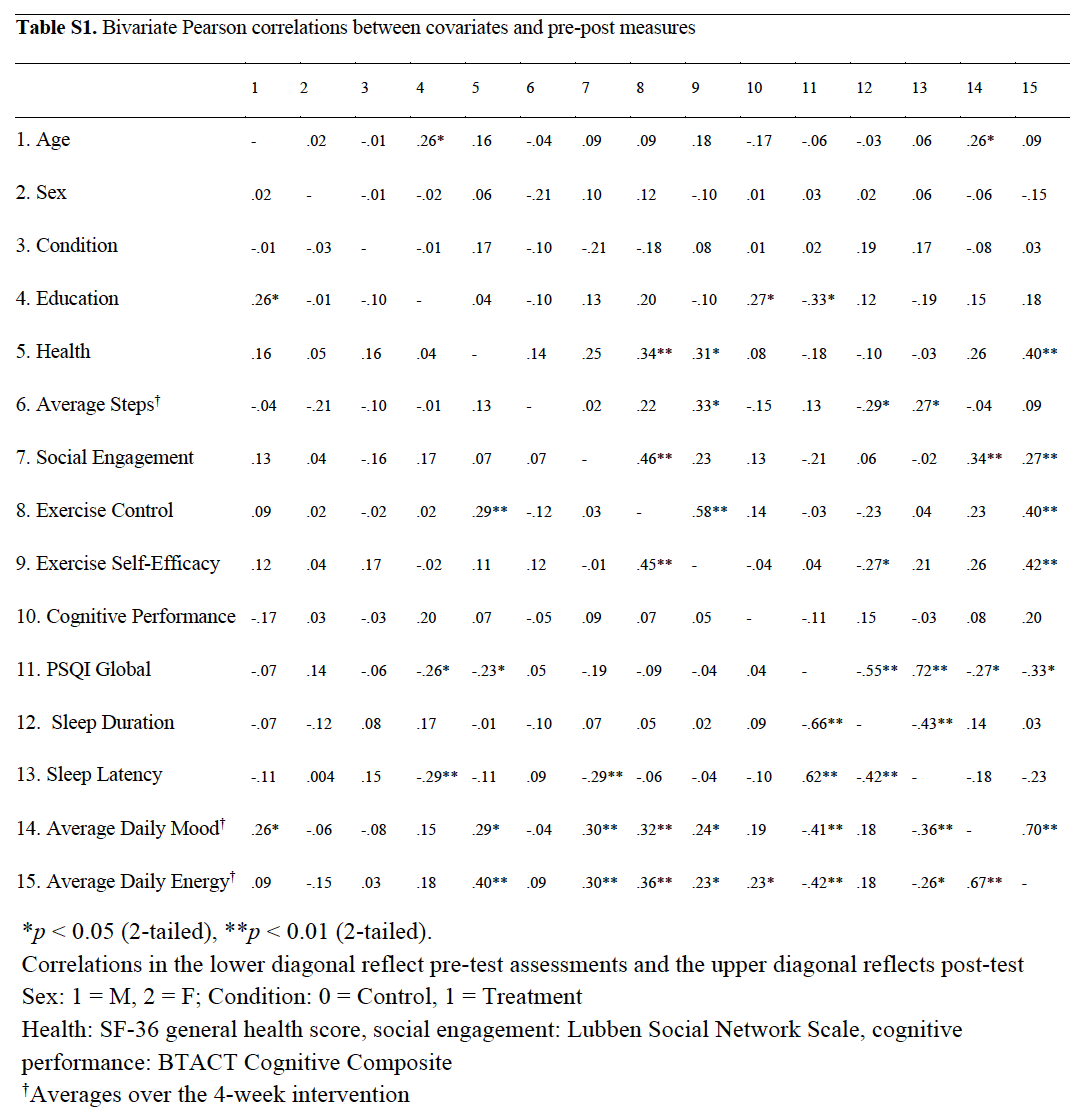


Note: Exact *p*-values are excluded from Supplementary Table 1 for clarity

| Table S2.  Unstandardized coefficients from multilevel time*condition interaction on pre/post outcomes (n=87) | | | | | | | | | | | | |
| --- | --- | --- | --- | --- | --- | --- | --- | --- | --- | --- | --- | --- |
|  | PSQI Global | | | Sleep Duration | | | Sleep Latency | | | Memory | | |
|  | *B* | *SE* | *p* | *B* | *SE* | *p* | *B* | *SE* | *p* | *B* | *SE* | *p* |
|  |  |  |  |  |  |  |  |  |  |  |  |  |
| Intercept | 14.05^***^ | 4.07 | <.001 | 5.93^***^ | 1.17 | <.001 | 64.39^**^ | 23.03 | .006 | -0.17 | 0.51 | .745 |
| Time | 0.11 | 0.43 | .802 | 0.02 | 0.14 | .874 | 0.69 | 2.62 | .792 | 0.17* | 0.07 | .023 |
| Age | 0.007 | 0.06 | .900 | -0.01 | 0.02 | .713 | 0.001 | 0.31 | .998 | -0.02** | 0.01 | .009 |
| Sex | 1.08 | 0.84 | .200 | -0.22 | 0.24 | .362 | 1.80 | 4.74 | .704 | 0.03 | 0.10 | .754 |
| Condition | -0.71 | 1.13 | .531 | 0.27 | 0.34 | .439 | 1.70 | 6.69 | .800 | -0.01 | 0.17 | .943 |
| Education | -0.45^**^ | 0.16 | .006 | 0.09^*^ | 0.05 | .042 | -2.27^*^ | 0.90 | .014 | 0.05* | 0.02 | .015 |
| Health | -0.06^*^ | 0.02 | .016 | 0.0004 | 0.007 | .946 | -0.19 | 0.13 | .154 | 0.003 | 0.003 | .303 |
| Days of app use | 0.02 | 0.05 | .672 | -0.007 | 0.01 | .639 | 0.02 | 0.29 | .949 | 0.005 | 0.006 | .458 |
| Time*Condition | 0.45 | 0.60 | .457 | -0.02 | 0.19 | .928 | 4.64 | 3.68 | .212 | -0.03 | 0.10 | .810 |
| **Variance Components:** *Variance (SD)* | | | | | | | | | | | | |
| Level 1 variance | 11.27 | (3.36) |  | 0.89 | (0.95) |  | 350.5 | (18.72) |  | 0.15 | (0.38) |  |
| Level 2 variance | 2.71 | (1.65) |  | 0.27 | (0.52) |  | 101.8 | (10.09) |  | 0.08 | (0.29) |  |
| AIC | 755.0 |  |  | 404.7 |  |  | 1266.9 |  |  | 189.8 |  |  |
| BIC | 787.8 |  |  | 437.5 |  |  | 1299.6 |  |  | 222.6 |  |  |
| Log likelihood | -366.5 |  |  | -191.4 |  |  | -622.4 |  |  | -83.9 |  |  |
| **p* < 0.05 (2-tailed), ***p* < 0.01 (2-tailed), ****p* < 0.001 (2-tailed). | | | | | | | | | | | | |

| Table S3.  Unstandardized coefficients from multilevel time*condition interaction on pre/post outcomes (n=87) | | | | | | | | | |
| --- | --- | --- | --- | --- | --- | --- | --- | --- | --- |
|  | Social Engagement | | | Exercise Control | | | Exercise Self-Efficacy | | |
| Outcome | *B* | *SE* | *p* | *B* | *SE* | *p* | *B* | *SE* | *p* |
|  | | | | | | | | | |
| Intercept | 21.72^*^ | 10.20 | .036 | 3.32^***^ | 0.55 | <.001 | 2.03^*^ | 0.83 | .017 |
| Time | -0.92 | 1.01 | .362 | -0.16 | 0.11 | .177 | -0.21 | 0.12 | .085 |
| Age | 0.11 | 0.14 | .427 | 0.001 | 0.007 | .864 | 0.01 | 0.01 | .211 |
| Sex | 1.12 | 2.11 | .597 | 0.02 | 0.11 | .862 | -0.09 | 0.17 | .587 |
| Condition | -2.84 | 2.76 | .305 | 0.07 | 0.24 | .774 | 0.31 | 0.28 | .272 |
| Education | 0.44 | 0.40 | .271 | 0.02 | 0.02 | .381 | -0.03 | 0.03 | .433 |
| Health | 0.06 | 0.06 | .310 | 0.01^***^ | 0.003 | <.001 | 0.006 | 0.005 | .209 |
| Days of app use | -0.19 | 0.13 | .141 | 0.002 | 0.007 | .811 | 0.002 | 0.01 | .824 |
| Time *Condition | -0.60 | 1.42 | .676 | -0.15 | 0.16 | .344 | -0.10 | 0.17 | .567 |
| **Variance Components:** *Variance (SD)* | | | | | | | | | |
| Level 1 variance | 72.39 | (8.51) |  | 0.10 | (0.31) |  | 0.39 | (0.63) |  |
| Level 2 variance | 15.02 | (3.88) |  | 0.21 | (0.45) |  | 0.22 | (0.47) |  |
| AIC | 1014.6 |  |  | 254.2 |  |  | 333.6 |  |  |
| BIC | 1047.3 |  |  | 286.9 |  |  | 366.3 |  |  |
| Log likelihood | -496.3 |  |  | -116.1 |  |  | -155.8 |  |  |
| **p* < 0.05 (2-tailed), ***p* < 0.01 (2-tailed), ****p* < 0.001 (2-tailed). | | | | | | | | | |

**StepMATE instructions and screenshots**

**CONTROL CONDITION**

**Home Screen**


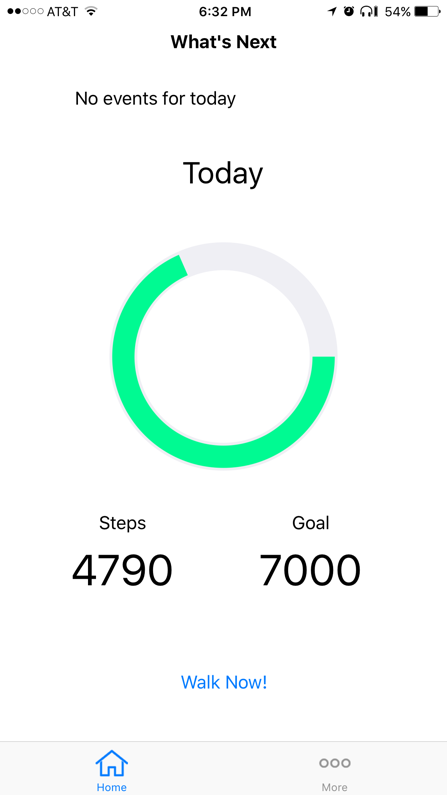


- This is your home screen. It displays how many steps you’ve taken so far today and remind you of what your daily step goal is. The circle represents how active you’ve been today, when the circle is filled, you’ve achieved your daily walking goal
- You can hit ‘Walk now’ to begin a walk
- This screen appears when you click walk now. When you hit start, the app begins tracking the time, distance, and number of steps you’ve taken
- At the end of the walk, hit finish. You’ll get some information about the walk you just took


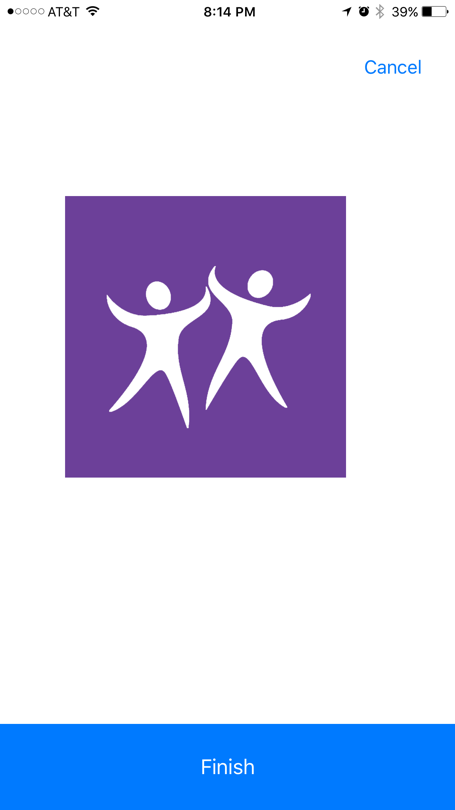


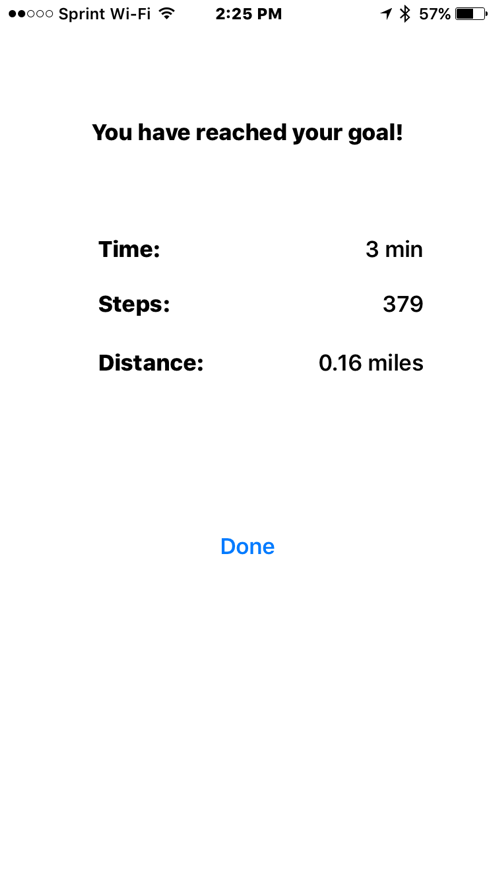


**More**

**
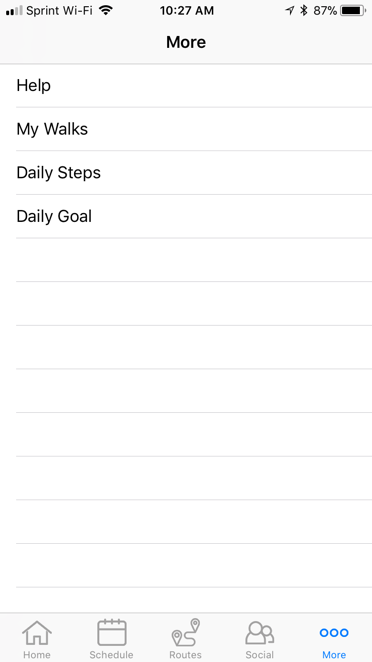
**

- Hit the more icon at the bottom of your home screen
- My Walks: Access previous walks you’ve gone on
- Daily Steps: See how many steps you’ve taken each day you’ve used Step Mate
- Daily goal: You can change your daily walking goal

**Daily Assessments**


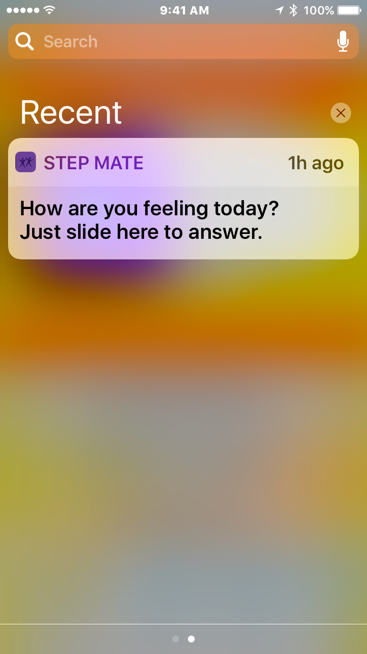


- Twice a day, Step Mate will send you 2 questions about your mood and energy level
-
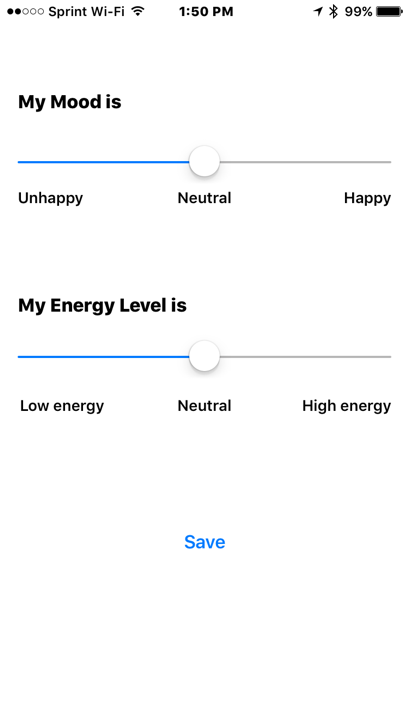
When you get a notification, click on it and it will bring you to the Step Mate app
- Move the slider to enter your mood and energy level
- When you’re done, hit Save
- **NOTE:** If you’ve received a notification and can’t find where it went, try swiping down from the top of your iPhone home screen

**Contact Us**

- If you have any questions about the app, please contact the Step Mate research team by phone or email.

**INTERVENTION CONDITION (NOTE: Some screenshots excluded due to blind review)**

**Home Screen**

-
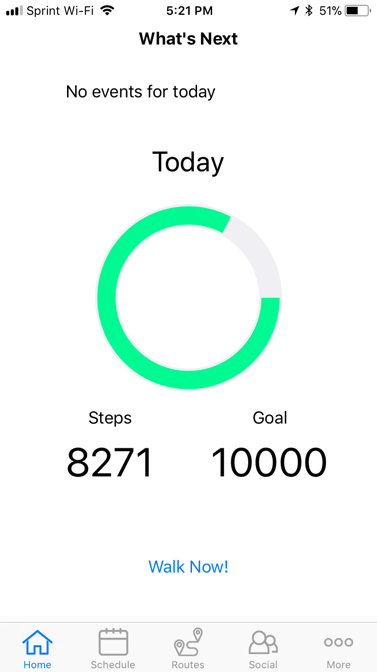
This is your home screen. It displays how many steps you’ve taken so far today and remind you of what your daily step goal is. The circle represents how active you’ve been today, when the circle is filled, you’ve achieved your daily walking goal
- If you have an event scheduled for the day, it will be displayed above the activity circle
- You can hit ‘Walk now’ to begin a walk
- This screen appears when you click walk now. When you hit start, the app begins tracking the time, distance, and number of steps you’ve taken
- At the end of the walk, you’ll be able to name your route so you can easily walk this route again
- You’ll also get some information about the walk you just took


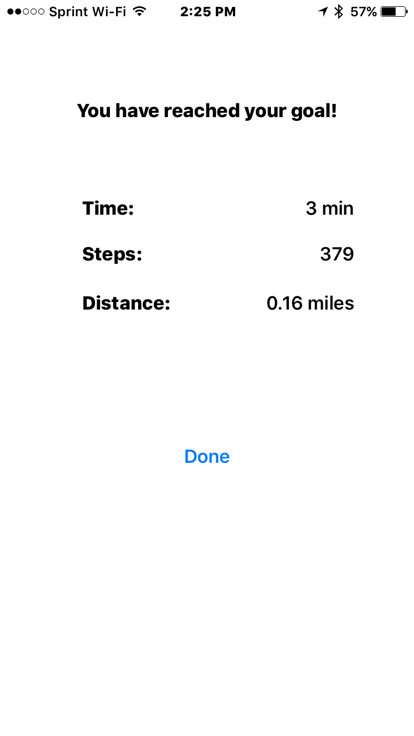


**Schedule**


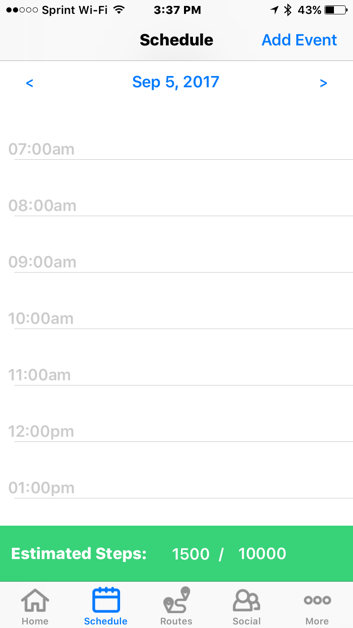


- To look at your schedule, hit the schedule icon at the bottom of the home screen
- To add a new event, hit ‘Add Event’ at the top right
- Write the name of the event, date, start and end time
- Check one of the options if you’d like the event to repeat daily/weekly/monthly
-
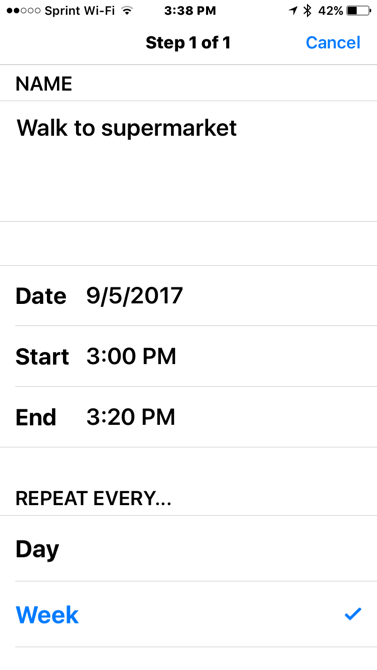
Pick the pace you’ll be walking (normal or brisk)
- Estimate the number of steps you will add based on your previous walks. (**NOTE**: If you do not write in an estimate, the app will estimate your steps automatically based on your typical walking speed and these steps will appear in your iPhone calendar. To get an accurate step estimate, only schedule the time you will be WALKING.)
- Choose whether you’d like a 15-minute reminder
- Hit ‘Done’


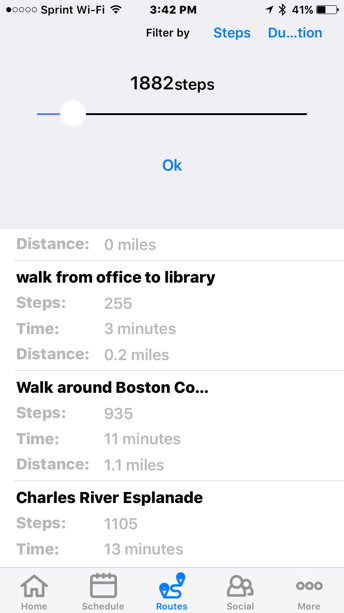
**Routes**

- Hit the routes icon from the home screen to access walking previous routes you’ve walked and named
- You can find the perfect route by filtering the routes by steps or time in the top right
- You can enter how many steps you’d like to add, or how long you’d like to walk

**Social**


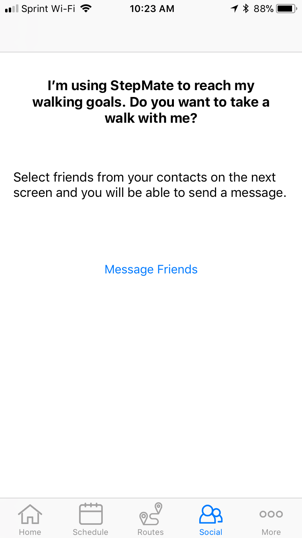


- Hit the social icon at the bottom of your home screen
- You can send a message to your friends to see if they’d like to join you for a walk
- Hit ‘Message Friends’, find a contact you’d like to send a message to, and invite them for a walk


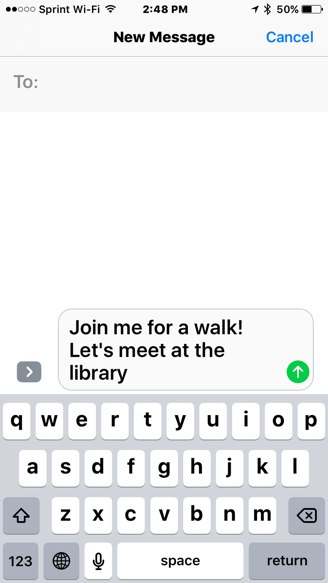


**More**

**
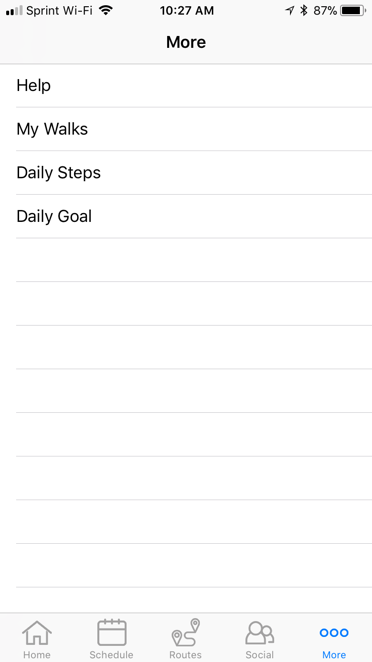
**

- Hit the more icon at the bottom of your home screen
- My Walks: Access previous walks you’ve gone on
- Daily Steps: See how many steps you’ve taken each day you’ve used Step Mate
- Daily goal: You can change your daily walking goal

**Daily Assessments**


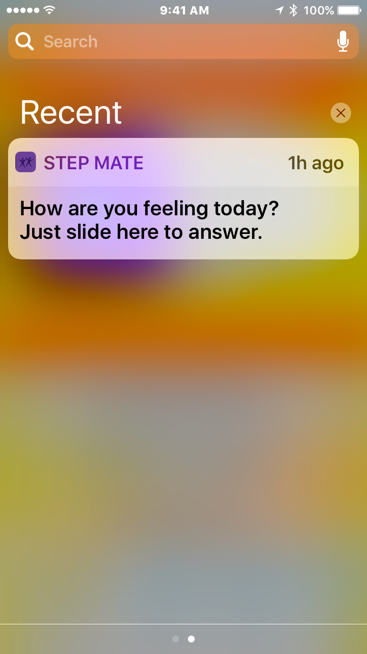


- Twice a day, Step Mate will send you 2 questions about your mood and energy level
-
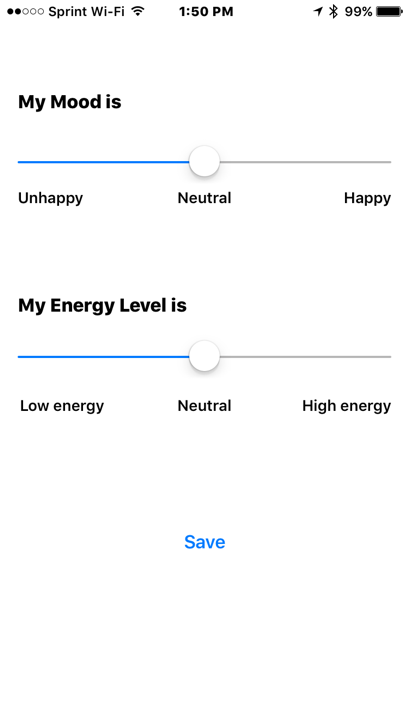
When you get a notification, click on it and it will bring you to the Step Mate app
- Move the slider to enter your mood and energy level
- When you’re done, hit Save
- **NOTE:** If you’ve received a notification and can’t find where it went, try swiping down from the top of your iPhone home screen

**Contact Us**

- If you have any questions about the app, please contact the Step Mate research team by phone or email.
