# Supplementary material for: Walking and Daily Affect Among Sedentary Older Adults Measured Using the StepMATE App: Pilot Randomized Controlled Trial
Source: JMIR Mhealth Uhealth. 2021 Dec 1;9(12):e27208. doi: 10.2196/27208 (PMC8686479; doi:10.2196/27208)
Supplement: Multimedia Appendix 2 [file mhealth_v9i12e27208_app2.pdf]

Pre/post/follow up Questionnaires in MLM

```
library(lme4)
```

```
## Loading required package: Matrix
```

```
library(lmerTest)
```

```
## Registered S3 methods overwritten by 'ggplot2':
##   method         from
##   [.quosures     rlang
##   c.quosures     rlang
##   print.quosures rlang
```

```
##
## Attaching package: 'lmerTest'
```

```
## The following object is masked from 'package:lme4':
##
##     lmer
```

```
## The following object is masked from 'package:stats':
##
##     step
```

```
#install.packages("plotrix")
library(plotrix)
```

```
## Warning: package 'plotrix' was built under R version 3.6.2
```

```
library(psych)
```

```
##
## Attaching package: 'psych'
```

```
## The following object is masked from 'package:plotrix':
##
##     rescale
```

```
setwd("~/Dropbox (Roybal App Study)/Roybal App Study Team Folder/Data/Data/Intent to Tre
at")

##Questionnaires 2 timepoints
itt<-read.csv('QualtricsLong_ITT.csv', header = TRUE, sep = ",")

##daily steps with no baseline
Fullnobase<-read.csv('StepsNoBase.csv', header = TRUE, sep = ",")

##daily steps with baseline
Fullbase<-read.csv('StepsBase.csv', header = TRUE, sep = ",")

(aggregate(Steps ~ Week + Condition, Fullbase,FUN = function(x) c(mean = mean(x), se = s
td.error(x))))
```

```
##    Week Condition Steps.mean  Steps.se
## 1     1         0  4048.1581  224.3855
## 2     2         0  5595.5370  196.2840
## 3     3         0  5109.9569  192.0958
## 4     4         0  5719.1224  210.2036
## 5     5         0  5183.1733  217.5703
## 6     1         1  3457.8776  166.7649
## 7     2         1  5111.2667  193.2026
## 8     3         1  5157.1506  223.3805
## 9     4         1  4938.4260  244.3691
## 10    5         1  5167.0631  270.2104
```

```
aggregate(mood ~ Condition, Fullbase,FUN = function(x) c(mean = mean(x, na.rm=T), sd = s
d(x)))
```

```
##   Condition mood.mean  mood.sd
## 1         0  6.850757 1.994196
## 2         1  6.562025 2.103122
```

```
aggregate(energy ~ Condition, Fullbase,FUN = function(x) c(mean = mean(x, na.rm=T), sd =
sd(x)))
```

```
##   Condition energy.mean energy.sd
## 1         0    5.731013  2.065684
## 2         1    5.801149  2.240449
```

```
mean(Fullbase$mood, na.rm=T)
```

```
## [1] 6.714587
```

```
mean(Fullbase$energy, na.rm=T)
```

```
## [1] 5.76409
```

```
sd(Fullbase$mood, na.rm=T)
```

```
## [1] 2.0507
```

```
sd(Fullbase$energy, na.rm=T)
```

```
## [1] 2.149461
```

```
Fullnobase$StepsRC<-Fullnobase$Steps/1000

Fullbase$StepsRC<-Fullbase$Steps/1000
```

R Markdown

```
m1<-lmer(StepsRC~ Week  + Age + Gender + Condition + EdYrs + T1sf_GeneralHealth + DaysUs
e + Condition*Week + (1|SubjectID), Fullnobase, REML=FALSE)
summary(m1)
```

```
## Linear mixed model fit by maximum likelihood . t-tests use
##   Satterthwaite's method [lmerModLmerTest]
## Formula:
## StepsRC ~ Week + Age + Gender + Condition + EdYrs + T1sf_GeneralHealth +
##     DaysUse + Condition * Week + (1 | SubjectID)
##    Data: Fullnobase
##
##      AIC      BIC   logLik deviance df.resid
##   9038.6   9099.8  -4508.3   9016.6     1907
##
## Scaled residuals:
##     Min      1Q  Median      3Q     Max
## -4.2169 -0.6086 -0.1001  0.4970  5.0577
##
## Random effects:
##  Groups    Name        Variance Std.Dev.
##  SubjectID (Intercept) 4.977    2.231
##  Residual              5.671    2.381
## Number of obs: 1918, groups:  SubjectID, 80
##
## Fixed effects:
##                      Estimate Std. Error         df t value Pr(>|t|)
## (Intercept)           5.47443    2.77646   82.39940   1.972   0.0520 .
## Week                 -0.11518    0.07002 1841.53197  -1.645   0.1001
## Age                  -0.01022    0.03530   80.62659  -0.289   0.7730
## Gender               -1.15745    0.55435   80.36995  -2.088   0.0400 *
## Condition            -0.56709    0.63376  156.72590  -0.895   0.3723
## EdYrs                -0.03333    0.10282   80.17840  -0.324   0.7466
## T1sf_GeneralHealth    0.03179    0.01487   81.44265   2.137   0.0356 *
## DaysUse               0.04439    0.05198   82.55777   0.854   0.3955
## Week:Condition        0.02299    0.10002 1847.40998   0.230   0.8182
## ---
## Signif. codes:  0 '***' 0.001 '**' 0.01 '*' 0.05 '.' 0.1 ' ' 1
##
## Correlation of Fixed Effects:
##             (Intr) Week   Age    Gender Condtn EdYrs  T1s_GH DaysUs
## Week        -0.076
## Age         -0.515 -0.009
## Gender      -0.297  0.003 -0.045
## Condition   -0.203  0.378  0.009  0.048
## EdYrs       -0.375  0.001 -0.222  0.018  0.033
## T1sf_GnrlHl -0.202 -0.004 -0.151 -0.073 -0.143 -0.016
## DaysUse     -0.315 -0.008 -0.145  0.013  0.196 -0.132  0.003
## Week:Condtn  0.075 -0.700 -0.004  0.005 -0.537 -0.009  0.004 -0.017
```

```
anova(m1)
```

```
## Type III Analysis of Variance Table with Satterthwaite's method
##                     Sum Sq Mean Sq NumDF   DenDF F value  Pr(>F)  
## Week               15.3455 15.3455     1 1841.53  2.7060 0.10014  
## Age                 0.4748  0.4748     1   80.63  0.0837 0.77305  
## Gender             24.7225 24.7225     1   80.37  4.3595 0.03997 *
## Condition           4.5406  4.5406     1  156.73  0.8007 0.37226  
## EdYrs               0.5961  0.5961     1   80.18  0.1051 0.74663  
## T1sf_GeneralHealth 25.9065 25.9065     1   81.44  4.5683 0.03557 *
## DaysUse             4.1369  4.1369     1   82.56  0.7295 0.39552  
## Week:Condition      0.2996  0.2996     1 1847.41  0.0528 0.81825  
## ---
## Signif. codes:  0 '***' 0.001 '**' 0.01 '*' 0.05 '.' 0.1 ' ' 1
```

```
m2<-lmer(StepsRC~ Week  + Age + Gender + Condition + EdYrs + T1sf_GeneralHealth + DaysUs
e + Condition*Week + (1|SubjectID), Fullbase, REML=FALSE)
summary(m2)
```

```
## Linear mixed model fit by maximum likelihood . t-tests use
##   Satterthwaite's method [lmerModLmerTest]
## Formula:
## StepsRC ~ Week + Age + Gender + Condition + EdYrs + T1sf_GeneralHealth +
##     DaysUse + Condition * Week + (1 | SubjectID)
##    Data: Fullbase
##
##      AIC      BIC   logLik deviance df.resid
##  11319.5  11383.1  -5648.8  11297.5     2371
##
## Scaled residuals:
##     Min      1Q  Median      3Q     Max
## -3.8483 -0.6166 -0.0890  0.5408  5.7116
##
## Random effects:
##  Groups    Name        Variance Std.Dev.
##  SubjectID (Intercept) 4.442    2.108
##  Residual              6.054    2.461
## Number of obs: 2382, groups:  SubjectID, 80
##
## Fixed effects:
##                     Estimate Std. Error         df t value Pr(>|t|)
## (Intercept)          5.13028    2.61287   81.13295   1.963   0.0530 .
## Week                 0.24230    0.05205 2306.55250   4.655 3.42e-06 ***
## Age                 -0.01590    0.03332   80.27145  -0.477   0.6345
## Gender              -1.13174    0.52328   80.08758  -2.163   0.0335 *
## Condition           -0.51392    0.55054  112.74708  -0.933   0.3526
## EdYrs               -0.03417    0.09702   79.79233  -0.352   0.7256
## T1sf_GeneralHealth   0.02612    0.01402   80.68322   1.863   0.0661 .
## DaysUse              0.03182    0.04894   81.46865   0.650   0.5174
## Week:Condition       0.02271    0.07433 2310.43994   0.306   0.7600
## ---
## Signif. codes:  0 '***' 0.001 '**' 0.01 '*' 0.05 '.' 0.1 ' ' 1
##
## Correlation of Fixed Effects:
##             (Intr) Week   Age    Gender Condtn EdYrs  T1s_GH DaysUs
## Week        -0.049
## Age         -0.516 -0.008
## Gender      -0.300  0.003 -0.043
## Condition   -0.198  0.280  0.007  0.053
## EdYrs       -0.376  0.000 -0.222  0.017  0.034
## T1sf_GnrlHl -0.202 -0.004 -0.151 -0.072 -0.156 -0.017
## DaysUse     -0.313 -0.006 -0.147  0.014  0.211 -0.132  0.003
## Week:Condtn  0.051 -0.700 -0.001  0.004 -0.399 -0.007  0.006 -0.016
```

```
anova(m2)
```

```
## Type III Analysis of Variance Table with Satterthwaite's method
##                      Sum Sq Mean Sq NumDF   DenDF F value    Pr(>F)    
## Week                131.205 131.205     1 2306.55 21.6721 3.418e-06 ***
## Age                   1.379   1.379     1   80.27  0.2277   0.63450    
## Gender               28.319  28.319     1   80.09  4.6776   0.03354 *  
## Condition             5.275   5.275     1  112.75  0.8714   0.35257    
## EdYrs                 0.751   0.751     1   79.79  0.1240   0.72562    
## T1sf_GeneralHealth  21.018  21.018     1   80.68  3.4717   0.06607 .  
## DaysUse               2.560   2.560     1   81.47  0.4228   0.51736    
## Week:Condition        0.565   0.565     1 2310.44  0.0934   0.75999    
## ---
## Signif. codes:  0 '***' 0.001 '**' 0.01 '*' 0.05 '.' 0.1 ' ' 1
```

```
m3<-lmer(BTACT_COMP~Time  + Age + Gender + Condition + EdYrs + T1sf_GeneralHealth + Days
Use + Condition*Time + (1|SubjectID), itt, REML=FALSE)
summary(m3)
```

```
## Linear mixed model fit by maximum likelihood . t-tests use
##   Satterthwaite's method [lmerModLmerTest]
## Formula:
## BTACT_COMP ~ Time + Age + Gender + Condition + EdYrs + T1sf_GeneralHealth +
##     DaysUse + Condition * Time + (1 | SubjectID)
##    Data: itt
##
##      AIC      BIC   logLik deviance df.resid
##    189.8    222.6    -83.9    167.8      134
##
## Scaled residuals:
##      Min       1Q   Median       3Q      Max
## -1.82437 -0.49172 -0.04002  0.49257  2.05015
##
## Random effects:
##  Groups    Name        Variance Std.Dev.
##  SubjectID (Intercept) 0.14628  0.3825
##  Residual              0.08322  0.2885
## Number of obs: 145, groups:  SubjectID, 87
##
## Fixed effects:
##                     Estimate Std. Error         df t value Pr(>|t|)
## (Intercept)        -0.165266   0.507512  87.448912  -0.326  0.74547
## Time                0.172519   0.074155  58.735430   2.326  0.02346 *
## Age                -0.018384   0.006833  81.151488  -2.691  0.00866 **
## Gender              0.032566   0.103736  81.191715   0.314  0.75438
## Condition          -0.012417   0.172048 110.867517  -0.072  0.94259
## EdYrs               0.048815   0.019706  81.780086   2.477  0.01530 *
## T1sf_GeneralHealth  0.003006   0.002900  84.802122   1.036  0.30297
## DaysUse             0.004799   0.006444  97.451059   0.745  0.45821
## Time:Condition     -0.025117   0.104048  60.560373  -0.241  0.81006
## ---
## Signif. codes:  0 '***' 0.001 '**' 0.01 '*' 0.05 '.' 0.1 ' ' 1
##
## Correlation of Fixed Effects:
##             (Intr) Time   Age    Gender Condtn EdYrs  T1s_GH DaysUs
## Time        -0.123
## Age         -0.531 -0.049
## Gender      -0.304  0.007 -0.035
## Condition   -0.188  0.578  0.007 -0.004
## EdYrs       -0.455 -0.003 -0.216  0.031  0.080
## T1sf_GnrlHl -0.237 -0.023 -0.153 -0.073 -0.112 -0.020
## DaysUse     -0.062 -0.088 -0.270 -0.016  0.016 -0.054  0.080
## Time:Condtn  0.143 -0.703 -0.001  0.020 -0.824 -0.040  0.038  0.007
```

```
anova(m3)
```

```
## Type III Analysis of Variance Table with Satterthwaite's method
##                       Sum Sq Mean Sq NumDF   DenDF F value   Pr(>F)
## Time                 0.45044 0.45044     1  58.735  5.4124 0.023465 *
## Age                  0.60246 0.60246     1  81.151  7.2389 0.008659 **
## Gender               0.00820 0.00820     1  81.192  0.0986 0.754380
## Condition            0.00043 0.00043     1 110.868  0.0052 0.942594
## EdYrs                0.51068 0.51068     1  81.780  6.1362 0.015305 *
## T1sf_GeneralHealth 0.08939 0.08939     1  84.802  1.0741 0.302971
## DaysUse              0.04616 0.04616     1  97.451  0.5547 0.458205
## Time:Condition       0.00485 0.00485     1  60.560  0.0583 0.810059
## ---
## Signif. codes:  0 '***' 0.001 '**' 0.01 '*' 0.05 '.' 0.1 ' ' 1
```

```
m4<-lmer(LSNS_COMP~Time  + Age + Gender + Condition + EdYrs + T1sf_GeneralHealth + DaysU
se + Condition*Time + (1|SubjectID), itt, REML=FALSE)
summary(m4)
```

```
## Linear mixed model fit by maximum likelihood . t-tests use
##   Satterthwaite's method [lmerModLmerTest]
## Formula:
## LSNS_COMP ~ Time + Age + Gender + Condition + EdYrs + T1sf_GeneralHealth +
##     DaysUse + Condition * Time + (1 | SubjectID)
##    Data: itt
##
##      AIC      BIC   logLik deviance df.resid
##   1014.6   1047.3   -496.3    992.6      134
##
## Scaled residuals:
##      Min       1Q   Median       3Q      Max
## -1.76159 -0.45756  0.08327  0.43695  2.01729
##
## Random effects:
##  Groups    Name        Variance Std.Dev.
##  SubjectID (Intercept) 72.39    8.508
##  Residual              15.02    3.876
## Number of obs: 145, groups:  SubjectID, 87
##
## Fixed effects:
##                     Estimate Std. Error        df t value Pr(>|t|)
## (Intercept)         21.71888   10.20259  89.61114   2.129    0.036 *
## Time                -0.92472    1.00774  60.83815  -0.918    0.362
## Age                  0.11067    0.13870  86.99416   0.798    0.427
## Gender               1.11677    2.10553  87.02270   0.530    0.597
## Condition           -2.83753    2.75756 141.62963  -1.029    0.305
## EdYrs                0.44231    0.39955  87.34797   1.107    0.271
## T1sf_GeneralHealth   0.05971    0.05852  88.72664   1.020    0.310
## DaysUse             -0.18939    0.12762  93.89680  -1.484    0.141
## Time:Condition      -0.59634    1.41998  61.69761  -0.420    0.676
## ---
## Signif. codes:  0 '***' 0.001 '**' 0.01 '*' 0.05 '.' 0.1 ' ' 1
##
## Correlation of Fixed Effects:
##             (Intr) Time   Age    Gender Condtn EdYrs  T1s_GH DaysUs
## Time        -0.081
## Age         -0.537 -0.034
## Gender      -0.318  0.004 -0.028
## Condition   -0.156  0.490  0.007  0.002
## EdYrs       -0.456 -0.002 -0.219  0.032  0.085
## T1sf_GnrlHl -0.240 -0.016 -0.154 -0.065 -0.126 -0.024
## DaysUse     -0.050 -0.059 -0.289 -0.013  0.021 -0.056  0.087
## Time:Condtn  0.096 -0.705 -0.001  0.015 -0.696 -0.028  0.027  0.005
```

```
anova(m4)
```

```
## Type III Analysis of Variance Table with Satterthwaite's method
##                     Sum Sq Mean Sq NumDF   DenDF F value Pr(>F)
## Time                12.649  12.649     1  60.838  0.8420 0.3624
## Age                  9.563   9.563     1  86.994  0.6366 0.4271
## Gender               4.226   4.226     1  87.023  0.2813 0.5972
## Condition           15.906  15.906     1 141.630  1.0588 0.3052
## EdYrs               18.409  18.409     1  87.348  1.2255 0.2713
## T1sf_GeneralHealth 15.640  15.640     1  88.727  1.0412 0.3103
## DaysUse             33.083  33.083     1  93.897  2.2023 0.1412
## Time:Condition       2.649   2.649     1  61.698  0.1764 0.6760
```

```
m5<-lmer(ExCon_Avg~Time  + Age + Gender + Condition + EdYrs + T1sf_GeneralHealth + DaysU
se + Condition*Time + (1|SubjectID), itt, REML=FALSE)
summary(m5)
```

```
## Linear mixed model fit by maximum likelihood . t-tests use
##   Satterthwaite's method [lmerModLmerTest]
## Formula:
## ExCon_Avg ~ Time + Age + Gender + Condition + EdYrs + T1sf_GeneralHealth +
##     DaysUse + Condition * Time + (1 | SubjectID)
##    Data: itt
##
##      AIC      BIC   logLik deviance df.resid
##    254.2    286.9   -116.1    232.2      134
##
## Scaled residuals:
##      Min       1Q   Median       3Q      Max
## -2.25451 -0.43655  0.07497  0.57606  1.70993
##
## Random effects:
##  Groups    Name        Variance Std.Dev.
##  SubjectID (Intercept) 0.09702  0.3115
##  Residual              0.20617  0.4541
## Number of obs: 145, groups:  SubjectID, 87
##
## Fixed effects:
##                     Estimate Std. Error         df t value Pr(>|t|)
## (Intercept)         3.320274   0.550075  95.056751   6.036 3.04e-08 ***
## Time               -0.156127   0.114388  66.207981  -1.365 0.176910
## Age                 0.001245   0.007252  81.056011   0.172 0.864128
## Gender              0.019201   0.110104  80.993195   0.174 0.861995
## Condition           0.069940   0.243184  91.043309   0.288 0.774306
## EdYrs               0.018453   0.020947  81.658645   0.881 0.380951
## T1sf_GeneralHealth   0.010728   0.003112  87.138843   3.448 0.000873 ***
## DaysUse             0.001728   0.007189 114.322866   0.240 0.810501
## Time:Condition     -0.151844   0.159298  69.647345  -0.953 0.343782
## ---
## Signif. codes:  0 '***' 0.001 '**' 0.01 '*' 0.05 '.' 0.1 ' ' 1
##
## Correlation of Fixed Effects:
##             (Intr) Time   Age    Gender Condtn EdYrs  T1s_GH DaysUs
## Time        -0.182
## Age         -0.519 -0.067
## Gender      -0.279  0.012 -0.049
## Condition   -0.239  0.632  0.007 -0.010
## EdYrs       -0.452 -0.004 -0.211  0.029  0.075
## T1sf_GnrlHl -0.231 -0.029 -0.151 -0.087 -0.100 -0.013
## DaysUse     -0.089 -0.124 -0.232 -0.024  0.013 -0.051  0.066
## Time:Condtn  0.206 -0.699 -0.002  0.026 -0.905 -0.051  0.050  0.008
```

```
anova(m5)
```

```
## Type III Analysis of Variance Table with Satterthwaite's method
##                     Sum Sq Mean Sq NumDF   DenDF F value    Pr(>F)
## Time               0.38408 0.38408     1  66.208  1.8629 0.1769102
## Age                0.00608 0.00608     1  81.056  0.0295 0.8641279
## Gender             0.00627 0.00627     1  80.993  0.0304 0.8619945
## Condition          0.01705 0.01705     1  91.043  0.0827 0.7743060
## EdYrs              0.15999 0.15999     1  81.659  0.7760 0.3809509
## T1sf_GeneralHealth 2.45063 2.45063     1  87.139 11.8863 0.0008729 ***
## DaysUse            0.01191 0.01191     1 114.323  0.0578 0.8105010
## Time:Condition     0.18733 0.18733     1  69.647  0.9086 0.3437818
## ---
## Signif. codes:  0 '***' 0.001 '**' 0.01 '*' 0.05 '.' 0.1 ' ' 1
```

```
m6<-lmer(ESE_Avg~Time  + Age + Gender + Condition + EdYrs + T1sf_GeneralHealth + DaysUse
+ Condition*Time + (1|SubjectID), itt, REML=FALSE)
summary(m6)
```

```
## Linear mixed model fit by maximum likelihood . t-tests use
##   Satterthwaite's method [lmerModLmerTest]
## Formula:
## ESE_Avg ~ Time + Age + Gender + Condition + EdYrs + T1sf_GeneralHealth +
##     DaysUse + Condition * Time + (1 | SubjectID)
##    Data: itt
##
##      AIC      BIC   logLik deviance df.resid
##    333.6    366.3   -155.8    311.6      134
##
## Scaled residuals:
##      Min       1Q   Median       3Q      Max
## -2.04295 -0.50393  0.09647  0.48038  2.20923
##
## Random effects:
##  Groups    Name        Variance Std.Dev.
##  SubjectID (Intercept) 0.3934   0.6272
##  Residual              0.2245   0.4739
## Number of obs: 145, groups:  SubjectID, 87
##
## Fixed effects:
##                     Estimate Std. Error         df t value Pr(>|t|)
## (Intercept)         2.034231   0.832661  92.645809   2.443   0.0165 *
## Time               -0.212969   0.121798  64.127337  -1.749   0.0852 .
## Age                 0.014116   0.011210  86.521464   1.259   0.2113
## Gender             -0.092806   0.170190  86.560719  -0.545   0.5869
## Condition           0.311908   0.282484 114.592821   1.104   0.2718
## EdYrs              -0.025450   0.032330  87.135555  -0.787   0.4333
## T1sf_GeneralHealth  0.006017   0.004758  90.079503   1.264   0.2093
## DaysUse             0.002358   0.010573 102.209172   0.223   0.8239
## Time:Condition     -0.098399   0.170893  65.995503  -0.576   0.5667
## ---
## Signif. codes:  0 '***' 0.001 '**' 0.01 '*' 0.05 '.' 0.1 ' ' 1
##
## Correlation of Fixed Effects:
##             (Intr) Time   Age    Gender Condtn EdYrs  T1s_GH DaysUs
## Time        -0.123
## Age         -0.531 -0.049
## Gender      -0.304  0.007 -0.035
## Condition   -0.188  0.579  0.007 -0.004
## EdYrs       -0.455 -0.003 -0.216  0.031  0.080
## T1sf_GnrlHl -0.237 -0.023 -0.153 -0.073 -0.112 -0.020
## DaysUse     -0.062 -0.088 -0.270 -0.016  0.016 -0.054  0.080
## Time:Condtn  0.144 -0.703 -0.001  0.020 -0.825 -0.040  0.038  0.007
```

```
anova(m6)
```

```
## Type III Analysis of Variance Table with Satterthwaite's method
##                      Sum Sq Mean Sq NumDF   DenDF F value  Pr(>F)  
## Time                0.68650 0.68650     1  64.127  3.0574 0.08515 .
## Age                 0.35602 0.35602     1  86.521  1.5856 0.21135  
## Gender              0.06677 0.06677     1  86.561  0.2974 0.58694  
## Condition           0.27375 0.27375     1 114.593  1.2192 0.27184  
## EdYrs               0.13914 0.13914     1  87.136  0.6197 0.43331  
## T1sf_GeneralHealth 0.35902 0.35902     1  90.080  1.5989 0.20932  
## DaysUse             0.01117 0.01117     1 102.209  0.0498 0.82394  
## Time:Condition      0.07444 0.07444     1  65.996  0.3315 0.56671  
## ---
## Signif. codes:  0 '***' 0.001 '**' 0.01 '*' 0.05 '.' 0.1 ' ' 1
```

```
m7<-lmer(SleepDuration~Time  + Age + Gender + Condition + EdYrs + T1sf_GeneralHealth + D
aysUse + Condition*Time + (1|SubjectID), itt, REML=FALSE)
summary(m7)
```

```
## Linear mixed model fit by maximum likelihood . t-tests use
##   Satterthwaite's method [lmerModLmerTest]
## Formula:
## SleepDuration ~ Time + Age + Gender + Condition + EdYrs + T1sf_GeneralHealth +
##     DaysUse + Condition * Time + (1 | SubjectID)
##    Data: itt
##
##      AIC      BIC   logLik deviance df.resid
##    404.7    437.5   -191.4    382.7      134
##
## Scaled residuals:
##     Min      1Q  Median      3Q     Max
## -2.0663 -0.3712  0.0210  0.3507  1.8921
##
## Random effects:
##  Groups    Name        Variance Std.Dev.
##  SubjectID (Intercept) 0.8933   0.9451
##  Residual              0.2736   0.5231
## Number of obs: 145, groups:  SubjectID, 87
##
## Fixed effects:
##                     Estimate Std. Error         df t value Pr(>|t|)
## (Intercept)         5.927303   1.167849  88.954041   5.075  2.1e-06 ***
## Time                0.021506   0.135498  60.214107   0.159   0.8744
## Age                -0.005851   0.015829  85.226927  -0.370   0.7126
## Gender             -0.220100   0.240299  85.262691  -0.916   0.3623
## Condition           0.266617   0.343228 131.656793   0.777   0.4387
## EdYrs               0.094319   0.045616  85.687791   2.068   0.0417 *
## T1sf_GeneralHealth  0.000457   0.006691  87.596707   0.068   0.9457
## DaysUse            -0.006910   0.014677  94.998755  -0.471   0.6388
## Time:Condition     -0.017313   0.190666  61.391599  -0.091   0.9279
## ---
## Signif. codes:  0 '***' 0.001 '**' 0.01 '*' 0.05 '.' 0.1 ' ' 1
##
## Correlation of Fixed Effects:
##             (Intr) Time   Age    Gender Condtn EdYrs  T1s_GH DaysUs
## Time        -0.096
## Age         -0.535 -0.039
## Gender      -0.313  0.005 -0.030
## Condition   -0.167  0.530  0.007  0.000
## EdYrs       -0.456 -0.002 -0.218  0.032  0.083
## T1sf_GnrlHl -0.239 -0.019 -0.154 -0.067 -0.120 -0.023
## DaysUse     -0.054 -0.069 -0.283 -0.014  0.019 -0.055  0.085
## Time:Condtn  0.113 -0.704 -0.001  0.017 -0.753 -0.032  0.031  0.006
```

```
anova(m7)
```

```
## Type III Analysis of Variance Table with Satterthwaite's method
##                      Sum Sq Mean Sq NumDF   DenDF F value  Pr(>F)  
## Time                0.00689 0.00689     1  60.214  0.0252 0.87442  
## Age                 0.03738 0.03738     1  85.227  0.1366 0.71256  
## Gender              0.22953 0.22953     1  85.263  0.8389 0.36228  
## Condition           0.16509 0.16509     1 131.657  0.6034 0.43868  
## EdYrs               1.16969 1.16969     1  85.688  4.2752 0.04169 *
## T1sf_GeneralHealth 0.00128 0.00128     1  87.597  0.0047 0.94570  
## DaysUse             0.06065 0.06065     1  94.999  0.2217 0.63885  
## Time:Condition      0.00226 0.00226     1  61.392  0.0082 0.92794  
## ---
## Signif. codes:  0 '***' 0.001 '**' 0.01 '*' 0.05 '.' 0.1 ' ' 1
```

```
m8<-lmer(SleepLatency~Time  + Age + Gender + Condition + EdYrs + T1sf_GeneralHealth + Da
ysUse + Condition*Time + (1|SubjectID), itt, REML=FALSE)
summary(m8)
```

```
## Linear mixed model fit by maximum likelihood . t-tests use
##   Satterthwaite's method [lmerModLmerTest]
## Formula:
## SleepLatency ~ Time + Age + Gender + Condition + EdYrs + T1sf_GeneralHealth +
##     DaysUse + Condition * Time + (1 | SubjectID)
##    Data: itt
##
##      AIC      BIC   logLik deviance df.resid
##   1266.9   1299.6   -622.4   1244.9      134
##
## Scaled residuals:
##     Min      1Q  Median      3Q     Max
## -2.8235 -0.3072 -0.0568  0.1841  4.0811
##
## Random effects:
##  Groups    Name        Variance Std.Dev.
##  SubjectID (Intercept) 350.5    18.72
##  Residual              101.8    10.09
## Number of obs: 145, groups:  SubjectID, 87
##
## Fixed effects:
##                      Estimate Std. Error         df t value Pr(>|t|)
## (Intercept)         6.439e+01  2.303e+01  8.523e+01   2.797  0.00638 **
## Time                6.933e-01  2.615e+00  5.647e+01   0.265  0.79188
## Age                 9.741e-04  3.122e-01  8.160e+01   0.003  0.99752
## Gender              1.804e+00  4.740e+00  8.164e+01   0.381  0.70452
## Condition           1.699e+00  6.687e+00  1.320e+02   0.254  0.79986
## EdYrs              -2.271e+00  8.997e-01  8.206e+01  -2.525  0.01351 *
## T1sf_GeneralHealth -1.897e-01  1.319e-01  8.392e+01  -1.438  0.15427
## DaysUse             1.854e-02  2.892e-01  9.116e+01   0.064  0.94901
## Time:Condition      4.641e+00  3.681e+00  5.758e+01   1.261  0.21241
## ---
## Signif. codes:  0 '***' 0.001 '**' 0.01 '*' 0.05 '.' 0.1 ' ' 1
##
## Correlation of Fixed Effects:
##             (Intr) Time   Age    Gender Condtn EdYrs  T1s_GH DaysUs
## Time        -0.094
## Age         -0.535 -0.038
## Gender      -0.314  0.005 -0.030
## Condition   -0.166  0.525  0.007  0.000
## EdYrs       -0.456 -0.002 -0.218  0.032  0.083
## T1sf_GnrlHl -0.239 -0.019 -0.154 -0.067 -0.121 -0.023
## DaysUse     -0.053 -0.068 -0.284 -0.014  0.019 -0.055  0.085
## Time:Condtn  0.111 -0.704 -0.001  0.016 -0.746 -0.032  0.031  0.006
```

```
anova(m8)
```

```
## Type III Analysis of Variance Table with Satterthwaite's method
##                     Sum Sq Mean Sq NumDF   DenDF F value  Pr(>F)  
## Time                  7.16    7.16     1  56.473  0.0703 0.79188  
## Age                   0.00    0.00     1  81.604  0.0000 0.99752  
## Gender               14.74   14.74     1  81.640  0.1448 0.70452  
## Condition             6.57    6.57     1 132.043  0.0645 0.79986  
## EdYrs               648.83  648.83     1  82.058  6.3735 0.01351 *
## T1sf_GeneralHealth 210.39  210.39     1  83.921  2.0667 0.15427  
## DaysUse               0.42    0.42     1  91.159  0.0041 0.94901  
## Time:Condition      161.87  161.87     1  57.580  1.5900 0.21241  
## ---
## Signif. codes:  0 '***' 0.001 '**' 0.01 '*' 0.05 '.' 0.1 ' ' 1
```

```
m9<-lmer(PSQIglobal~Time  + Age + Gender + Condition + EdYrs + T1sf_GeneralHealth + Days
Use + Condition*Time + (1|SubjectID), itt, REML=FALSE)
summary(m9)
```

```
## Linear mixed model fit by maximum likelihood . t-tests use
##   Satterthwaite's method [lmerModLmerTest]
## Formula:
## PSQIglobal ~ Time + Age + Gender + Condition + EdYrs + T1sf_GeneralHealth +
##     DaysUse + Condition * Time + (1 | SubjectID)
##    Data: itt
##
##      AIC      BIC   logLik deviance df.resid
##    755.0    787.8   -366.5    733.0      134
##
## Scaled residuals:
##      Min       1Q   Median       3Q      Max
## -2.49664 -0.39054 -0.09391  0.37951  2.63171
##
## Random effects:
##  Groups    Name        Variance Std.Dev.
##  SubjectID (Intercept) 11.266   3.357
##  Residual               2.709   1.646
## Number of obs: 145, groups:  SubjectID, 87
##
## Fixed effects:
##                     Estimate Std. Error         df t value Pr(>|t|)
## (Intercept)        14.048798   4.066291  88.193117   3.455 0.000848 ***
## Time                0.107882   0.427409  59.406560   0.252 0.801596
## Age                 0.006953   0.055223  85.175015   0.126 0.900097
## Gender              1.081538   0.838312  85.206571   1.290 0.200493
## Condition          -0.712334   1.133405 138.248477  -0.628 0.530720
## EdYrs              -0.448473   0.159101  85.570587  -2.819 0.005987 **
## T1sf_GeneralHealth -0.057199   0.023314  87.145045  -2.453 0.016142 *
## DaysUse             0.021671   0.050947  93.128393   0.425 0.671550
## Time:Condition      0.451090   0.601962  60.372131   0.749 0.456547
## ---
## Signif. codes:  0 '***' 0.001 '**' 0.01 '*' 0.05 '.' 0.1 ' ' 1
##
## Correlation of Fixed Effects:
##             (Intr) Time   Age    Gender Condtn EdYrs  T1s_GH DaysUs
## Time        -0.087
## Age         -0.536 -0.036
## Gender      -0.316  0.004 -0.029
## Condition   -0.160  0.506  0.007  0.001
## EdYrs       -0.456 -0.002 -0.219  0.032  0.084
## T1sf_GnrlHl -0.239 -0.017 -0.154 -0.066 -0.123 -0.024
## DaysUse     -0.051 -0.063 -0.287 -0.013  0.020 -0.056  0.086
## Time:Condtn  0.103 -0.705 -0.001  0.015 -0.719 -0.030  0.029  0.005
```

```
anova(m9)
```

```
## Type III Analysis of Variance Table with Satterthwaite's method
##                     Sum Sq Mean Sq NumDF   DenDF F value   Pr(>F)
## Time                0.1726  0.1726     1  59.407  0.0637 0.801596
## Age                 0.0430  0.0430     1  85.175  0.0159 0.900097
## Gender              4.5093  4.5093     1  85.207  1.6645 0.200493
## Condition           1.0701  1.0701     1 138.248  0.3950 0.530720
## EdYrs              21.5259 21.5259     1  85.571  7.9456 0.005987 **
## T1sf_GeneralHealth 16.3072 16.3072     1  87.145  6.0193 0.016142 *
## DaysUse             0.4902  0.4902     1  93.128  0.1809 0.671550
## Time:Condition      1.5213  1.5213     1  60.372  0.5615 0.456547
## ---
## Signif. codes:  0 '***' 0.001 '**' 0.01 '*' 0.05 '.' 0.1 ' ' 1
```
