# Supplementary material for: Walking and Daily Affect Among Sedentary Older Adults Measured Using the StepMATE App: Pilot Randomized Controlled Trial
Source: JMIR Mhealth Uhealth. 2021 Dec 1;9(12):e27208. doi: 10.2196/27208 (PMC8686479; doi:10.2196/27208)
Supplement: Multimedia Appendix 3 [file mhealth_v9i12e27208_app3.pdf]

StepMLMLAG

ABisson

3/24/2020

Same Day mood/energy

```
##predicting same day mood/energy, between- and within - person steps
#mX: origninal model with no interaction
#mXi: Model with step*gender interaction
#mXf: model with only women
#mXm: Model with only men

m1<-lmer(mood~WPstepsRC + PMstepsRC + Gender  + Age  + Condition  + EdYrs  + T1sf_Genera
lHealth  +  (1|SubjectID), stepmate, REML=FALSE)
summary(m1)
```

```
## Linear mixed model fit by maximum likelihood . t-tests use
##   Satterthwaite's method [lmerModLmerTest]
## Formula:
## mood ~ WPstepsRC + PMstepsRC + Gender + Age + Condition + EdYrs +
##     T1sf_GeneralHealth + (1 | SubjectID)
##    Data: stepmate
##
##      AIC      BIC   logLik deviance df.resid
##   4981.2   5033.8  -2480.6   4961.2     1412
##
## Scaled residuals:
##     Min      1Q  Median      3Q     Max
## -5.0412 -0.4353  0.1514  0.5233  2.9898
##
## Random effects:
##  Groups    Name        Variance Std.Dev.
##  SubjectID (Intercept) 2.380    1.543
##  Residual              1.604    1.267
## Number of obs: 1422, groups:  SubjectID, 79
##
## Fixed effects:
##                     Estimate Std. Error         df t value Pr(>|t|)
## (Intercept)          3.17547    1.89555   78.80749   1.675   0.0979 .
## WPstepsRC            0.05896    0.01502 1348.14563   3.926 9.06e-05 ***
## PMstepsRC           -0.08831    0.08206   77.76769  -1.076   0.2852
## Gender              -0.47241    0.40417   78.53709  -1.169   0.2460
## Age                  0.03708    0.02412   78.26466   1.538   0.1282
## Condition           -0.49853    0.36849   78.42901  -1.353   0.1800
## EdYrs                0.04946    0.07042   77.86190   0.702   0.4845
## T1sf_GeneralHealth   0.02657    0.01065   81.00564   2.494   0.0147 *
## ---
## Signif. codes:  0 '***' 0.001 '**' 0.01 '*' 0.05 '.' 0.1 ' ' 1
##
## Correlation of Fixed Effects:
##             (Intr) WPstRC PMstRC Gender Age    Condtn EdYrs
## WPstepsRC    0.003
## PMstepsRC   -0.272 -0.004
## Gender      -0.368 -0.002  0.261
## Age         -0.583 -0.002  0.030 -0.030
## Condition   -0.172  0.005  0.141  0.114  0.048
## EdYrs       -0.437 -0.002  0.026  0.028 -0.242  0.072
## T1sf_GnrlHl -0.136 -0.004 -0.214 -0.134 -0.159 -0.212 -0.021
```

```
m1i<-lmer(mood~WPstepsRC*Gender + PMstepsRC + Age  + Condition  + EdYrs  + T1sf_GeneralH
ealth  +  (1|SubjectID), stepmate, REML=FALSE)
summary(m1i)
```

```
## Linear mixed model fit by maximum likelihood . t-tests use
##   Satterthwaite's method [lmerModLmerTest]
## Formula:
## mood ~ WPstepsRC * Gender + PMstepsRC + Age + Condition + EdYrs +
##     T1sf_GeneralHealth + (1 | SubjectID)
##    Data: stepmate
##
##      AIC      BIC   logLik deviance df.resid
##   4976.8   5034.7  -2477.4   4954.8     1411
##
## Scaled residuals:
##     Min      1Q  Median      3Q     Max
## -4.9978 -0.4504  0.1495  0.5222  3.0310
##
## Random effects:
##  Groups    Name        Variance Std.Dev.
##  SubjectID (Intercept) 2.380    1.543
##  Residual              1.597    1.264
## Number of obs: 1422, groups:  SubjectID, 79
##
## Fixed effects:
##                     Estimate Std. Error         df t value Pr(>|t|)
## (Intercept)          3.22009    1.89514   78.82183   1.699   0.0932 .
## WPstepsRC           -0.06987    0.05299 1346.63451  -1.318   0.1876
## Gender              -0.49552    0.40417   78.61972  -1.226   0.2239
## PMstepsRC           -0.08997    0.08204   77.78162  -1.097   0.2762
## Age                  0.03741    0.02411   78.27131   1.552   0.1248
## Condition           -0.49088    0.36841   78.44178  -1.332   0.1866
## EdYrs                0.04834    0.07041   77.87147   0.687   0.4943
## T1sf_GeneralHealth    0.02649    0.01065   81.00025   2.487   0.0149 *
## WPstepsRC:Gender      0.07883    0.03111 1347.06507   2.534   0.0114 *
## ---
## Signif. codes:  0 '***' 0.001 '**' 0.01 '*' 0.05 '.' 0.1 ' ' 1
##
## Correlation of Fixed Effects:
##             (Intr) WPstRC Gender PMstRC Age    Condtn EdYrs  T1s_GH
## WPstepsRC   -0.008
## Gender      -0.368  0.021
## PMstepsRC   -0.272  0.006  0.261
## Age         -0.583 -0.006 -0.030  0.030
## Condition   -0.172 -0.006  0.113  0.141  0.048
## EdYrs       -0.437  0.005  0.028  0.026 -0.242  0.072
## T1sf_GnrlHl -0.136  0.002 -0.134 -0.214 -0.159 -0.212 -0.021
## WPstpsRC:Gn  0.009 -0.959 -0.023 -0.008  0.005  0.008 -0.006 -0.003
```

```
m1f<-lmer(mood~WPstepsRC + PMstepsRC  + Age  + Condition  + EdYrs  + T1sf_GeneralHealth
+  (1|SubjectID), mystepmateF, REML=FALSE)
summary(m1f)
```

```
## Linear mixed model fit by maximum likelihood . t-tests use
##   Satterthwaite's method [lmerModLmerTest]
## Formula:
## mood ~ WPstepsRC + PMstepsRC + Age + Condition + EdYrs + T1sf_GeneralHealth +
##     (1 | SubjectID)
##    Data: mystepmateF
##
##      AIC      BIC   logLik deviance df.resid
##   3636.7   3680.7  -1809.3   3618.7      976
##
## Scaled residuals:
##     Min      1Q  Median      3Q     Max
## -4.5480 -0.4251  0.1441  0.5550  2.7596
##
## Random effects:
##  Groups    Name        Variance Std.Dev.
##  SubjectID (Intercept) 2.699    1.643
##  Residual              1.934    1.391
## Number of obs: 985, groups:  SubjectID, 55
##
## Fixed effects:
##                      Estimate Std. Error         df t value Pr(>|t|)
## (Intercept)          2.072527   2.211389  55.261133   0.937    0.353
## WPstepsRC            0.087555   0.020704 933.725221   4.229 2.58e-05 ***
## PMstepsRC           -0.009885   0.132742  54.109783  -0.074    0.941
## Age                  0.023565   0.030343  54.197663   0.777    0.441
## Condition           -0.433346   0.471964  54.283905  -0.918    0.363
## EdYrs                0.060096   0.088788  54.120412   0.677    0.501
## T1sf_GeneralHealth   0.032822   0.013215  56.320941   2.484    0.016 *
## ---
## Signif. codes:  0 '***' 0.001 '**' 0.01 '*' 0.05 '.' 0.1 ' ' 1
##
## Correlation of Fixed Effects:
##             (Intr) WPstRC PMstRC Age    Condtn EdYrs
## WPstepsRC    0.001
## PMstepsRC   -0.023 -0.011
## Age         -0.665  0.003  0.021
## Condition   -0.138  0.011  0.161  0.102
## EdYrs       -0.491 -0.005 -0.224 -0.172  0.001
## T1sf_GnrlHl -0.171 -0.005 -0.308 -0.230 -0.239  0.084
```

```
m1m<-lmer(mood~WPstepsRC + PMstepsRC  + Age  + Condition  + EdYrs  + T1sf_GeneralHealth
+  (1|SubjectID), mystepmateM, REML=FALSE)
summary(m1m)
```

```
## Linear mixed model fit by maximum likelihood . t-tests use
##   Satterthwaite's method [lmerModLmerTest]
## Formula:
## mood ~ WPstepsRC + PMstepsRC + Age + Condition + EdYrs + T1sf_GeneralHealth +
##     (1 | SubjectID)
##    Data: mystepmateM
##
##      AIC      BIC   logLik deviance df.resid
##   1256.1   1292.8   -619.1   1238.1      428
##
## Scaled residuals:
##     Min      1Q  Median      3Q     Max
## -3.6818 -0.6103  0.1830  0.4810  2.7088
##
## Random effects:
##  Groups    Name        Variance Std.Dev.
##  SubjectID (Intercept) 1.185    1.0885
##  Residual              0.837    0.9149
## Number of obs: 437, groups:  SubjectID, 24
##
## Fixed effects:
##                     Estimate Std. Error         df t value Pr(>|t|)
## (Intercept)         5.410848   2.526060  24.039089   2.142   0.0425 *
## WPstepsRC           0.008595   0.017935 414.314369   0.479   0.6320
## PMstepsRC          -0.209747   0.087251  23.719939  -2.404   0.0244 *
## Age                 0.069622   0.034903  24.446280   1.995   0.0573 .
## Condition          -0.719908   0.487184  24.547827  -1.478   0.1522
## EdYrs              -0.082533   0.115944  24.222579  -0.712   0.4834
## T1sf_GeneralHealth  0.001134   0.016886  25.366357   0.067   0.9470
## ---
## Signif. codes:  0 '***' 0.001 '**' 0.01 '*' 0.05 '.' 0.1 ' ' 1
##
## Correlation of Fixed Effects:
##             (Intr) WPstRC PMstRC Age    Condtn EdYrs
## WPstepsRC    0.000
## PMstepsRC   -0.399  0.009
## Age         -0.545 -0.012 -0.074
## Condition   -0.110 -0.004  0.187 -0.144
## EdYrs       -0.373  0.012  0.410 -0.453  0.248
## T1sf_GnrlHl -0.344 -0.003 -0.140  0.148 -0.228 -0.237
```

```
m1.age<-lmer(mood~WPstepsRC*Age  + Gender + PMstepsRC +  Condition  + EdYrs  + T1sf_Gene
ralHealth  +  (1|SubjectID), stepmate, REML=FALSE)
summary(m1.age)
```

```
## Linear mixed model fit by maximum likelihood . t-tests use
##   Satterthwaite's method [lmerModLmerTest]
## Formula:
## mood ~ WPstepsRC * Age + Gender + PMstepsRC + Condition + EdYrs +
##     T1sf_GeneralHealth + (1 | SubjectID)
##    Data: stepmate
##
##      AIC      BIC   logLik deviance df.resid
##   4977.7   5035.5  -2477.8   4955.7     1411
##
## Scaled residuals:
##     Min      1Q  Median      3Q     Max
## -5.0210 -0.4288  0.1448  0.5174  2.9717
##
## Random effects:
##  Groups    Name        Variance Std.Dev.
##  SubjectID (Intercept) 2.387    1.545
##  Residual              1.598    1.264
## Number of obs: 1422, groups:  SubjectID, 79
##
## Fixed effects:
##                      Estimate Std. Error         df t value Pr(>|t|)
## (Intercept)         3.224e+00  1.898e+00  7.881e+01   1.699   0.0933 .
## WPstepsRC          -2.294e-01  1.236e-01  1.347e+03  -1.856   0.0636 .
## Age                 3.613e-02  2.415e-02  7.830e+01   1.496   0.1387
## Gender             -4.678e-01  4.047e-01  7.853e+01  -1.156   0.2512
## PMstepsRC          -8.770e-02  8.216e-02  7.776e+01  -1.067   0.2891
## Condition          -4.943e-01  3.689e-01  7.842e+01  -1.340   0.1842
## EdYrs               4.948e-02  7.051e-02  7.785e+01   0.702   0.4849
## T1sf_GeneralHealth  2.652e-02  1.066e-02  8.098e+01   2.487   0.0150 *
## WPstepsRC:Age       4.614e-03  1.963e-03  1.346e+03   2.351   0.0189 *
## ---
## Signif. codes:  0 '***' 0.001 '**' 0.01 '*' 0.05 '.' 0.1 ' ' 1
##
## Correlation of Fixed Effects:
##             (Intr) WPstRC Age    Gender PMstRC Condtn EdYrs  T1s_GH
## WPstepsRC   -0.011
## Age         -0.583  0.016
## Gender      -0.368 -0.005 -0.030
## PMstepsRC   -0.272 -0.004  0.030  0.261
## Condition   -0.172 -0.004  0.048  0.114  0.141
## EdYrs       -0.437  0.000 -0.241  0.028  0.026  0.072
## T1sf_GnrlHl -0.136  0.002 -0.159 -0.134 -0.214 -0.212 -0.021
## WPstpsRC:Ag  0.011 -0.993 -0.017  0.005  0.003  0.005  0.000 -0.002
```

```
m1y<-lmer(mood~WPstepsRC + PMstepsRC + Gender  + Condition  + EdYrs  + T1sf_GeneralHealt
h  +  (1|SubjectID), mystepmateY, REML=FALSE)
summary(m1y)
```

```
## Linear mixed model fit by maximum likelihood . t-tests use
##   Satterthwaite's method [lmerModLmerTest]
## Formula:
## mood ~ WPstepsRC + PMstepsRC + Gender + Condition + EdYrs + T1sf_GeneralHealth +
##     (1 | SubjectID)
##    Data: mystepmateY
##
##      AIC      BIC   logLik deviance df.resid
##   2260.1   2300.3  -1121.1   2242.1      630
##
## Scaled residuals:
##     Min      1Q  Median      3Q     Max
## -4.5241 -0.4142  0.1438  0.5022  2.9609
##
## Random effects:
##  Groups    Name        Variance Std.Dev.
##  SubjectID (Intercept) 2.332    1.527
##  Residual              1.633    1.278
## Number of obs: 639, groups:  SubjectID, 37
##
## Fixed effects:
##                     Estimate Std. Error        df t value Pr(>|t|)
## (Intercept)          4.21129    2.04716  36.29716   2.057   0.0469 *
## WPstepsRC            0.04234    0.02122 604.37666   1.995   0.0464 *
## PMstepsRC           -0.07692    0.10760  35.74772  -0.715   0.4794
## Gender              -0.44713    0.58835  36.23229  -0.760   0.4522
## Condition           -0.22010    0.55355  36.58263  -0.398   0.6932
## EdYrs                0.03813    0.09591  36.07641   0.398   0.6933
## T1sf_GeneralHealth   0.03959    0.01533  37.82403   2.583   0.0138 *
## ---
## Signif. codes:  0 '***' 0.001 '**' 0.01 '*' 0.05 '.' 0.1 ' ' 1
##
## Correlation of Fixed Effects:
##             (Intr) WPstRC PMstRC Gender Condtn EdYrs
## WPstepsRC    0.005
## PMstepsRC   -0.346 -0.004
## Gender      -0.487 -0.007  0.386
## Condition   -0.081  0.000  0.156  0.042
## EdYrs       -0.687 -0.002  0.011 -0.081  0.061
## T1sf_GnrlHl -0.276 -0.004 -0.283 -0.075 -0.348 -0.069
```

```
m1o<-lmer(mood~WPstepsRC + PMstepsRC + Gender  + Condition  + EdYrs  + T1sf_GeneralHealt
h  +  (1|SubjectID), mystepmateO, REML=FALSE)
summary(m1o)
```

```
## Linear mixed model fit by maximum likelihood . t-tests use
##   Satterthwaite's method [lmerModLmerTest]
## Formula:
## mood ~ WPstepsRC + PMstepsRC + Gender + Condition + EdYrs + T1sf_GeneralHealth +
##     (1 | SubjectID)
##    Data: mystepmateO
##
##      AIC      BIC   logLik deviance df.resid
##   2730.3   2772.3  -1356.2   2712.3      774
##
## Scaled residuals:
##     Min      1Q  Median      3Q     Max
## -5.0574 -0.4704  0.1530  0.5405  2.7969
##
## Random effects:
##  Groups    Name        Variance Std.Dev.
##  SubjectID (Intercept) 2.126    1.458
##  Residual              1.578    1.256
## Number of obs: 783, groups:  SubjectID, 42
##
## Fixed effects:
##                      Estimate Std. Error         df t value Pr(>|t|)
## (Intercept)          7.552610   2.330340  42.829109   3.241 0.002308 **
## WPstepsRC            0.075974   0.021269 743.772746   3.572 0.000377 ***
## PMstepsRC           -0.064032   0.131535  42.125235  -0.487 0.628922
## Gender              -0.479290   0.545516  42.425762  -0.879 0.384567
## Condition           -0.854852   0.474203  42.166020  -1.803 0.078584 .
## EdYrs                0.042505   0.097526  41.804155   0.436 0.665202
## T1sf_GeneralHealth   0.005284   0.014741  43.446145   0.358 0.721754
## ---
## Signif. codes:  0 '***' 0.001 '**' 0.01 '*' 0.05 '.' 0.1 ' ' 1
##
## Correlation of Fixed Effects:
##             (Intr) WPstRC PMstRC Gender Condtn EdYrs
## WPstepsRC   -0.002
## PMstepsRC   -0.147 -0.009
## Gender      -0.470  0.006  0.028
## Condition   -0.243  0.011  0.121  0.159
## EdYrs       -0.788 -0.001 -0.076  0.153  0.111
## T1sf_GnrlHl -0.379 -0.003 -0.163 -0.154 -0.079  0.042
```

```
m2<-lmer(energy~WPstepsRC + PMstepsRC + Gender  + Age  + Condition  + EdYrs  + T1sf_Gene
ralHealth  +  (1|SubjectID), stepmate, REML=FALSE)
summary(m2)
```

```
## Linear mixed model fit by maximum likelihood . t-tests use
##   Satterthwaite's method [lmerModLmerTest]
## Formula:
## energy ~ WPstepsRC + PMstepsRC + Gender + Age + Condition + EdYrs +
##     T1sf_GeneralHealth + (1 | SubjectID)
##    Data: stepmate
##
##      AIC      BIC   logLik deviance df.resid
##   5303.3   5355.8  -2641.6   5283.3     1412
##
## Scaled residuals:
##     Min      1Q  Median      3Q     Max
## -3.9163 -0.4907  0.1105  0.5995  3.0671
##
## Random effects:
##  Groups    Name        Variance Std.Dev.
##  SubjectID (Intercept) 1.883    1.372
##  Residual              2.064    1.437
## Number of obs: 1422, groups:  SubjectID, 79
##
## Fixed effects:
##                     Estimate Std. Error         df t value Pr(>|t|)
## (Intercept)         2.932e+00  1.711e+00  7.916e+01   1.714 0.090439 .
## WPstepsRC           1.059e-01  1.702e-02  1.351e+03   6.224 6.44e-10 ***
## PMstepsRC          -1.153e-02  7.391e-02  7.756e+01  -0.156 0.876407
## Gender             -6.215e-01  3.645e-01  7.864e+01  -1.705 0.092190 .
## Age                -1.557e-03  2.174e-02  7.833e+01  -0.072 0.943115
## Condition          -1.294e-01  3.323e-01  7.848e+01  -0.389 0.698070
## EdYrs               9.280e-02  6.345e-02  7.777e+01   1.463 0.147594
## T1sf_GeneralHealth  3.610e-02  9.650e-03  8.228e+01   3.742 0.000337 ***
## ---
## Signif. codes:  0 '***' 0.001 '**' 0.01 '*' 0.05 '.' 0.1 ' ' 1
##
## Correlation of Fixed Effects:
##             (Intr) WPstRC PMstRC Gender Age    Condtn EdYrs
## WPstepsRC    0.004
## PMstepsRC   -0.271 -0.005
## Gender      -0.367 -0.002  0.260
## Age         -0.583 -0.002  0.029 -0.031
## Condition   -0.167  0.006  0.141  0.112  0.046
## EdYrs       -0.438 -0.003  0.025  0.026 -0.240  0.070
## T1sf_GnrlHl -0.141 -0.005 -0.213 -0.131 -0.159 -0.213 -0.018
```

```
m2i<-lmer(energy~WPstepsRC*Gender + PMstepsRC + Age  + Condition  + EdYrs  + T1sf_Genera
lHealth  +  (1|SubjectID), stepmate, REML=FALSE)
summary(m2i)
```

```
## Linear mixed model fit by maximum likelihood . t-tests use
##   Satterthwaite's method [lmerModLmerTest]
## Formula:
## energy ~ WPstepsRC * Gender + PMstepsRC + Age + Condition + EdYrs +
##     T1sf_GeneralHealth + (1 | SubjectID)
##    Data: stepmate
##
##      AIC      BIC   logLik deviance df.resid
##   5286.9   5344.7  -2632.4   5264.9     1411
##
## Scaled residuals:
##     Min      1Q  Median      3Q     Max
## -4.0503 -0.4959  0.1140  0.5829  3.1474
##
## Random effects:
##  Groups    Name        Variance Std.Dev.
##  SubjectID (Intercept) 1.874    1.369
##  Residual              2.036    1.427
## Number of obs: 1422, groups:  SubjectID, 79
##
## Fixed effects:
##                      Estimate Std. Error         df t value Pr(>|t|)
## (Intercept)         3.020e+00  1.706e+00  7.921e+01   1.770 0.080601 .
## WPstepsRC          -1.410e-01  5.982e-02  1.349e+03  -2.357 0.018578 *
## Gender             -6.660e-01  3.637e-01  7.880e+01  -1.831 0.070880 .
## PMstepsRC          -1.472e-02  7.373e-02  7.761e+01  -0.200 0.842325
## Age                -9.322e-04  2.169e-02  7.837e+01  -0.043 0.965824
## Condition          -1.147e-01  3.315e-01  7.854e+01  -0.346 0.730276
## EdYrs               9.064e-02  6.329e-02  7.782e+01   1.432 0.156060
## T1sf_GeneralHealth  3.593e-02  9.624e-03  8.229e+01   3.734 0.000347 ***
## WPstepsRC:Gender    1.511e-01  3.511e-02  1.350e+03   4.303 1.81e-05 ***
## ---
## Signif. codes:  0 '***' 0.001 '**' 0.01 '*' 0.05 '.' 0.1 ' ' 1
##
## Correlation of Fixed Effects:
##             (Intr) WPstRC Gender PMstRC Age    Condtn EdYrs  T1s_GH
## WPstepsRC   -0.010
## Gender      -0.367  0.027
## PMstepsRC   -0.271  0.008  0.261
## Age         -0.583 -0.007 -0.031  0.029
## Condition   -0.167 -0.008  0.112  0.141  0.047
## EdYrs       -0.438  0.007  0.026  0.026 -0.240  0.070
## T1sf_GnrlHl -0.141  0.003 -0.131 -0.213 -0.159 -0.213 -0.018
## WPstpsRC:Gn  0.012 -0.959 -0.028 -0.010  0.007  0.010 -0.008 -0.004
```

```
m2f<-lmer(energy~WPstepsRC + PMstepsRC  + Age  + Condition  + EdYrs  + T1sf_GeneralHealt
h  +  (1|SubjectID), mystepmateF, REML=FALSE)
summary(m2f)
```

```
## Linear mixed model fit by maximum likelihood . t-tests use
##   Satterthwaite's method [lmerModLmerTest]
## Formula:
## energy ~ WPstepsRC + PMstepsRC + Age + Condition + EdYrs + T1sf_GeneralHealth +
##     (1 | SubjectID)
##    Data: mystepmateF
##
##      AIC      BIC   logLik deviance df.resid
##   3798.1   3842.1  -1890.1   3780.1      976
##
## Scaled residuals:
##     Min      1Q  Median      3Q     Max
## -3.7988 -0.5306  0.1131  0.6449  2.9084
##
## Random effects:
##  Groups    Name        Variance Std.Dev.
##  SubjectID (Intercept) 1.764    1.328
##  Residual              2.354    1.534
## Number of obs: 985, groups:  SubjectID, 55
##
## Fixed effects:
##                     Estimate Std. Error        df t value Pr(>|t|)
## (Intercept)          2.56895    1.82899  54.95879   1.405 0.165776
## WPstepsRC            0.16071    0.02282 936.15063   7.043 3.65e-12 ***
## PMstepsRC            0.12764    0.10935  53.04284   1.167 0.248303
## Age                 -0.02513    0.02501  53.28748  -1.005 0.319556
## Condition           -0.25338    0.38905  53.37880  -0.651 0.517651
## EdYrs                0.07905    0.07315  53.17060   1.081 0.284760
## T1sf_GeneralHealth   0.03968    0.01097  56.69091   3.618 0.000633 ***
## ---
## Signif. codes:  0 '***' 0.001 '**' 0.01 '*' 0.05 '.' 0.1 ' ' 1
##
## Correlation of Fixed Effects:
##             (Intr) WPstRC PMstRC Age    Condtn EdYrs
## WPstepsRC    0.002
## PMstepsRC   -0.021 -0.014
## Age         -0.664  0.004  0.019
## Condition   -0.132  0.015  0.159  0.100
## EdYrs       -0.494 -0.006 -0.225 -0.169 -0.002
## T1sf_GnrlHl -0.178 -0.007 -0.308 -0.229 -0.237  0.090
```

```
m2m<-lmer(energy~WPstepsRC + PMstepsRC  + Age  + Condition  + EdYrs  + T1sf_GeneralHealt
h  +  (1|SubjectID), mystepmateM, REML=FALSE)
summary(m2m)
```

```
## Linear mixed model fit by maximum likelihood . t-tests use
##   Satterthwaite's method [lmerModLmerTest]
## Formula:
## energy ~ WPstepsRC + PMstepsRC + Age + Condition + EdYrs + T1sf_GeneralHealth +
##     (1 | SubjectID)
##    Data: mystepmateM
##
##      AIC      BIC   logLik deviance df.resid
##   1450.4   1487.2   -716.2   1432.4      428
##
## Scaled residuals:
##     Min      1Q  Median      3Q     Max
## -3.6387 -0.4295  0.0674  0.5058  2.5919
##
## Random effects:
##  Groups    Name        Variance Std.Dev.
##  SubjectID (Intercept) 1.421    1.192
##  Residual              1.325    1.151
## Number of obs: 437, groups:  SubjectID, 24
##
## Fixed effects:
##                     Estimate Std. Error         df t value Pr(>|t|)
## (Intercept)         2.698908   2.787347  24.169961   0.968    0.342
## WPstepsRC           0.009205   0.022557 414.791808   0.408    0.683
## PMstepsRC          -0.163929   0.096179  23.758826  -1.704    0.101
## Age                 0.056897   0.038562  24.708217   1.475    0.153
## Condition           0.107022   0.538381  24.779711   0.199    0.844
## EdYrs              -0.039607   0.128008  24.400971  -0.309    0.760
## T1sf_GeneralHealth   0.020318   0.018704  25.757900   1.086    0.287
##
## Correlation of Fixed Effects:
##             (Intr) WPstRC PMstRC Age    Condtn EdYrs
## WPstepsRC    0.000
## PMstepsRC   -0.398  0.010
## Age         -0.546 -0.014 -0.075
## Condition   -0.105 -0.004  0.187 -0.147
## EdYrs       -0.370  0.013  0.409 -0.454  0.250
## T1sf_GnrlHl -0.345 -0.003 -0.140  0.149 -0.234 -0.239
```

```
m2.age<-lmer(energy~WPstepsRC*Age  + Gender + PMstepsRC +  Condition  + EdYrs  + T1sf_Ge
neralHealth  +  (1|SubjectID), stepmate, REML=FALSE)
summary(m2.age)
```

```
## Linear mixed model fit by maximum likelihood . t-tests use
##   Satterthwaite's method [lmerModLmerTest]
## Formula:
## energy ~ WPstepsRC * Age + Gender + PMstepsRC + Condition + EdYrs +
##     T1sf_GeneralHealth + (1 | SubjectID)
##    Data: stepmate
##
##      AIC      BIC   logLik deviance df.resid
##   5298.7   5356.6  -2638.4   5276.7     1411
##
## Scaled residuals:
##     Min      1Q  Median      3Q     Max
## -4.0734 -0.4973  0.1171  0.5948  3.0958
##
## Random effects:
##  Groups    Name        Variance Std.Dev.
##  SubjectID (Intercept) 1.883    1.372
##  Residual              2.054    1.433
## Number of obs: 1422, groups:  SubjectID, 79
##
## Fixed effects:
##                      Estimate Std. Error         df t value Pr(>|t|)
## (Intercept)         2.994e+00  1.711e+00  7.915e+01   1.750 0.083945 .
## WPstepsRC          -2.503e-01  1.401e-01  1.349e+03  -1.787 0.074230 .
## Age                -2.741e-03  2.175e-02  7.836e+01  -0.126 0.900010
## Gender             -6.159e-01  3.645e-01  7.861e+01  -1.690 0.095016 .
## PMstepsRC          -1.077e-02  7.390e-02  7.753e+01  -0.146 0.884526
## Condition          -1.242e-01  3.322e-01  7.845e+01  -0.374 0.709605
## EdYrs               9.280e-02  6.344e-02  7.774e+01   1.463 0.147554
## T1sf_GeneralHealth  3.603e-02  9.647e-03  8.223e+01   3.735 0.000345 ***
## WPstepsRC:Age       5.698e-03  2.224e-03  1.349e+03   2.562 0.010521 *
## ---
## Signif. codes:  0 '***' 0.001 '**' 0.01 '*' 0.05 '.' 0.1 ' ' 1
##
## Correlation of Fixed Effects:
##             (Intr) WPstRC Age    Gender PMstRC Condtn EdYrs  T1s_GH
## WPstepsRC   -0.013
## Age         -0.583  0.021
## Gender      -0.367 -0.006 -0.031
## PMstepsRC   -0.271 -0.005  0.029  0.260
## Condition   -0.167 -0.005  0.046  0.112  0.141
## EdYrs       -0.437  0.000 -0.240  0.026  0.025  0.070
## T1sf_GnrlHl -0.141  0.002 -0.159 -0.131 -0.213 -0.213 -0.018
## WPstpsRC:Ag  0.014 -0.993 -0.021  0.006  0.004  0.006  0.000 -0.003
```

```
m2y<-lmer(energy~WPstepsRC + PMstepsRC + Gender  + Condition  + EdYrs  + T1sf_GeneralHea
lth  +  (1|SubjectID), mystepmateY, REML=FALSE)
summary(m2y)
```

```
## Linear mixed model fit by maximum likelihood . t-tests use
##   Satterthwaite's method [lmerModLmerTest]
## Formula: energy ~ WPstepsRC + PMstepsRC + Gender + Condition + EdYrs +
##     T1sf_GeneralHealth + (1 | SubjectID)
##    Data: mystepmateY
##
##      AIC      BIC   logLik deviance df.resid
##   2294.3   2334.4  -1138.1   2276.3      630
##
## Scaled residuals:
##     Min      1Q  Median      3Q     Max
## -4.1773 -0.4778  0.1380  0.5732  2.7501
##
## Random effects:
##  Groups    Name        Variance Std.Dev.
##  SubjectID (Intercept) 2.470    1.572
##  Residual              1.723    1.313
## Number of obs: 639, groups:  SubjectID, 37
##
## Fixed effects:
##                     Estimate Std. Error        df t value Pr(>|t|)
## (Intercept)          2.68021    2.10644  36.43290   1.272   0.2113
## WPstepsRC            0.05057    0.02179 604.49502   2.320   0.0207 *
## PMstepsRC           -0.03332    0.11072  35.88364  -0.301   0.7652
## Gender              -0.32579    0.60539  36.36827  -0.538   0.5938
## Condition            0.05073    0.56958  36.71864   0.089   0.9295
## EdYrs                0.10898    0.09868  36.21221   1.104   0.2767
## T1sf_GeneralHealth   0.02720    0.01577  37.95956   1.724   0.0928 .
## ---
## Signif. codes:  0 '***' 0.001 '**' 0.01 '*' 0.05 '.' 0.1 ' ' 1
##
## Correlation of Fixed Effects:
##             (Intr) WPstRC PMstRC Gender Condtn EdYrs
## WPstepsRC    0.005
## PMstepsRC   -0.346 -0.004
## Gender      -0.487 -0.007  0.386
## Condition   -0.081  0.000  0.156  0.042
## EdYrs       -0.687 -0.002  0.011 -0.081  0.062
## T1sf_GnrlHl -0.276 -0.004 -0.283 -0.075 -0.348 -0.069
```

```
m2o<-lmer(energy~WPstepsRC + PMstepsRC + Gender  + Condition  + EdYrs  + T1sf_GeneralHea
lth  +  (1|SubjectID), mystepmateO, REML=FALSE)
summary(m2o)
```

```
## Linear mixed model fit by maximum likelihood . t-tests use
##   Satterthwaite's method [lmerModLmerTest]
## Formula: energy ~ WPstepsRC + PMstepsRC + Gender + Condition + EdYrs +
##     T1sf_GeneralHealth + (1 | SubjectID)
##    Data: mystepmateO
##
##      AIC      BIC   logLik deviance df.resid
##   2992.4   3034.4  -1487.2   2974.4      774
##
## Scaled residuals:
##     Min      1Q  Median      3Q     Max
## -3.8158 -0.5450  0.0854  0.6170  2.9254
##
## Random effects:
##  Groups    Name        Variance Std.Dev.
##  SubjectID (Intercept) 1.217    1.103
##  Residual              2.310    1.520
## Number of obs: 783, groups:  SubjectID, 42
##
## Fixed effects:
##                     Estimate Std. Error        df t value Pr(>|t|)
## (Intercept)          3.29865    1.83298  43.64934   1.800 0.078837 .
## WPstepsRC            0.16326    0.02571 747.32480   6.351 3.72e-10 ***
## PMstepsRC            0.10434    0.10293  42.25701   1.014 0.316494
## Gender              -1.10727    0.42780  42.82939  -2.588 0.013120 *
## Condition           -0.22683    0.37117  42.32532  -0.611 0.544393
## EdYrs                0.05052    0.07617  41.84957   0.663 0.510816
## T1sf_GeneralHealth   0.04497    0.01163  44.35813   3.865 0.000359 ***
## ---
## Signif. codes:  0 '***' 0.001 '**' 0.01 '*' 0.05 '.' 0.1 ' ' 1
##
## Correlation of Fixed Effects:
##             (Intr) WPstRC PMstRC Gender Condtn EdYrs
## WPstepsRC   -0.002
## PMstepsRC   -0.143 -0.014
## Gender      -0.476  0.008  0.026
## Condition   -0.243  0.017  0.120  0.158
## EdYrs       -0.785 -0.002 -0.077  0.157  0.112
## T1sf_GnrlHl -0.390 -0.005 -0.166 -0.139 -0.075  0.048
```

Next Day mood/energy

```
##lag analyses, predicting next day mood/energy

m3<-lmer(mood~WPlagstepsRC + lagMood + PMstepsRC + Gender  + Age  + Condition  + EdYrs
 + T1sf_GeneralHealth  +  (1|SubjectID), stepmate, REML=FALSE)
summary(m3)
```

```
## Linear mixed model fit by maximum likelihood . t-tests use
##   Satterthwaite's method [lmerModLmerTest]
## Formula:
## mood ~ WPlagstepsRC + lagMood + PMstepsRC + Gender + Age + Condition +
##     EdYrs + T1sf_GeneralHealth + (1 | SubjectID)
##    Data: stepmate
##
##      AIC      BIC   logLik deviance df.resid
##   4042.8   4098.7  -2010.4   4020.8     1176
##
## Scaled residuals:
##     Min      1Q  Median      3Q     Max
## -4.7316 -0.4285  0.1549  0.5038  3.0535
##
## Random effects:
##  Groups    Name        Variance Std.Dev.
##  SubjectID (Intercept) 1.198    1.095
##  Residual              1.479    1.216
## Number of obs: 1187, groups:  SubjectID, 75
##
## Fixed effects:
##                     Estimate Std. Error         df t value Pr(>|t|)
## (Intercept)         1.888e+00  1.417e+00  5.670e+01   1.332   0.1882
## WPlagstepsRC        4.129e-02  1.605e-02  1.113e+03   2.572   0.0102 *
## lagMood             3.041e-01  2.761e-02  1.127e+03  11.013   <2e-16 ***
## PMstepsRC          -5.347e-02  6.068e-02  5.493e+01  -0.881   0.3821
## Gender             -4.175e-01  3.010e-01  5.561e+01  -1.387   0.1710
## Age                 3.516e-02  1.788e-02  5.641e+01   1.967   0.0541 .
## Condition          -4.449e-01  2.749e-01  5.547e+01  -1.619   0.1112
## EdYrs               2.898e-02  5.195e-02  5.592e+01   0.558   0.5792
## T1sf_GeneralHealth  1.727e-02  8.187e-03  5.889e+01   2.110   0.0391 *
## ---
## Signif. codes:  0 '***' 0.001 '**' 0.01 '*' 0.05 '.' 0.1 ' ' 1
##
## Correlation of Fixed Effects:
##             (Intr) WPlgRC lagMod PMstRC Gender Age    Condtn EdYrs
## WPlagstpsRC  0.016
## lagMood     -0.062 -0.104
## PMstepsRC   -0.268 -0.020  0.037
## Gender      -0.369 -0.019  0.048  0.267
## Age         -0.568  0.014 -0.056  0.017 -0.043
## Condition   -0.163  0.002  0.045  0.137  0.110  0.039
## EdYrs       -0.440 -0.010 -0.034  0.027  0.016 -0.228  0.070
## T1sf_GnrlHl -0.166 -0.001 -0.082 -0.206 -0.094 -0.162 -0.201  0.002
```

```
m3i<-lmer(mood~WPlagstepsRC*Gender  + lagMood + PMstepsRC + Age  + Condition  + EdYrs  +
T1sf_GeneralHealth  +  (1|SubjectID), stepmate, REML=FALSE)
summary(m3i)
```

```
## Linear mixed model fit by maximum likelihood . t-tests use
##   Satterthwaite's method [lmerModLmerTest]
## Formula:
## mood ~ WPlagstepsRC * Gender + lagMood + PMstepsRC + Age + Condition +
##     EdYrs + T1sf_GeneralHealth + (1 | SubjectID)
##    Data: stepmate
##
##      AIC      BIC   logLik deviance df.resid
##   4039.5   4100.4  -2007.7   4015.5     1175
##
## Scaled residuals:
##     Min      1Q  Median      3Q     Max
## -4.7147 -0.4416  0.1541  0.5139  3.0757
##
## Random effects:
##  Groups    Name        Variance Std.Dev.
##  SubjectID (Intercept) 1.219    1.104
##  Residual              1.471    1.213
## Number of obs: 1187, groups:  SubjectID, 75
##
## Fixed effects:
##                       Estimate Std. Error         df t value Pr(>|t|)
## (Intercept)          1.985e+00  1.429e+00  5.705e+01   1.390   0.1700
## WPlagstepsRC        -8.470e-02  5.674e-02  1.104e+03  -1.493   0.1358
## Gender              -4.440e-01  3.036e-01  5.604e+01  -1.463   0.1492
## lagMood              2.980e-01  2.763e-02  1.131e+03  10.786   <2e-16 ***
## PMstepsRC           -5.526e-02  6.115e-02  5.524e+01  -0.904   0.3701
## Age                  3.550e-02  1.802e-02  5.670e+01   1.970   0.0537 .
## Condition           -4.371e-01  2.770e-01  5.577e+01  -1.578   0.1203
## EdYrs                2.689e-02  5.236e-02  5.622e+01   0.514   0.6096
## T1sf_GeneralHealth   1.729e-02  8.248e-03  5.915e+01   2.097   0.0403 *
## WPlagstepsRC:Gender  7.704e-02  3.326e-02  1.107e+03   2.316   0.0207 *
## ---
## Signif. codes:  0 '***' 0.001 '**' 0.01 '*' 0.05 '.' 0.1 ' ' 1
##
## Correlation of Fixed Effects:
##             (Intr) WPlgRC Gender lagMod PMstRC Age    Condtn EdYrs  T1s_GH
## WPlagstpsRC -0.023
## Gender      -0.370  0.030
## lagMood     -0.063  0.037  0.050
## PMstepsRC   -0.268  0.005  0.267  0.037
## Age         -0.568 -0.003 -0.043 -0.056  0.017
## Condition   -0.163 -0.012  0.110  0.044  0.137  0.039
## EdYrs       -0.440  0.015  0.017 -0.032  0.027 -0.228  0.070
## T1sf_GnrlHl -0.166  0.002 -0.094 -0.081 -0.206 -0.162 -0.201  0.002
## WPlgstpRC:G  0.028 -0.959 -0.036 -0.069 -0.011  0.008  0.013 -0.019 -0.003
```

```
m3f<-lmer(mood~WPlagstepsRC + lagMood + PMstepsRC  + Age  + Condition  + EdYrs  + T1sf_G
eneralHealth  +  (1|SubjectID), mystepmateF, REML=FALSE)
summary(m3f)
```

```
## Linear mixed model fit by maximum likelihood . t-tests use
##   Satterthwaite's method [lmerModLmerTest]
## Formula: mood ~ WPlagstepsRC + lagMood + PMstepsRC + Age + Condition +
##     EdYrs + T1sf_GeneralHealth + (1 | SubjectID)
##    Data: mystepmateF
##
##      AIC      BIC   logLik deviance df.resid
##   2908.3   2955.2  -1444.1   2888.3      796
##
## Scaled residuals:
##     Min      1Q  Median      3Q     Max
## -4.2606 -0.4329  0.1463  0.5577  2.7820
##
## Random effects:
##  Groups    Name        Variance Std.Dev.
##  SubjectID (Intercept) 1.320    1.149
##  Residual              1.807    1.344
## Number of obs: 806, groups:  SubjectID, 52
##
## Fixed effects:
##                     Estimate Std. Error        df t value Pr(>|t|)
## (Intercept)          1.21186    1.64728  38.08552   0.736  0.46644
## WPlagstepsRC         0.06782    0.02225 757.49763   3.048  0.00239 **
## lagMood              0.30848    0.03344 758.19501   9.224  < 2e-16 ***
## PMstepsRC            0.01605    0.09841  35.84593   0.163  0.87140
## Age                  0.02126    0.02220  37.39034   0.958  0.34433
## Condition           -0.35468    0.34819  37.20983  -1.019  0.31496
## EdYrs                0.03329    0.06514  37.47258   0.511  0.61229
## T1sf_GeneralHealth   0.02089    0.01009  40.32114   2.070  0.04494 *
## ---
## Signif. codes:  0 '***' 0.001 '**' 0.01 '*' 0.05 '.' 0.1 ' ' 1
##
## Correlation of Fixed Effects:
##             (Intr) WPlgRC lagMod PMstRC Age    Condtn EdYrs
## WPlagstpsRC  0.016
## lagMood     -0.042 -0.135
## PMstepsRC    0.003 -0.021 -0.002
## Age         -0.653  0.015 -0.031 -0.005
## Condition   -0.125  0.015  0.037  0.156  0.083
## EdYrs       -0.512 -0.019 -0.037 -0.225 -0.153 -0.009
## T1sf_GnrlHl -0.204  0.000 -0.103 -0.310 -0.221 -0.204  0.124
```

```
m3m<-lmer(mood~WPlagstepsRC + lagMood + PMstepsRC  + Age  + Condition  + EdYrs  + T1sf_G
eneralHealth  +  (1|SubjectID), mystepmateM, REML=FALSE)
summary(m3m)
```

```
## Linear mixed model fit by maximum likelihood . t-tests use
##   Satterthwaite's method [lmerModLmerTest]
## Formula: mood ~ WPlagstepsRC + lagMood + PMstepsRC + Age + Condition +
##     EdYrs + T1sf_GeneralHealth + (1 | SubjectID)
##    Data: mystepmateM
##
##      AIC      BIC   logLik deviance df.resid
##   1059.2   1098.6   -519.6   1039.2      371
##
## Scaled residuals:
##     Min      1Q  Median      3Q     Max
## -3.8800 -0.5305  0.2178  0.5557  2.6974
##
## Random effects:
##  Groups    Name        Variance Std.Dev.
##  SubjectID (Intercept) 0.6873   0.829
##  Residual              0.7640   0.874
## Number of obs: 381, groups:  SubjectID, 23
##
## Fixed effects:
##                     Estimate Std. Error         df t value Pr(>|t|)
## (Intercept)          2.927177   1.995013  20.657309   1.467   0.1574
## WPlagstepsRC        -0.007386   0.019141 357.788342  -0.386   0.6998
## lagMood              0.255716   0.049103 374.149425   5.208 3.16e-07 ***
## PMstepsRC           -0.157175   0.068536  19.897437  -2.293   0.0329 *
## Age                  0.075986   0.027874  21.941640   2.726   0.0124 *
## Condition           -0.700283   0.394033  20.221629  -1.777   0.0906 .
## EdYrs               -0.086600   0.091573  19.814571  -0.946   0.3557
## T1sf_GeneralHealth   0.002639   0.014057  20.008135   0.188   0.8530
## ---
## Signif. codes:  0 '***' 0.001 '**' 0.01 '*' 0.05 '.' 0.1 ' ' 1
##
## Correlation of Fixed Effects:
##             (Intr) WPlgRC lagMod PMstRC Age    Condtn EdYrs
## WPlagstpsRC -0.010
## lagMood     -0.127 -0.017
## PMstepsRC   -0.409  0.000  0.142
## Age         -0.531  0.004 -0.125 -0.080
## Condition   -0.086 -0.014  0.070  0.192 -0.168
## EdYrs       -0.345  0.013  0.028  0.397 -0.455  0.286
## T1sf_GnrlHl -0.339 -0.001  0.015 -0.130  0.143 -0.302 -0.276
```

```
m3.age<-lmer(mood~WPlagstepsRC*Age  + Gender  + lagMood + PMstepsRC +  Condition  + EdYr
s  + T1sf_GeneralHealth  +  (1|SubjectID), stepmate, REML=FALSE)
summary(m3.age)
```

```
## Linear mixed model fit by maximum likelihood . t-tests use
##   Satterthwaite's method [lmerModLmerTest]
## Formula:
## mood ~ WPlagstepsRC * Age + Gender + lagMood + PMstepsRC + Condition +
##     EdYrs + T1sf_GeneralHealth + (1 | SubjectID)
##    Data: stepmate
##
##      AIC      BIC   logLik deviance df.resid
##   4044.7   4105.6  -2010.3   4020.7     1175
##
## Scaled residuals:
##     Min      1Q  Median      3Q     Max
## -4.7312 -0.4274  0.1489  0.5072  3.0525
##
## Random effects:
##  Groups    Name        Variance Std.Dev.
##  SubjectID (Intercept) 1.201    1.096
##  Residual              1.479    1.216
## Number of obs: 1187, groups:  SubjectID, 75
##
## Fixed effects:
##                     Estimate Std. Error         df t value Pr(>|t|)
## (Intercept)         1.898e+00  1.419e+00  5.673e+01   1.338   0.1863
## WPlagstepsRC       -5.312e-03  1.276e-01  1.100e+03  -0.042   0.9668
## Age                 3.508e-02  1.790e-02  5.643e+01   1.960   0.0550 .
## Gender             -4.181e-01  3.014e-01  5.561e+01  -1.387   0.1708
## lagMood             3.031e-01  2.769e-02  1.127e+03  10.947   <2e-16 ***
## PMstepsRC          -5.335e-02  6.074e-02  5.493e+01  -0.878   0.3836
## Condition          -4.443e-01  2.752e-01  5.547e+01  -1.614   0.1121
## EdYrs               2.909e-02  5.200e-02  5.591e+01   0.559   0.5781
## T1sf_GeneralHealth  1.727e-02  8.195e-03  5.888e+01   2.107   0.0394 *
## WPlagstepsRC:Age    7.487e-04  2.033e-03  1.099e+03   0.368   0.7127
## ---
## Signif. codes:  0 '***' 0.001 '**' 0.01 '*' 0.05 '.' 0.1 ' ' 1
##
## Correlation of Fixed Effects:
##             (Intr) WPlgRC Age    Gender lagMod PMstRC Condtn EdYrs  T1s_GH
## WPlagstpsRC -0.017
## Age         -0.568  0.015
## Gender      -0.369  0.002 -0.043
## lagMood     -0.063  0.062 -0.055  0.048
## PMstepsRC   -0.268 -0.009  0.017  0.267  0.036
## Condition   -0.163 -0.007  0.039  0.110  0.044  0.137
## EdYrs       -0.440 -0.006 -0.228  0.016 -0.034  0.027  0.070
## T1sf_GnrlHl -0.166  0.005 -0.162 -0.094 -0.081 -0.206 -0.201  0.002
## WPlgstpRC:A  0.019 -0.992 -0.013 -0.005 -0.075  0.006  0.008  0.005 -0.005
```

```
m4<-lmer(energy~WPlagstepsRC + lagEnergy + PMstepsRC + Gender  + Age  + Condition  + EdY
rs  + T1sf_GeneralHealth  +  (1|SubjectID), stepmate, REML=FALSE)
summary(m4)
```

```
## Linear mixed model fit by maximum likelihood . t-tests use
##   Satterthwaite's method [lmerModLmerTest]
## Formula: energy ~ WPlagstepsRC + lagEnergy + PMstepsRC + Gender + Age +
##     Condition + EdYrs + T1sf_GeneralHealth + (1 | SubjectID)
##    Data: stepmate
##
##      AIC      BIC   logLik deviance df.resid
##   4394.8   4450.7  -2186.4   4372.8     1176
##
## Scaled residuals:
##     Min      1Q  Median      3Q     Max
## -4.2300 -0.5059  0.1259  0.5767  3.6469
##
## Random effects:
##  Groups    Name        Variance Std.Dev.
##  SubjectID (Intercept) 1.114    1.056
##  Residual              2.034    1.426
## Number of obs: 1187, groups:  SubjectID, 75
##
## Fixed effects:
##                      Estimate Std. Error         df t value Pr(>|t|)
## (Intercept)         2.007e+00  1.397e+00  6.137e+01   1.436 0.156088
## WPlagstepsRC        6.035e-03  1.891e-02  1.128e+03   0.319 0.749721
## lagEnergy           2.415e-01  2.922e-02  1.143e+03   8.265 3.84e-16 ***
## PMstepsRC           1.465e-02  5.964e-02  5.886e+01   0.246 0.806807
## Gender             -4.692e-01  2.966e-01  6.015e+01  -1.582 0.118960
## Age                -1.495e-03  1.759e-02  6.035e+01  -0.085 0.932547
## Condition          -1.642e-01  2.704e-01  5.973e+01  -0.607 0.545928
## EdYrs               7.404e-02  5.127e-02  6.095e+01   1.444 0.153798
## T1sf_GeneralHealth  2.931e-02  8.144e-03  6.554e+01   3.599 0.000614 ***
## ---
## Signif. codes:  0 '***' 0.001 '**' 0.01 '*' 0.05 '.' 0.1 ' ' 1
##
## Correlation of Fixed Effects:
##             (Intr) WPlgRC lgEnrg PMstRC Gender Age    Condtn EdYrs
## WPlagstpsRC  0.019
## lagEnergy   -0.057 -0.154
## PMstepsRC   -0.268 -0.019  0.005
## Gender      -0.368 -0.025  0.060  0.266
## Age         -0.573  0.009  0.005  0.020 -0.039
## Condition   -0.153  0.007  0.009  0.135  0.107  0.036
## EdYrs       -0.439 -0.006 -0.064  0.029  0.011 -0.230  0.068
## T1sf_GnrlHl -0.165  0.008 -0.132 -0.199 -0.098 -0.169 -0.198  0.011
```

```
m4i<-lmer(energy~WPlagstepsRC*Gender  + lagEnergy + PMstepsRC + Age  + Condition  + EdYr
s  + T1sf_GeneralHealth  +  (1|SubjectID), stepmate, REML=FALSE)
summary(m4i)
```

```
## Linear mixed model fit by maximum likelihood . t-tests use
##   Satterthwaite's method [lmerModLmerTest]
## Formula: energy ~ WPlagstepsRC * Gender + lagEnergy + PMstepsRC + Age +
##     Condition + EdYrs + T1sf_GeneralHealth + (1 | SubjectID)
##    Data: stepmate
##
##      AIC      BIC   logLik deviance df.resid
##   4396.8   4457.7  -2186.4   4372.8     1175
##
## Scaled residuals:
##     Min      1Q  Median      3Q     Max
## -4.2329 -0.5034  0.1226  0.5760  3.6501
##
## Random effects:
##  Groups    Name        Variance Std.Dev.
##  SubjectID (Intercept) 1.111    1.054
##  Residual              2.034    1.426
## Number of obs: 1187, groups:  SubjectID, 75
##
## Fixed effects:
##                       Estimate Std. Error         df t value Pr(>|t|)
## (Intercept)          1.991e+00  1.397e+00  6.134e+01   1.426 0.158984
## WPlagstepsRC         2.528e-02  6.671e-02  1.116e+03   0.379 0.704774
## Gender              -4.649e-01  2.966e-01  6.025e+01  -1.568 0.122216
## lagEnergy            2.426e-01  2.938e-02  1.141e+03   8.259 4.03e-16 ***
## PMstepsRC            1.485e-02  5.957e-02  5.873e+01   0.249 0.803978
## Age                 -1.507e-03  1.757e-02  6.021e+01  -0.086 0.931922
## Condition           -1.657e-01  2.701e-01  5.962e+01  -0.614 0.541770
## EdYrs                7.429e-02  5.121e-02  6.083e+01   1.451 0.152028
## T1sf_GeneralHealth   2.929e-02  8.135e-03  6.538e+01   3.601 0.000611 ***
## WPlagstepsRC:Gender -1.180e-02  3.918e-02  1.120e+03  -0.301 0.763395
## ---
## Signif. codes:  0 '***' 0.001 '**' 0.01 '*' 0.05 '.' 0.1 ' ' 1
##
## Correlation of Fixed Effects:
##             (Intr) WPlgRC Gender lgEnrg PMstRC Age    Condtn EdYrs  T1s_GH
## WPlagstpsRC -0.027
## Gender      -0.369  0.037
## lagEnergy   -0.060  0.056  0.065
## PMstepsRC   -0.269  0.005  0.266  0.006
## Age         -0.572 -0.001 -0.039  0.005  0.020
## Condition   -0.152 -0.016  0.106  0.007  0.135  0.036
## EdYrs       -0.439  0.015  0.012 -0.062  0.029 -0.230  0.067
## T1sf_GnrlHl -0.165  0.000 -0.098 -0.131 -0.199 -0.169 -0.198  0.011
## WPlgstpRC:G  0.034 -0.959 -0.046 -0.104 -0.011  0.004  0.019 -0.018  0.003
```

```
m4.age<-lmer(energy~WPlagstepsRC*Age  + Gender  + lagEnergy + PMstepsRC + Condition  + E
dYrs  + T1sf_GeneralHealth  +  (1|SubjectID), stepmate, REML=FALSE)
summary(m4.age)
```

```
## Linear mixed model fit by maximum likelihood . t-tests use
##   Satterthwaite's method [lmerModLmerTest]
## Formula: energy ~ WPlagstepsRC * Age + Gender + lagEnergy + PMstepsRC +
##     Condition + EdYrs + T1sf_GeneralHealth + (1 | SubjectID)
##    Data: stepmate
##
##      AIC      BIC   logLik deviance df.resid
##   4396.3   4457.2  -2186.1   4372.3     1175
##
## Scaled residuals:
##     Min      1Q  Median      3Q     Max
## -4.2272 -0.4945  0.1199  0.5771  3.6417
##
## Random effects:
##  Groups    Name        Variance Std.Dev.
##  SubjectID (Intercept) 1.119    1.058
##  Residual              2.032    1.426
## Number of obs: 1187, groups:  SubjectID, 75
##
## Fixed effects:
##                      Estimate Std. Error         df t value Pr(>|t|)
## (Intercept)         2.031e+00  1.400e+00  6.142e+01   1.450  0.15214
## WPlagstepsRC       -1.051e-01  1.493e-01  1.110e+03  -0.704  0.48172
## Age                -1.808e-03  1.763e-02  6.043e+01  -0.103  0.91869
## Gender             -4.705e-01  2.972e-01  6.015e+01  -1.583  0.11864
## lagEnergy           2.397e-01  2.928e-02  1.143e+03   8.187 7.11e-16 ***
## PMstepsRC           1.509e-02  5.976e-02  5.888e+01   0.252  0.80154
## Condition          -1.616e-01  2.709e-01  5.975e+01  -0.596  0.55316
## EdYrs               7.437e-02  5.137e-02  6.095e+01   1.448  0.15282
## T1sf_GeneralHealth  2.930e-02  8.159e-03  6.552e+01   3.592  0.00063 ***
## WPlagstepsRC:Age    1.786e-03  2.380e-03  1.109e+03   0.750  0.45315
## ---
## Signif. codes:  0 '***' 0.001 '**' 0.01 '*' 0.05 '.' 0.1 ' ' 1
##
## Correlation of Fixed Effects:
##             (Intr) WPlgRC Age    Gender lgEnrg PMstRC Condtn EdYrs  T1s_GH
## WPlagstpsRC -0.019
## Age         -0.573  0.023
## Gender      -0.368  0.002 -0.039
## lagEnergy   -0.058  0.045  0.006  0.060
## PMstepsRC   -0.268 -0.012  0.020  0.266  0.005
## Condition   -0.152 -0.012  0.036  0.106  0.008  0.135
## EdYrs       -0.439 -0.008 -0.230  0.011 -0.064  0.029  0.068
## T1sf_GnrlHl -0.165  0.005 -0.169 -0.098 -0.131 -0.199 -0.198  0.011
## WPlgstpRC:A  0.021 -0.992 -0.022 -0.005 -0.065  0.010  0.013  0.007 -0.004
```

```
write.csv(stepmate, file = "ITTlag.csv")
```
