# Supplementary material for: Walking and Daily Affect Among Sedentary Older Adults Measured Using the StepMATE App: Pilot Randomized Controlled Trial
Source: JMIR Mhealth Uhealth. 2021 Dec 1;9(12):e27208. doi: 10.2196/27208 (PMC8686479; doi:10.2196/27208)
Supplement: Multimedia Appendix 6 [file mhealth_v9i12e27208_app6.pdf]

CONSORT-EHEALTH (V 1.6.1) - Submission/Publication Form

The CONSORT-EHEALTH checklist is intended for authors of randomized trials evaluating web-based and Internet-based applications/interventions, including mobile interventions, electronic games (incl multiplayer games), social media, certain telehealth applications, and other interactive and/or networked electronic applications. Some of the items (e.g. all subitems under item 5 - description of the intervention) may also be applicable for other study designs.

The goal of the CONSORT EHEALTH checklist and guideline is to be
a) a guide for reporting for authors of RCTs,
b) to form a basis for appraisal of an ehealth trial (in terms of validity)

CONSORT-EHEALTH items/subitems are MANDATORY reporting items for studies published in the Journal of Medical Internet Research and other journals / scientific societies endorsing the checklist.

Items numbered 1., 2., 3., 4a., 4b etc are original CONSORT or CONSORT-NPT (non-pharmacologic treatment) items.
Items with Roman numerals (i., ii, iii, iv etc.) are CONSORT-EHEALTH extensions/clarifications.

As the CONSORT-EHEALTH checklist is still considered in a formative stage, we would ask that you also RATE ON A SCALE OF 1-5 how important/useful you feel each item is FOR THE PURPOSE OF THE CHECKLIST and reporting guideline (optional).

Mandatory reporting items are marked with a red *.
In the textboxes, either copy & paste the relevant sections from your manuscript into this form - please include any quotes from your manuscript in QUOTATION MARKS,
or answer directly by providing additional information not in the manuscript, or elaborating on why the item was not relevant for this study.

YOUR ANSWERS WILL BE PUBLISHED AS A SUPPLEMENTARY FILE TO YOUR PUBLICATION IN JMIR AND ARE CONSIDERED PART OF YOUR PUBLICATION (IF ACCEPTED).
Please fill in these questions diligently. Information will not be copyedited, so please use proper spelling and grammar, use correct capitalization, and avoid abbreviations.

DO NOT FORGET TO SAVE AS PDF _AND_ CLICK THE SUBMIT BUTTON SO YOUR ANSWERS ARE IN OUR DATABASE !!!

Citation Suggestion (if you append the pdf as Appendix we suggest to cite this paper in the caption):
Eysenbach G, CONSORT-EHEALTH Group
CONSORT-EHEALTH: Improving and Standardizing Evaluation Reports of Web-based and

Mobile Health Interventions
J Med Internet Res 2011;13(4):e126
URL: http://www.jmir.org/2011/4/e126/
doi: 10.2196/jmir.1923
PMID: 22209829

* Required

Your name *

First Last

Alycia Bisson

Primary Affiliation (short), City, Country *

University of Toronto, Toronto, Canada

Brandeis University, Waltham, United States

Your e-mail address *

abc@gmail.com

alyciansullivan@brandeis.edu

Title of your manuscript *

Provide the (draft) title of your manuscript.

Walking and Daily Affect: Results from the StepMATE (Mobile App for Tracking Exercise) Randomized Control Trial

Name of your App/Software/Intervention *

If there is a short and a long/alternate name, write the short name first and add the long name in brackets.

StepMATE (Mobile App for Tracking Exercise)

Evaluated Version (if any)

e.g. "V1", "Release 2017-03-01", "Version 2.0.27913"

Your answer

Language(s) *

What language is the intervention/app in? If multiple languages are available, separate by comma (e.g. "English, French")

English

URL of your Intervention Website or App

e.g. a direct link to the mobile app on app in appstore (itunes, Google Play), or URL of the website. If the intervention is a DVD or hardware, you can also link to an Amazon page.

Your answer

URL of an image/screenshot (optional)

Your answer

Accessibility *

Can an enduser access the intervention presently?

- ◯ access is free and open
- ◯ access only for special usergroups, not open
- ◯ access is open to everyone, but requires payment/subscription/in-app purchases
- ◉ app/intervention no longer accessible
- ◯ Other:

Primary Medical Indication/Disease/Condition *

e.g. "Stress", "Diabetes", or define the target group in brackets after the condition, e.g. "Autism (Parents of children with)", "Alzheimers (Informal Caregivers of)"

Physical Inactivity

Primary Outcomes measured in trial *

comma-separated list of primary outcomes reported in the trial

Physical Activity (daily steps), Daily mood, Dail

Secondary/other outcomes

Are there any other outcomes the intervention is expected to affect?

Sleep, cognitive performance, social engagement, exercise control, exercise self-efficacy

Recommended "Dose" *

What do the instructions for users say on how often the app should be used?

- (selected) Approximately Daily
- Approximately Weekly
- Approximately Monthly
- Approximately Yearly
- "as needed"
- Other:

Approx. Percentage of Users (starters) still using the app as recommended after 3 months *

- (selected) unknown / not evaluated
- 0-10%
- 11-20%
- 21-30%
- 31-40%
- 41-50%
- 51-60%
- 61-70%
- 71%-80%
- 81-90%
- 91-100%
- Other:

Overall, was the app/intervention effective? *

- ( ) yes: all primary outcomes were significantly better in intervention group vs control
- ( ) partly: SOME primary outcomes were significantly better in intervention group vs control
- ( ) no statistically significant difference between control and intervention
- ( ) potentially harmful: control was significantly better than intervention in one or more outcomes
- ( ) inconclusive: more research is needed
- (x) Other: Some primary outcomes improved for both the intervention and contr

Article Preparation Status/Stage *

At which stage in your article preparation are you currently (at the time you fill in this form)

- ( ) not submitted yet - in early draft status
- ( ) not submitted yet - in late draft status, just before submission
- ( ) submitted to a journal but not reviewed yet
- (x) submitted to a journal and after receiving initial reviewer comments
- ( ) submitted to a journal and accepted, but not published yet
- ( ) published
- ( ) Other:

Journal *

If you already know where you will submit this paper (or if it is already submitted), please provide the journal name (if it is not JMIR, provide the journal name under "other")

- ○ not submitted yet / unclear where I will submit this
- ○ Journal of Medical Internet Research (JMIR)
- ◉ JMIR mHealth and UHealth
- ○ JMIR Serious Games
- ○ JMIR Mental Health
- ○ JMIR Public Health
- ○ JMIR Formative Research
- ○ Other JMIR sister journal
- ○ Other:

Is this a full powered effectiveness trial or a pilot/feasibility trial? *

- ◉ Pilot/feasibility
- ○ Fully powered

Manuscript tracking number *

If this is a JMIR submission, please provide the manuscript tracking number under "other" (The ms tracking number can be found in the submission acknowledgement email, or when you login as author in JMIR. If the paper is already published in JMIR, then the ms tracking number is the four-digit number at the end of the DOI, to be found at the bottom of each published article in JMIR)

- ○ no ms number (yet) / not (yet) submitted to / published in JMIR
- ◉ Other: JMU ms#27208

TITLE AND ABSTRACT

1a) TITLE: Identification as a randomized trial in the title

1a) Does your paper address CONSORT item 1a? *

I.e does the title contain the phrase "Randomized Controlled Trial"? (if not, explain the reason under "other")

- (x) yes
- ( ) Other:

1a-i) Identify the mode of delivery in the title

Identify the mode of delivery. Preferably use “web-based” and/or “mobile” and/or “electronic game” in the title. Avoid ambiguous terms like “online”, “virtual”, “interactive”. Use “Internet-based” only if Intervention includes non-web-based Internet components (e.g. email), use “computer-based” or “electronic” only if offline products are used. Use “virtual” only in the context of “virtual reality” (3-D worlds). Use “online” only in the context of “online support groups”. Complement or substitute product names with broader terms for the class of products (such as “mobile” or “smart phone” instead of “iphone”), especially if the application runs on different platforms.

	1	2	3	4	5	
subitem not at all important	( )	( )	( )	(x)	( )	essential

Clear selection

Does your paper address subitem 1a-i? *

Copy and paste relevant sections from manuscript title (include quotes in quotation marks "like this" to indicate direct quotes from your manuscript), or elaborate on this item by providing additional information not in the ms, or briefly explain why the item is not applicable/relevant for your study

"Walking and Daily Affect: Results from the StepMATE (Mobile App for Tracking Exercise) Randomized Control Trial"

1a-ii) Non-web-based components or important co-interventions in title

Mention non-web-based components or important co-interventions in title, if any (e.g., “with telephone support”).

	1	2	3	4	5	
subitem not at all important	(x)	( )	( )	( )	( )	essential

Clear selection

Does your paper address subitem 1a-ii?

Copy and paste relevant sections from manuscript title (include quotes in quotation marks "like this" to indicate direct quotes from your manuscript), or elaborate on this item by providing additional information not in the ms, or briefly explain why the item is not applicable/relevant for your study

Included in the manuscript.

"StepMATE was a fully automated app that included behavioral supports to help people plan where and when to walk, and social supports to help find others who might want to walk with them."

1a-iii) Primary condition or target group in the title

Mention primary condition or target group in the title, if any (e.g., “for children with Type I Diabetes”) Example: A Web-based and Mobile Intervention with Telephone Support for Children with Type I Diabetes: Randomized Controlled Trial

	1	2	3	4	5	
subitem not at all important	( )	(x)	( )	( )	( )	essential

Clear selection

Does your paper address subitem 1a-iii? *

Copy and paste relevant sections from manuscript title (include quotes in quotation marks "like this" to indicate direct quotes from your manuscript), or elaborate on this item by providing additional information not in the ms, or briefly explain why the item is not applicable/relevant for your study

Not in title; sample was healthy adults.

Target group described in the abstract and manuscript.

"... in a sample of inactive middle aged and older adults"

1b) ABSTRACT: Structured summary of trial design, methods, results, and conclusions

NPT extension: Description of experimental treatment, comparator, care providers, centers, and blinding status.

1b-i) Key features/functionalities/components of the intervention and comparator in the METHODS section of the ABSTRACT

Mention key features/functionalities/components of the intervention and comparator in the abstract. If possible, also mention theories and principles used for designing the site. Keep in mind the needs of systematic reviewers and indexers by including important synonyms. (Note: Only report in the abstract what the main paper is reporting. If this information is missing from the main body of text, consider adding it)

	1	2	3	4	5	
subitem not at all important	○	○	○	○	◉	essential

Clear selection

Does your paper address subitem 1b-i? *

Copy and paste relevant sections from the manuscript abstract (include quotes in quotation marks "like this" to indicate direct quotes from your manuscript), or elaborate on this item by providing additional information not in the ms, or briefly explain why the item is not applicable/relevant for your study

"They were randomly assigned to receive either a basic, pedometer-like version of the app, or a version with supports to help participants determine where, when, and with whom to walk."

1b-ii) Level of human involvement in the METHODS section of the ABSTRACT

Clarify the level of human involvement in the abstract, e.g., use phrases like “fully automated” vs. “therapist/nurse/care provider/physician-assisted” (mention number and expertise of providers involved, if any). (Note: Only report in the abstract what the main paper is reporting. If this information is missing from the main body of text, consider adding it)

	1	2	3	4	5	
subitem not at all important	○	○	○	◉	○	essential

Clear selection

Does your paper address subitem 1b-ii?

Copy and paste relevant sections from the manuscript abstract (include quotes in quotation marks "like this" to indicate direct quotes from your manuscript), or elaborate on this item by providing additional information not in the ms, or briefly explain why the item is not applicable/relevant for your study

Manuscript updated to reflect this item.

"The current pilot study tested the efficacy of a new app, StepMATE (Mobile App for Tracking Exercise) for increasing daily walking in a sample of inactive middle aged and older adults. We also examined the daily relationships between walking and self-reported mood and energy levels."

1b-iii) Open vs. closed, web-based (self-assessment) vs. face-to-face assessments in the METHODS section of the ABSTRACT

Mention how participants were recruited (online vs. offline), e.g., from an open access website or from a clinic or a closed online user group (closed usergroup trial), and clarify if this was a purely web-based trial, or there were face-to-face components (as part of the intervention or for assessment). Clearly say if outcomes were self-assessed through questionnaires (as common in web-based trials). Note: In traditional offline trials, an open trial (open-label trial) is a type of clinical trial in which both the researchers and participants know which treatment is being administered. To avoid confusion, use “blinded” or “unblinded” to indicated the level of blinding instead of “open”, as “open” in web-based trials usually refers to “open access” (i.e. participants can self-enrol). (Note: Only report in the abstract what the main paper is reporting. If this information is missing from the main body of text, consider adding it)

	1	2	3	4	5	
subitem not at all important	○	○	○	○	◉	essential

Clear selection

Does your paper address subitem 1b-iii?

Copy and paste relevant sections from the manuscript abstract (include quotes in quotation marks "like this" to indicate direct quotes from your manuscript), or elaborate on this item by providing additional information not in the ms, or briefly explain why the item is not applicable/relevant for your study

"Participants were adults 50 years old and over (Mage = 61.64, SD = 7.67). They were randomly assigned to receive either a basic, pedometer-like version of the app, or a version with supports to help participants determine where, when, and with whom to walk. Upon downloading the app, step data from the week prior were automatically recorded. Participants in both groups were asked to set a daily walking goal, which they could change at any point during the intervention. They were asked to use the app as much as possible over the next 4 weeks. Twice per day, popup notifications assessed mood and energy levels."

1b-iv) RESULTS section in abstract must contain use data

Report number of participants enrolled/assessed in each group, the use/uptake of the intervention (e.g., attrition/adherence metrics, use over time, number of logins etc.), in addition to primary/secondary outcomes. (Note: Only report in the abstract what the main paper is reporting. If this information is missing from the main body of text, consider adding it)

	1	2	3	4	5	
subitem not at all important	○	○	○	○	●	essential

Clear selection

Does your paper address subitem 1b-iv?

Copy and paste relevant sections from the manuscript abstract (include quotes in quotation marks "like this" to indicate direct quotes from your manuscript), or elaborate on this item by providing additional information not in the ms, or briefly explain why the item is not applicable/relevant for your study

Abstract modified.

"Although one group had access to additional app features, both groups used the app in a similar way, mainly using just the walk-tracking feature. Multilevel models revealed that both groups took significantly more steps during the 4-week study than the week before downloading the app, ($\gamma = 0.24$, $p < .001$). During the study, participants in both groups averaged 5,248 steps per day, compared to an average of 3,753 steps taken per day in the baseline week. Contrary to predictions, there were no differences in step increases between the two conditions. Initial step increases were maintained but did not increase further over the study. Cognition significantly improved from pre- to post-test, ($\gamma = 0.17$, $p = .023$). Across conditions, on days in which participants took more steps than average, they reported better mood and higher energy levels on the same day, and better mood on the subsequent day. Daily associations between walking, mood, and energy were significant for women, but not men and were stronger for older participants (those 62 years old and older), than younger participants."

1b-v) CONCLUSIONS/DISCUSSION in abstract for negative trials

Conclusions/Discussions in abstract for negative trials: Discuss the primary outcome - if the trial is negative (primary outcome not changed), and the intervention was not used, discuss whether negative results are attributable to lack of uptake and discuss reasons. (Note: Only report in the abstract what the main paper is reporting. If this information is missing from the main body of text, consider adding it)

	1	2	3	4	5	
subitem not at all important	○	○	○	○	◉	essential

Clear selection

Does your paper address subitem 1b-v?

Copy and paste relevant sections from the manuscript abstract (include quotes in quotation marks "like this" to indicate direct quotes from your manuscript), or elaborate on this item by providing additional information not in the ms, or briefly explain why the item is not applicable/relevant for your study

"The behavioral features included in the app were not more beneficial than the basic version for increasing daily walking, likely because the treatment group rarely used the additional features available to them. Both groups increased their steps to a similar extent, suggesting that setting and monitoring daily walking goals was sufficient for an initial increase and maintenance of steps. Across conditions, walking had benefits for positive mood and energy level on the same day, particularly for women and older participants. The effects of steps on mood also lasted into the next day. Further investigations should identify other motivating factors that could lead to greater and more sustained increases in physical activity."

INTRODUCTION

2a) In INTRODUCTION: Scientific background and explanation of rationale

2a-i) Problem and the type of system/solution

Describe the problem and the type of system/solution that is object of the study: intended as stand-alone intervention vs. incorporated in broader health care program? Intended for a particular patient population? Goals of the intervention, e.g., being more cost-effective to other interventions, replace or complement other solutions? (Note: Details about the intervention are provided in “Methods” under 5)

	1	2	3	4	5	
subitem not at all important	○	○	○	○	◉	essential

Clear selection

Does your paper address subitem 2a-i? *

Copy and paste relevant sections from the manuscript (include quotes in quotation marks "like this" to indicate direct quotes from your manuscript), or elaborate on this item by providing additional information not in the ms, or briefly explain why the item is not applicable/relevant for your study

"The benefits of physical activity for lifelong health, well-being, and cognition are well-documented, yet the majority of American adults lead an inactive lifestyle[1]–[3]. According to the Centers for Disease Control (CDC), 53.3% of adults meet the guidelines for aerobic activity[4]. The percentage of adults who meet these guidelines drops with increasing age; only 32.3% of adults 75 and older meet these guidelines[4]. Fitness technologies, such as Fitbit, Apple watch, or smartphone apps have the potential to track and encourage physical activity without the need for additional equipment or a gym membership. Indeed, the 'health and fitness' category is one of the most popular categories in the iTunes and Google Play app stores, with almost 230,000 apps available in 2017.
A recent review of 37 Fitbit-based interventions reported that these studies were associated with increases in daily steps, moderate to vigorous physical activity, and decreases in body weight[5]. Participants in these interventions took about 950 steps per day more than control groups who were not given a Fitbit. The behavioral components included in the given interventions were related to the success of the interventions[5]. Goal setting was described as the most promising intervention tool, while messaging was less effective. The authors suggested that a combination of intervention tools may be necessary to encourage changes in physical activity[5]. Another recent review suggested that there is little to moderate evidence that mobile or electronic health interventions are successful for increasing physical activity in older adults[6]. However, they reported that techniques like self-monitoring, behavior change techniques, and social support may improve the effectiveness of mobile health interventions.
While many devices and smartphone apps currently track physical activity, encourage one to meet step goals, and link with other personal data, few stand-alone smartphone apps include additional features that address barriers unique to inactive adults[7]. Further, apps that do include behavior change strategies typically cost more money and do not provide features like action planning and environmental supports. Focus groups have identified a need for physical activity apps to promote autonomy and self-regulation, while also providing adaptability and flexibility to accommodate individual needs[8].
Implementation intentions involve behavioral strategies such as creating a specific plan to reach a goal[9]. Using walking as an example, implementation intentions could include action planning; creating a plan that includes the time and place that walking would occur[10]. Meta analyses have shown that implementation intentions, including action planning, are associated with physical activity increases[11], [12]. A recent study tested whether an implementation intentions intervention was more successful in increasing physical activity than just using a Fitbit[13]. Compared to those who only wore a Fitbit, the intervention group who were given step goals, personalized walking routes, and a daily schedule to fill out, significantly increased their daily steps over the course of one month[13]. Although action planning and environmental supports are rarely incorporated into fitness technology, such strategies may directly address common barriers that prevent adults from engaging in physical activity[7]. "

2a-ii) Scientific background, rationale: What is known about the (type of) system

Scientific background, rationale: What is known about the (type of) system that is the object of the study (be sure to discuss the use of similar systems for other conditions/diagnoses, if appropiate), motivation for the study, i.e. what are the reasons for and what is the context for this specific study, from which stakeholder viewpoint is the study performed, potential impact of findings [2]. Briefly justify the choice of the comparator.

	1	2	3	4	5	
subitem not at all important	○	○	○	○	◉	essential

Clear selection

Does your paper address subitem 2a-ii? *

Copy and paste relevant sections from the manuscript (include quotes in quotation marks "like this" to indicate direct quotes from your manuscript), or elaborate on this item by providing additional information not in the ms, or briefly explain why the item is not applicable/relevant for your study

"Along with the benefits of exercise to physical and cognitive health, many have shown the importance of physical activity to mood and affective states[14], [15]. In fact, a recent meta-analysis reported that improved executive functioning and mood, along with decreases in stress are among the most consistently reported outcomes following acute exercise[14]. These effects have been echoed in multiple populations and various activity domains, including vigorous activities like cycling and lower-intensity activities like yoga or walking. One study showed that patients with multiple sclerosis were more likely to report improved mood following a single 20 minute bout of walking or yoga than a 20 minute period of rest[16]. Others have also found that positive exercise experiences are linked to increases in motivational self-efficacy and exercise intentions, which then predict future exercise behavior[17].

While early research has shown the importance of physical activity for mood and affect in controlled laboratory settings, new technologies have made it possible to examine these relationships in real-time, using accelerometry along with methods like experience sampling or Ecological Momentary Assessments (EMAs). One recent study used a newly developed smartphone app and EMA to examine relationships between self-reported happiness and physical activity levels[18]. Results from over 10,000 users of the app showed that people who are more active overall report greater happiness than less active individuals. They also examined within-person fluctuations and found that people were happier on more physically active days compared to less active ones[18].

A review paper by Liao and colleagues (2015) summarized 14 studies that used EMA to examine short-term relationships between physical activity and affect. They found evidence for reciprocal relationships, where current positive affect predicted increased physical activity within the next few hours, and physical activity engagement predicted greater positive affect within the next few hours[19]. Thus, it appears that positive affect predicts subsequent physical activity, which also predicts future positive affect.

It is possible that men and women experience differential effects of exercise on mood, however, very little work has examined these sex differences. One study found that in young adults, females were more likely to report improvements in mood following exercise than males[20]. The same study found that women were more likely than men to report reduced fatigue following a 30-minute bout of exercise[20]. Thus, it is possible that women are more sensitive to mood changes following exercise.

In sum, physical activity and affect have been linked at both the within- and between-person level. These effects are similar across various domains of physical activity, and in healthy and non-healthy adult populations. Affective changes can be seen from acute (20 minute) bouts of activity, to regular activity over the course of months. Increases in positive affect following exercise are likely to improve exercise self-efficacy and subsequently predict future physical activity. While prior studies have examined affective improvements in the context of structured exercise, no studies to our knowledge have tested whether the number of steps one takes per day is predictive of contemporaneous changes in affect. Further, few have closely examined whether these effects differ between men and women.

Physical Activity, Sleep, and Energy

Physical Activity, Sleep, and Energy

Another consistent finding in the literature is the relationship between physical activity and sleep. When examining average levels of physical activity, those who are more active tend to sleep better than less active people[21]. Most of this work has focused on high impact physical activity, or populations with sleep disorders or other health problems. Daily studies suggest that on days in which one is more active, they tend to sleep better and longer than less active days[22]–[24]. Recent work found that women who average more steps per day over the course of the month reported better sleep quality than inactive women[22]. Unexpectedly, daily steps were not associated with self-reports of sleep quality for men.

One study examined the relationship between physical activity, affect, and insomnia symptoms in a sample of inactive adults with insomnia[25]. Compared to the control group who were asked to maintain their baseline physical activity levels, those who engaged in consistent walking reported significant decreases in insomnia symptoms, along with improved affect over the six month intervention[25]. Taken together, these results suggest that even low impact physical activity such as walking or yoga has the potential to improve sleep in adults. Those who sleep well likely will report higher energy levels during the day; however, self-reports of energy are also affected by other things that happen on any given day. Though the link between physical activity and sleep has been studied, less is known about how daily physical activity is related to self-reported daily energy levels."

2b) In INTRODUCTION: Specific objectives or hypotheses

Does your paper address CONSORT subitem 2b? *

Copy and paste relevant sections from the manuscript (include quotes in quotation marks "like this" to indicate direct quotes from your manuscript), or elaborate on this item by providing additional information not in the ms, or briefly explain why the item is not applicable/relevant for your study

"Participants in the StepMATE pilot randomized control trial (RCT) were randomly assigned to receive one of two versions of the app. The control group was only given the step-tracking and goal-setting functions, similar to a Fitbit, and the treatment group was given a version of the app with additional social and environmental supports. First, we tested whether the app was associated with increases in average daily steps over the four weeks, and whether there were differences between the two conditions. It was predicted that the additional supports would help the treatment group increase their daily walking more than the control condition. Next, we examined whether there were changes over time or between-group differences in other outcomes, including sleep, exercise control, exercise self-efficacy, social engagement, and memory. We predicted that participants would report improvements in these outcomes from pre-to post-test, with greater improvements in the treatment group. Finally, within-person relationships between daily steps and self-reported mood and energy were modeled. We predicted that on days when participants took more steps than average, they would report higher energy levels and better mood than on less active days. Drawing from prior findings on sex differences in daily relationships between exercise and other outcomes, interactions between daily steps and sex on mood and energy were examined. It was predicted that daily steps would be more closely related to mood and energy in women than men. Exploratory analyses examined whether there were interactions between daily steps and age in predicting mood and energy."

METHODS

3a) Description of trial design (such as parallel, factorial) including allocation ratio

Does your paper address CONSORT subitem 3a? *

Copy and paste relevant sections from the manuscript (include quotes in quotation marks "like this" to indicate direct quotes from your manuscript), or elaborate on this item by providing additional information not in the ms, or briefly explain why the item is not applicable/relevant for your study

Manuscript updated.

"Participants were recruited online on a rolling basis between January 2018 and March 2019 using Facebook, Craigslist, and FindParticipants. Participants were also recruited locally throughout eastern and central Massachusetts via flyers at senior centers, libraries, cafes, and community events. Because the study required no in-person meeting, participants were recruited from locations across the continental United States."

3b) Important changes to methods after trial commencement (such as eligibility criteria), with reasons

Does your paper address CONSORT subitem 3b? *

Copy and paste relevant sections from the manuscript (include quotes in quotation marks "like this" to indicate direct quotes from your manuscript), or elaborate on this item by providing additional information not in the ms, or briefly explain why the item is not applicable/relevant for your study

No changes were made to methods after trial commencement

3b-i) Bug fixes, Downtimes, Content Changes

Bug fixes, Downtimes, Content Changes: ehealth systems are often dynamic systems. A description of changes to methods therefore also includes important changes made on the intervention or comparator during the trial (e.g., major bug fixes or changes in the functionality or content) (5-iii) and other “unexpected events” that may have influenced study design such as staff changes, system failures/downtimes, etc. [2].

	1	2	3	4	5	
subitem not at all important	○	○	○	○	◉	essential

Clear selection

Does your paper address subitem 3b-i?

Copy and paste relevant sections from the manuscript (include quotes in quotation marks "like this" to indicate direct quotes from your manuscript), or elaborate on this item by providing additional information not in the ms, or briefly explain why the item is not applicable/relevant for your study

Manuscript updated.

"Following an iPhone software update, the StepMATE app crashed and did not work properly for approximately two weeks. There were 18 participants affected, and daily step data was lost for these participants."

4a) Eligibility criteria for participants

Does your paper address CONSORT subitem 4a? *

Copy and paste relevant sections from the manuscript (include quotes in quotation marks "like this" to indicate direct quotes from your manuscript), or elaborate on this item by providing additional information not in the ms, or briefly explain why the item is not applicable/relevant for your study

"Participants were required to own an iPhone with a built-in accelerometer to measure steps (iPhone 5S or newer). Only those who reported exercising less than the CDC guidelines of 150 minutes of moderate to vigorous exercise per week were eligible, they also needed to report walking for exercise no more than 30 minutes per day[35]. Participants were ineligible if a doctor advised them not to walk due to health conditions, or if they had a cardiac event or fall within the last six months. Screening for cognitive impairment was done over the phone using a shortened version of the Short Portable Mental Status Questionnaire (SPMSQ)[36]. Participants were ineligible if they made three or more errors on the SPMSQ."

4a-i) Computer / Internet literacy

Computer / Internet literacy is often an implicit “de facto” eligibility criterion - this should be explicitly clarified.

	1	2	3	4	5	
subitem not at all important	◉	○	○	○	○	essential

Clear selection

Does your paper address subitem 4a-i?

Copy and paste relevant sections from the manuscript (include quotes in quotation marks "like this" to indicate direct quotes from your manuscript), or elaborate on this item by providing additional information not in the ms, or briefly explain why the item is not applicable/relevant for your study

Computer or internet literacy was not an eligibility criteria.

4a-ii) Open vs. closed, web-based vs. face-to-face assessments:

Open vs. closed, web-based vs. face-to-face assessments: Mention how participants were recruited (online vs. offline), e.g., from an open access website or from a clinic, and clarify if this was a purely web-based trial, or there were face-to-face components (as part of the intervention or for assessment), i.e., to what degree got the study team to know the participant. In online-only trials, clarify if participants were quasi-anonymous and whether having multiple identities was possible or whether technical or logistical measures (e.g., cookies, email confirmation, phone calls) were used to detect/prevent these.

	1	2	3	4	5	
subitem not at all important	○	○	○	○	◉	essential

Clear selection

Does your paper address subitem 4a-ii? *

Copy and paste relevant sections from the manuscript (include quotes in quotation marks "like this" to indicate direct quotes from your manuscript), or elaborate on this item by providing additional information not in the ms, or briefly explain why the item is not applicable/relevant for your study

"Participants were recruited online on a rolling basis between January 2018 and March 2019 using Facebook, Craigslist, and FindParticipants. Participants were also recruited locally throughout eastern and central Massachusetts via flyers at senior centers, libraries, cafes, and community events. Because the study required no in-person meeting, participants were recruited from locations across the continental United States."

4a-iii) Information giving during recruitment

Information given during recruitment. Specify how participants were briefed for recruitment and in the informed consent procedures (e.g., publish the informed consent documentation as appendix, see also item X26), as this information may have an effect on user self-selection, user expectation and may also bias results.

	1	2	3	4	5	
subitem not at all important	○	○	○	○	◉	essential

Clear selection

Does your paper address subitem 4a-iii?

Copy and paste relevant sections from the manuscript (include quotes in quotation marks "like this" to indicate direct quotes from your manuscript), or elaborate on this item by providing additional information not in the ms, or briefly explain why the item is not applicable/relevant for your study

The informed consent documents are included on clinicaltrials.gov.

4b) Settings and locations where the data were collected

Does your paper address CONSORT subitem 4b? *

Copy and paste relevant sections from the manuscript (include quotes in quotation marks "like this" to indicate direct quotes from your manuscript), or elaborate on this item by providing additional information not in the ms, or briefly explain why the item is not applicable/relevant for your study

"Participants were also recruited locally throughout eastern and central Massachusetts via flyers at senior centers, libraries, cafes, and community events. Because the study required no in-person meeting, participants were recruited from locations across the continental United States."

4b-i) Report if outcomes were (self-)assessed through online questionnaires

Clearly report if outcomes were (self-)assessed through online questionnaires (as common in web-based trials) or otherwise.

	1	2	3	4	5	
subitem not at all important	○	○	○	○	◉	essential

Clear selection

Does your paper address subitem 4b-i? *

Copy and paste relevant sections from the manuscript (include quotes in quotation marks "like this" to indicate direct quotes from your manuscript), or elaborate on this item by providing additional information not in the ms, or briefly explain why the item is not applicable/relevant for your study

Manuscript modified.

"Then participants were administered a reduced version of the Brief Test of Adult Cognition by Telephone (BTACT) over the phone and completed the pre-study questionnaires online via Qualtrics, including self-assessed social engagement, exercise control, exercise self-efficacy, and sleep. Participants filled out their ID number at the beginning of the Qualtrics survey so their self-report data could easily be linked to their step data."

4b-ii) Report how institutional affiliations are displayed

Report how institutional affiliations are displayed to potential participants [on ehealth media], as affiliations with prestigious hospitals or universities may affect volunteer rates, use, and reactions with regards to an intervention.(Not a required item – describe only if this may bias results)

	1	2	3	4	5	
subitem not at all important	○	◉	○	○	○	essential

Clear selection

Does your paper address subitem 4b-ii?

Copy and paste relevant sections from the manuscript (include quotes in quotation marks "like this" to indicate direct quotes from your manuscript), or elaborate on this item by providing additional information not in the ms, or briefly explain why the item is not applicable/relevant for your study

Informed consent documents are included on clinicaltrials.gov.

5) The interventions for each group with sufficient details to allow replication, including how and when they were actually administered

5-i) Mention names, credential, affiliations of the developers, sponsors, and owners

Mention names, credential, affiliations of the developers, sponsors, and owners [6] (if authors/evaluators are owners or developer of the software, this needs to be declared in a “Conflict of interest” section or mentioned elsewhere in the manuscript).

	1	2	3	4	5	
subitem not at all important	○	○	○	○	◉	essential

Clear selection

Does your paper address subitem 5-i?

Copy and paste relevant sections from the manuscript (include quotes in quotation marks "like this" to indicate direct quotes from your manuscript), or elaborate on this item by providing additional information not in the ms, or briefly explain why the item is not applicable/relevant for your study

Manuscript modified.

"The StepMATE app was developed by Beneufit using Apple ResearchKit, with feedback from University researchers."

5-ii) Describe the history/development process

Describe the history/development process of the application and previous formative evaluations (e.g., focus groups, usability testing), as these will have an impact on adoption/use rates and help with interpreting results.

	1	2	3	4	5	
subitem not at all important	○	○	○	○	●	essential

Clear selection

Does your paper address subitem 5-ii?

Copy and paste relevant sections from the manuscript (include quotes in quotation marks "like this" to indicate direct quotes from your manuscript), or elaborate on this item by providing additional information not in the ms, or briefly explain why the item is not applicable/relevant for your study

"Based on our previous study in midlife adults that assessed barriers to being physically active[13], along with findings from pilot interviews of 9 older adults, we found that perceived lack of time was a common barrier preventing people from getting enough exercise. Other reported barriers reported in these studies were not knowing where to exercise, and not wanting to exercise alone. The StepMATE app was developed by Beneufit using Apple ResearchKit, with feedback from University researchers. StepMATE was a fully automated app that included behavioral supports to help people plan where and when to walk, and social supports to help find others who might want to walk with them."

5-iii) Revisions and updating

Revisions and updating. Clearly mention the date and/or version number of the application/intervention (and comparator, if applicable) evaluated, or describe whether the intervention underwent major changes during the evaluation process, or whether the development and/or content was “frozen” during the trial. Describe dynamic components such as news feeds or changing content which may have an impact on the replicability of the intervention (for unexpected events see item 3b).

	1	2	3	4	5	
subitem not at all important	(•)	( )	( )	( )	( )	essential

Clear selection

Does your paper address subitem 5-iii?

Copy and paste relevant sections from the manuscript (include quotes in quotation marks "like this" to indicate direct quotes from your manuscript), or elaborate on this item by providing additional information not in the ms, or briefly explain why the item is not applicable/relevant for your study

There were no major changes to the app during the intervention

5-iv) Quality assurance methods

Provide information on quality assurance methods to ensure accuracy and quality of information provided [1], if applicable.

	1	2	3	4	5	
subitem not at all important	(•)	( )	( )	( )	( )	essential

Clear selection

Does your paper address subitem 5-iv?

Copy and paste relevant sections from the manuscript (include quotes in quotation marks "like this" to indicate direct quotes from your manuscript), or elaborate on this item by providing additional information not in the ms, or briefly explain why the item is not applicable/relevant for your study

Not applicable

5-v) Ensure replicability by publishing the source code, and/or providing screenshots/screen-capture video, and/or providing flowcharts of the algorithms used

Ensure replicability by publishing the source code, and/or providing screenshots/screen-capture video, and/or providing flowcharts of the algorithms used. Replicability (i.e., other researchers should in principle be able to replicate the study) is a hallmark of scientific reporting.

	1	2	3	4	5	
subitem not at all important	○	○	○	○	◉	essential

Clear selection

Does your paper address subitem 5-v?

Copy and paste relevant sections from the manuscript (include quotes in quotation marks "like this" to indicate direct quotes from your manuscript), or elaborate on this item by providing additional information not in the ms, or briefly explain why the item is not applicable/relevant for your study

The source codes used for data analysis (including results of analyses) in R are now uploaded as an appendix.

5-vi) Digital preservation

Digital preservation: Provide the URL of the application, but as the intervention is likely to change or disappear over the course of the years; also make sure the intervention is archived (Internet Archive, webcitation.org, and/or publishing the source code or screenshots/videos alongside the article). As pages behind login screens cannot be archived, consider creating demo pages which are accessible without login.

	1	2	3	4	5	
subitem not at all important	○	○	○	○	◉	essential

Clear selection

Does your paper address subitem 5-vi?

Copy and paste relevant sections from the manuscript (include quotes in quotation marks "like this" to indicate direct quotes from your manuscript), or elaborate on this item by providing additional information not in the ms, or briefly explain why the item is not applicable/relevant for your study

Screenshots of the app are included in the supplementary materials. Videos detailing app features by condition are now uploaded as an appendix.

5-vii) Access

Access: Describe how participants accessed the application, in what setting/context, if they had to pay (or were paid) or not, whether they had to be a member of specific group. If known, describe how participants obtained “access to the platform and Internet” [1]. To ensure access for editors/reviewers/readers, consider to provide a “backdoor” login account or demo mode for reviewers/readers to explore the application (also important for archiving purposes, see vi).

	1	2	3	4	5	
subitem not at all important	○	○	○	○	◉	essential

Clear selection

Does your paper address subitem 5-vii? *

Copy and paste relevant sections from the manuscript (include quotes in quotation marks "like this" to indicate direct quotes from your manuscript), or elaborate on this item by providing additional information not in the ms, or briefly explain why the item is not applicable/relevant for your study

"Researchers then scheduled a phone call to help participants download the app, setup their account including daily walking goals, and thoroughly explain the app features. Those in the control condition downloaded a version with only the daily step goals and the ability to track time, distance, and steps within a walk. The treatment condition had access to these and additional features, including schedules, maps, and social features."

5-viii) Mode of delivery, features/functionalities/components of the intervention and comparator, and the theoretical framework

Describe mode of delivery, features/functionalities/components of the intervention and comparator, and the theoretical framework [6] used to design them (instructional strategy [1], behaviour change techniques, persuasive features, etc., see e.g., [7, 8] for terminology). This includes an in-depth description of the content (including where it is coming from and who developed it) [1],” whether [and how] it is tailored to individual circumstances and allows users to track their progress and receive feedback” [6]. This also includes a description of communication delivery channels and – if computer-mediated communication is a component – whether communication was synchronous or asynchronous [6]. It also includes information on presentation strategies [1], including page design principles, average amount of text on pages, presence of hyperlinks to other resources, etc. [1].

	1	2	3	4	5	
subitem not at all important	○	○	○	○	◉	essential

Clear selection

Does your paper address subitem 5-viii? *

Copy and paste relevant sections from the manuscript (include quotes in quotation marks "like this" to indicate direct quotes from your manuscript), or elaborate on this item by providing additional information not in the ms, or briefly explain why the item is not applicable/relevant for your study

"The StepMATE app (Figure 1) was designed to help participants determine when, where, and with whom they would add physical activity into their day. Daily step goals were set by participants in both the intervention and control groups, and all participants had the ability to change their step goal at any point throughout the study. For the 'when' component, those in the intervention condition had a scheduling feature in the app. Participants could schedule a block of time to go for a walk, and had the option to create a reminder, set recurring events, and estimate the number of steps they would get in that walk. Once an event was created, it appeared in both the StepMATE app and in the iPhone's built-in calendar.
For the 'where' component, those in the intervention group were able to create, name, and save walking routes in the app. When participants in the intervention group hit 'Walk Now,' StepMATE began keeping track of their geographical location, distance, number of steps, and total time of the walk. This information was then saved after the walk was finished, so the time it takes to walk a route could be compared if it is walked again. When a user created multiple routes, they could be filtered by number of steps or duration, so one could easily find a walk that fits the amount of time they have, or the number of steps needed to achieve their daily walking goal. Those in the control group still had a 'walk now' button, however, when control participants hit walk now, the app would simply track the number of steps taken in that walk. These participants were not able to name or save their walks, nor could they view their walks on a map.
For the 'with whom' (social feature), those in the intervention condition had the option to text one of their iPhone contacts through the app and invite them for a walk. Those in the control condition did not have access to this feature. Supplementary materials include screenshots of the app, video tutorials of the app features and differences between versions, and descriptions of other various app functions."

5-ix) Describe use parameters

Describe use parameters (e.g., intended "doses" and optimal timing for use). Clarify what instructions or recommendations were given to the user, e.g., regarding timing, frequency, heaviness of use, if any, or was the intervention used ad libitum.

	1	2	3	4	5	
subitem not at all important	○	○	○	○	◉	essential

Clear selection

Does your paper address subitem 5-ix?

Copy and paste relevant sections from the manuscript (include quotes in quotation marks "like this" to indicate direct quotes from your manuscript), or elaborate on this item by providing additional information not in the ms, or briefly explain why the item is not applicable/relevant for your study

"Participants in both groups were asked to use the app for one month and do their best to answer the daily mood and energy questions. All participants were sent a pouch to wear around their waists and were encouraged to use it to carry their phone with them as much as possible until going to bed each night. After the first and third weeks, participants received an email letting them know how many weeks they had been in the study and how many remained. After the second week, researchers called participants to ask some open-ended feedback questions and ensure there were no problems with the app. If any problems arose during the course of the intervention, participants had access to a 'help' section within the app that included a phone number and email to contact researchers. This information was also included in the paper intervention materials that were mailed to them and attached to all email communications."

5-x) Clarify the level of human involvement

Clarify the level of human involvement (care providers or health professionals, also technical assistance) in the e-intervention or as co-intervention (detail number and expertise of professionals involved, if any, as well as "type of assistance offered, the timing and frequency of the support, how it is initiated, and the medium by which the assistance is delivered". It may be necessary to distinguish between the level of human involvement required for the trial, and the level of human involvement required for a routine application outside of a RCT setting (discuss under item 21 – generalizability).

	1	2	3	4	5	
subitem not at all important	○	○	○	◉	○	essential

Clear selection

Does your paper address subitem 5-x?

Copy and paste relevant sections from the manuscript (include quotes in quotation marks "like this" to indicate direct quotes from your manuscript), or elaborate on this item by providing additional information not in the ms, or briefly explain why the item is not applicable/relevant for your study

Manuscript modified.

"If any problems arose during the course of the intervention, participants had access to a ‘help’ section within the app that included a phone number and email to contact researchers. This information was also included in the paper intervention materials that were mailed to them and attached to all email communications."

5-xi) Report any prompts/reminders used

Report any prompts/reminders used: Clarify if there were prompts (letters, emails, phone calls, SMS) to use the application, what triggered them, frequency etc. It may be necessary to distinguish between the level of prompts/reminders required for the trial, and the level of prompts/reminders for a routine application outside of a RCT setting (discuss under item 21 – generalizability).

	1	2	3	4	5	
subitem not at all important	○	○	○	●	○	essential

Clear selection

Does your paper address subitem 5-xi? *

Copy and paste relevant sections from the manuscript (include quotes in quotation marks "like this" to indicate direct quotes from your manuscript), or elaborate on this item by providing additional information not in the ms, or briefly explain why the item is not applicable/relevant for your study

"Twice at random times each day, mood and energy levels were assessed. A popup notification asked participants to rate their current mood (unhappy, neutral, happy) and energy (low, neutral, high) on a slider scale."

5-xii) Describe any co-interventions (incl. training/support)

Describe any co-interventions (incl. training/support): Clearly state any interventions that are provided in addition to the targeted eHealth intervention, as ehealth intervention may not be designed as stand-alone intervention. This includes training sessions and support [1]. It may be necessary to distinguish between the level of training required for the trial, and the level of training for a routine application outside of a RCT setting (discuss under item 21 – generalizability.

	1	2	3	4	5	
subitem not at all important	○	○	○	○	◉	essential

Clear selection

Does your paper address subitem 5-xii? *

Copy and paste relevant sections from the manuscript (include quotes in quotation marks "like this" to indicate direct quotes from your manuscript), or elaborate on this item by providing additional information not in the ms, or briefly explain why the item is not applicable/relevant for your study

Materials sent to participants to familiarize themselves with the app and app features are included in the supplementary materials. Training videos that explain all app features are now also uploaded as an appendix.

6a) Completely defined pre-specified primary and secondary outcome measures, including how and when they were assessed

Does your paper address CONSORT subitem 6a? *

Copy and paste relevant sections from the manuscript (include quotes in quotation marks "like this" to indicate direct quotes from your manuscript), or elaborate on this item by providing additional information not in the ms, or briefly explain why the item is not applicable/relevant for your study

"Pre- and Post-Test Measures

Social Engagement
Social engagement was measured using the Lubben Social Network Scale (LSNS)[26]. The LSNS is comprised of 12 items (6 family and 6 friend), which ask about the size of one's social network (e.g., how many friends or family do you feel at ease with that you can talk about private matters?), and also the closeness of the relationships (e.g., how often do you see or hear from the friend or family member with whom you have the most contact?). A composite score was calculated by summing the responses of the 12 items, with a final score ranging from 0-60, where a higher score indicates more social engagement. The internal consistency in the current sample was Cronbach's a = .862.

Exercise Control
Control over exercise was measured using the 6-item Exercise Control Beliefs Scale[27]. Items assess one's beliefs about their control over exercise (e.g., I am confident in my ability to do an exercise routine), with answer choices ranging from strongly disagree (1) to strongly agree (5). The 6 items were averaged to create a mean exercise control score, with a higher score indicating greater control over exercise. Internal consistency in the current sample was Cronbach's a = .604

Exercise Self-Efficacy
A modified version of Bandura's Exercise Self-Efficacy (ESE) scale [28] was used in the current study. This 9-item scale assesses how sure one is that they would exercise under different conditions or constraints (e.g., How sure are you that you will exercise when you are feeling tired or under pressure to get things done?), with answer choices ranging from not sure at all (1) to very sure (4). The 9 items were averaged to create a composite score, where a higher score indicates greater exercise self-efficacy. Reliability of this scale was Cronbach's a = .935

Cognitive Performance
Cognition was assessed using a shortened version of the Brief Test of Adult Cognition by Telephone (BTACT)[29]. This version of the BTACT assesses five cognitive dimensions, including: two measures of episodic verbal memory (immediate and delayed free recall of 15 words), working memory (backwards digit span), verbal fluency (the number of words produced from a given category within 60 seconds), and processing speed (counting backwards from 100 in 30 seconds). The primary outcome measure was a composite of all cognitive tests. Test scores on both occasions were standardized based on scores at the pretest to create cognitive composites for the pretest and posttest. The BTACT is a reliable assessment of cognitive functioning with psychometric properties reported in another manuscript[29].

Sleep
Sleep duration and quality were measured using the Pittsburgh Sleep Quality Index (PSQI)

Sleep duration and quality were measured using the Pittsburgh Sleep Quality Index (PSQI) [30]. The PSQI global score could range from 0 to 21, with a higher score indicating worse sleep. Reliability for the 7 subscales of the PSQI was Cronbach's a = .77. In the current study, the PSQI global score, and raw scores for duration (number of hours slept, on average in the past month) and latency (number of minutes to fall asleep, on average during the past month) were examined.

Daily Measures

App Engagement
To assess the usage of various app features, for each participant, the total number of texts sent to contacts, number of routes saved, number of scheduled events, and number of times the 'Walk Now' feature was used were computed.

Physical Activity
Daily over the course of one month, physical activity was assessed using the total number of steps taken each day. Daily steps were quantified using the iPhone's built-in accelerometer and recorded via the StepMATE app. When participants downloaded StepMATE at the beginning of the study, the app automatically and retroactively recorded daily steps from the week prior to the start of the study. During the four-week intervention, participants were asked to carry their phone with them during the day, however, they were not specifically instructed to do so during the baseline week before the intervention began. While data indicate that older adults typically average between 2,000 and 9,000 steps per day [31], there are likely times participants walked without carrying their iPhone. For days when the iPhone recorded less than 500 steps, that day of steps was coded as missing. Weekly step averages were calculated for weeks with four or more days with 500 or more daily steps. Out of the 81 participants included in this intent-to-treat analysis, 11 participants had missing or incomplete baseline data.

Daily Affect: Mood and Energy
Twice at random times each day, mood and energy levels were assessed. A popup notification asked participants to rate their current mood (unhappy, neutral, happy) and energy (low, neutral, high) on a slider scale. Scores were converted by the StepMATE app to a 0-10 scale, with 0 indicating low mood/energy, and 10 indicating high mood/energy. The two daily ratings were averaged to provide a daily average of one's mood and energy. "

6a-i) Online questionnaires: describe if they were validated for online use and apply CHERRIES items to describe how the questionnaires were designed/deployed

If outcomes were obtained through online questionnaires, describe if they were validated for online use and apply CHERRIES items to describe how the questionnaires were designed/deployed [9].

	1	2	3	4	5	
subitem not at all important	○	○	○	◉	○	essential

Clear selection

Does your paper address subitem 6a-i?

Copy and paste relevant sections from manuscript text

Questionnaires in this study have been validated and proven reliable in other studies. Researchers tested the Qualtrics questionnaire links to ensure the survey proceeded without errors on both mobile devices and computers. Time to finish questionnaires was also tested to provide participants with accurate estimates of the time to complete the surveys.

6a-ii) Describe whether and how "use" (including intensity of use/dosage) was defined/measured/monitored

Describe whether and how "use" (including intensity of use/dosage) was defined/measured/monitored (logins, logfile analysis, etc.). Use/adoption metrics are important process outcomes that should be reported in any ehealth trial.

	1	2	3	4	5	
subitem not at all important	○	○	○	○	◉	essential

Clear selection

Does your paper address subitem 6a-ii?

Copy and paste relevant sections from manuscript text

"First, the difference in app engagement between conditions was examined. We compared usage of the 'Walk Now' feature between conditions using independent samples t-tests. Usage of the schedule and social functions was tallied for the intervention condition."

6a-iii) Describe whether, how, and when qualitative feedback from participants was obtained

Describe whether, how, and when qualitative feedback from participants was obtained (e.g., through emails, feedback forms, interviews, focus groups).

	1	2	3	4	5	
subitem not at all important	○	○	○	○	◉	essential

Clear selection

Does your paper address subitem 6a-iii?

Copy and paste relevant sections from manuscript text

"After the second week, researchers called participants to ask some open-ended feedback questions and ensure there were no problems with the app. If any problems arose during the course of the intervention, participants had access to a 'help' section within the app that included a phone number and email to contact researchers. This information was also included in the paper intervention materials that were mailed to them and attached to all email communications."

"At the completion of the one-month study, participants in both groups were again administered the shortened BTACT over the phone as well as some open-ended feedback questions, and then were asked to complete the post-study questionnaires online via Qualtrics."

6b) Any changes to trial outcomes after the trial commenced, with reasons

Does your paper address CONSORT subitem 6b? *

Copy and paste relevant sections from the manuscript (include quotes in quotation marks "like this" to indicate direct quotes from your manuscript), or elaborate on this item by providing additional information not in the ms, or briefly explain why the item is not applicable/relevant for your study

No changes were made to outcomes after trial commenced.

7a) How sample size was determined

NPT: When applicable, details of whether and how the clustering by care provides or centers was addressed

7a-i) Describe whether and how expected attrition was taken into account when calculating the sample size

Describe whether and how expected attrition was taken into account when calculating the sample size.

	1	2	3	4	5	
subitem not at all important	○	○	○	○	◉	essential

Clear selection

Does your paper address subitem 7a-i?

Copy and paste relevant sections from manuscript title (include quotes in quotation marks "like this" to indicate direct quotes from your manuscript), or elaborate on this item by providing additional information not in the ms, or briefly explain why the item is not applicable/relevant for your study

"An a priori power analysis for the primary outcome variable, number of steps, was conducted using G*Power version 3.1[37], which indicated that 31 participants per condition were required with an estimated effect size of d = .10, with 95% power at p = .05."

7b) When applicable, explanation of any interim analyses and stopping guidelines

Does your paper address CONSORT subitem 7b? *

Copy and paste relevant sections from the manuscript (include quotes in quotation marks "like this" to indicate direct quotes from your manuscript), or elaborate on this item by providing additional information not in the ms, or briefly explain why the item is not applicable/relevant for your study

Not applicable

8a) Method used to generate the random allocation sequence

NPT: When applicable, how care providers were allocated to each trial group

Does your paper address CONSORT subitem 8a? *

Copy and paste relevant sections from the manuscript (include quotes in quotation marks "like this" to indicate direct quotes from your manuscript), or elaborate on this item by providing additional information not in the ms, or briefly explain why the item is not applicable/relevant for your study

Not applicable

8b) Type of randomisation; details of any restriction (such as blocking and block size)

Does your paper address CONSORT subitem 8b? *

Copy and paste relevant sections from the manuscript (include quotes in quotation marks "like this" to indicate direct quotes from your manuscript), or elaborate on this item by providing additional information not in the ms, or briefly explain why the item is not applicable/relevant for your study

Added to procedures.

"A research assistant used Microsoft Excel for block randomization procedures. Blocks of ten consecutive ID numbers were randomly assigned to one of the two conditions. The app developer received the lists of ID numbers and associated conditions, so when an ID number was provided during the app download, the correct version of the app would install on the participant's phone. Upon meeting inclusion criteria and consenting to participate in the study, each participant was assigned an ID number which was paired to the condition generated from the block randomization."

9) Mechanism used to implement the random allocation sequence (such as sequentially numbered containers), describing any steps taken to conceal the sequence until interventions were assigned

Does your paper address CONSORT subitem 9? *

Copy and paste relevant sections from the manuscript (include quotes in quotation marks "like this" to indicate direct quotes from your manuscript), or elaborate on this item by providing additional information not in the ms, or briefly explain why the item is not applicable/relevant for your study

Added to procedures.

"A research assistant used Microsoft Excel for block randomization procedures. Blocks of ten consecutive ID numbers were randomly assigned to one of the two conditions. The app developer received the lists of ID numbers and associated conditions, so when an ID number was provided during the app download, the correct version of the app would install on the participant's phone. Upon meeting inclusion criteria and consenting to participate in the study, each participant was assigned an ID number which was paired to the condition generated from the block randomization."

10) Who generated the random allocation sequence, who enrolled participants, and who assigned participants to interventions

Does your paper address CONSORT subitem 10? *

Copy and paste relevant sections from the manuscript (include quotes in quotation marks "like this" to indicate direct quotes from your manuscript), or elaborate on this item by providing additional information not in the ms, or briefly explain why the item is not applicable/relevant for your study

Added to procedures.

"A research assistant used Microsoft Excel for block randomization procedures. Blocks of ten consecutive ID numbers were randomly assigned to one of the two conditions. The app developer received the lists of ID numbers and associated conditions, so when an ID number was provided during the app download, the correct version of the app would install on the participant's phone. Upon meeting inclusion criteria and consenting to participate in the study, each participant was assigned an ID number which was paired to the condition generated from the block randomization."

11a) If done, who was blinded after assignment to interventions (for example, participants, care providers, those assessing outcomes) and how

NPT: Whether or not administering co-interventions were blinded to group assignment

11a-i) Specify who was blinded, and who wasn't

Specify who was blinded, and who wasn't. Usually, in web-based trials it is not possible to blind the participants [1, 3] (this should be clearly acknowledged), but it may be possible to blind outcome assessors, those doing data analysis or those administering co-interventions (if any).

	1	2	3	4	5	
subitem not at all important	○	○	○	○	◉	essential

Clear selection

Does your paper address subitem 11a-i? *

Copy and paste relevant sections from the manuscript (include quotes in quotation marks "like this" to indicate direct quotes from your manuscript), or elaborate on this item by providing additional information not in the ms, or briefly explain why the item is not applicable/relevant for your study

Added to procedures.

"Participants were blinded to which condition they were assigned to receive. Although researchers were aware of the condition assignment for the purposes of helping with app downloads and troubleshooting issues, all measures with the exception of the cognitive assessments were done online without researcher involvement."

11a-ii) Discuss e.g., whether participants knew which intervention was the “intervention of interest” and which one was the “comparator”

Informed consent procedures (4a-ii) can create biases and certain expectations - discuss e.g., whether participants knew which intervention was the “intervention of interest” and which one was the “comparator”.

	1	2	3	4	5	
subitem not at all important	○	○	○	○	◉	essential

Clear selection

Does your paper address subitem 11a-ii?

Copy and paste relevant sections from the manuscript (include quotes in quotation marks "like this" to indicate direct quotes from your manuscript), or elaborate on this item by providing additional information not in the ms, or briefly explain why the item is not applicable/relevant for your study

Added to procedures.

"Participants were blinded to which condition they were assigned to receive. Although researchers were aware of the condition assignment for the purposes of helping with app downloads and troubleshooting issues, all measures with the exception of the cognitive assessments were done online without researcher involvement."

"After the posttest, participants in the control condition were given the opportunity to download the full version of the app, and all participants were encouraged to keep and use the app for their own personal use."

11b) If relevant, description of the similarity of interventions

(this item is usually not relevant for ehealth trials as it refers to similarity of a placebo or sham intervention to a active medication/intervention)

Does your paper address CONSORT subitem 11b? *

Copy and paste relevant sections from the manuscript (include quotes in quotation marks "like this" to indicate direct quotes from your manuscript), or elaborate on this item by providing additional information not in the ms, or briefly explain why the item is not applicable/relevant for your study

"The StepMATE app (Figure 1) was designed to help participants determine when, where, and with whom they would add physical activity into their day. Daily step goals were set by participants in both the intervention and control groups, and all participants had the ability to change their step goal at any point throughout the study. For the ‘when’ component, those in the intervention condition had a scheduling feature in the app. Participants could schedule a block of time to go for a walk, and had the option to create a reminder, set recurring events, and estimate the number of steps they would get in that walk. Once an event was created, it appeared in both the StepMATE app and in the iPhone’s built-in calendar.
For the ‘where’ component, those in the intervention group were able to create, name, and save walking routes in the app. When participants in the intervention group hit ‘Walk Now,’ StepMATE began keeping track of their geographical location, distance, number of steps, and total time of the walk. This information was then saved after the walk was finished, so the time it takes to walk a route could be compared if it is walked again. When a user created multiple routes, they could be filtered by number of steps or duration, so one could easily find a walk that fits the amount of time they have, or the number of steps needed to achieve their daily walking goal. Those in the control group still had a ‘walk now’ button, however, when control participants hit walk now, the app would simply track the number of steps taken in that walk. These participants were not able to name or save their walks, nor could they view their walks on a map.
For the ‘with whom’ (social feature), those in the intervention condition had the option to text one of their iPhone contacts through the app and invite them for a walk. Those in the control condition did not have access to this feature. Supplementary materials include screenshots of the app, video tutorials of the app features and differences between versions, and descriptions of other various app functions."

12a) Statistical methods used to compare groups for primary and secondary outcomes

NPT: When applicable, details of whether and how the clustering by care providers or centers was addressed

Does your paper address CONSORT subitem 12a? *

Copy and paste relevant sections from the manuscript (include quotes in quotation marks "like this" to indicate direct quotes from your manuscript), or elaborate on this item by providing additional information not in the ms, or briefly explain why the item is not applicable/relevant for your study

"Data analyses were conducted using RStudio Version 1.2.1335[33]. First, the difference in app engagement between conditions was examined. We compared usage of the 'Walk Now' feature between conditions using independent samples t-tests. Usage of the schedule and social functions was tallied for the intervention condition.
We tested the remainder of our hypotheses with multilevel mixed effects modeling with lme4 package[34], controlling for age, sex, education, and health. Using the following model, we tested whether weekly average steps increased from the baseline week to the four intervention weeks. Sensitivity analyses tested whether this effect differed if the baseline week was excluded. Interactions were specified to determine whether change in weekly step averages differed between conditions.

Level 1: Step Averageij = β0j + β1j (Week) + rij
Level 2: β0j = γ00 + γ01 (Agej) + γ02 (Sexj) + γ03 (Conditionj) + γ04 (Educationj) + γ05 (Healthj) + u0 j
β1j = γ10 + γ11(Conditionj)

Next, we used the model below to examine changes in the other outcome measures between pre- and post-test, including social engagement, exercise control and self-efficacy, memory, and sleep. Again, interactions were examined to determine whether change in outcomes differed between conditions.

Level 1: Outcome measureij = β0j + β1j (Time) + rij
Level 2: β0j = γ00 + γ01 (Agej) + γ02 (Sexj) + γ03 (Conditionj) + γ04 (Educationj) + γ05 (Healthj) + u0j
β1j = γ10 + γ11(Conditionj)

Finally, within-person relationships between daily steps, mood, and energy levels were tested. The models below tested whether daily steps were associated with same-day mood and energy. Lagged analyses were used to determine whether steps predicted next day mood/energy, controlling for previous day mood/energy. To parse out between- and within-person effects, models included both weekly average steps and daily deviation from average steps as predictors. Exploratory analyses examined whether sex or age moderated these effects. In instances when significant interactions with sex were found, separate models were run with males and females to probe the interaction. When significant age interactions were found, separate models were run by using a median split of age in our sample (62 years old).

Level 1: Daily Mood/Energyij = β0j + β1j (Daily steps) + rij
Level 2: β0j = γ00 + γ01 (Agej) + γ02 (Sexj) + γ03 (Conditionj) + γ04 (Educationj) + γ05 (Healthj) + γ06 (Average Stepsj) + u0j
β1j = γ10 + u1j"

12a-i) Imputation techniques to deal with attrition / missing values

Imputation techniques to deal with attrition / missing values: Not all participants will use the intervention/comparator as intended and attrition is typically high in ehealth trials. Specify how participants who did not use the application or dropped out from the trial were treated in the statistical analysis (a complete case analysis is strongly discouraged, and simple imputation techniques such as LOCF may also be problematic [4]).

	1	2	3	4	5	
subitem not at all important	○	○	○	◉	○	essential

Clear selection

Does your paper address subitem 12a-i? *

Copy and paste relevant sections from the manuscript (include quotes in quotation marks "like this" to indicate direct quotes from your manuscript), or elaborate on this item by providing additional information not in the ms, or briefly explain why the item is not applicable/relevant for your study

"While data indicate that older adults typically average between 2,000 and 9,000 steps per day [31], there are likely times participants walked without carrying their iPhone. For days when the iPhone recorded less than 500 steps, that day of steps was coded as missing. Weekly step averages were calculated for weeks with four or more days with 500 or more daily steps. Out of the 81 participants included in this intent-to-treat analysis, 11 participants had missing or incomplete baseline data."

12b) Methods for additional analyses, such as subgroup analyses and adjusted analyses

Does your paper address CONSORT subitem 12b? *

Copy and paste relevant sections from the manuscript (include quotes in quotation marks "like this" to indicate direct quotes from your manuscript), or elaborate on this item by providing additional information not in the ms, or briefly explain why the item is not applicable/relevant for your study

"Exploratory analyses examined whether sex or age moderated these effects. In instances when significant interactions with sex were found, separate models were run with males and females to probe the interaction. When significant age interactions were found, separate models were run by using a median split of age in our sample (62 years old)."

X26) REB/IRB Approval and Ethical Considerations [recommended as subheading under "Methods"] (not a CONSORT item)

X26-i) Comment on ethics committee approval

	1	2	3	4	5	
subitem not at all important	○	○	○	○	◉	essential

Clear selection

Does your paper address subitem X26-i?

Copy and paste relevant sections from the manuscript (include quotes in quotation marks "like this" to indicate direct quotes from your manuscript), or elaborate on this item by providing additional information not in the ms, or briefly explain why the item is not applicable/relevant for your study

"All procedures were approved by the University IRB."

x26-ii) Outline informed consent procedures

Outline informed consent procedures e.g., if consent was obtained offline or online (how? Checkbox, etc.?), and what information was provided (see 4a-ii). See [6] for some items to be included in informed consent documents.

	1	2	3	4	5	
subitem not at all important	○	○	○	○	◉	essential

Clear selection

Does your paper address subitem X26-ii?

Copy and paste relevant sections from the manuscript (include quotes in quotation marks "like this" to indicate direct quotes from your manuscript), or elaborate on this item by providing additional information not in the ms, or briefly explain why the item is not applicable/relevant for your study

Informed consent documents are included on clinicaltrials.gov.

X26-iii) Safety and security procedures

Safety and security procedures, incl. privacy considerations, and any steps taken to reduce the likelihood or detection of harm (e.g., education and training, availability of a hotline)

	1	2	3	4	5	
subitem not at all important	○	○	○	◉	○	essential

Clear selection

Does your paper address subitem X26-iii?

Copy and paste relevant sections from the manuscript (include quotes in quotation marks "like this" to indicate direct quotes from your manuscript), or elaborate on this item by providing additional information not in the ms, or briefly explain why the item is not applicable/relevant for your study

"If any problems arose during the course of the intervention, participants had access to a 'help' section within the app that included a phone number and email to contact researchers. This information was also included in the paper intervention materials that were mailed to them and attached to all email communications. "

RESULTS

13a) For each group, the numbers of participants who were randomly assigned, received intended treatment, and were analysed for the primary outcome

NPT: The number of care providers or centers performing the intervention in each group and the number of patients treated by each care provider in each center

Does your paper address CONSORT subitem 13a? *

Copy and paste relevant sections from the manuscript (include quotes in quotation marks "like this" to indicate direct quotes from your manuscript), or elaborate on this item by providing additional information not in the ms, or briefly explain why the item is not applicable/relevant for your study

CONSORT table (Figure 1)

13b) For each group, losses and exclusions after randomisation, together with reasons

Does your paper address CONSORT subitem 13b? (NOTE: Preferably, this is shown in a CONSORT flow diagram) *

Copy and paste relevant sections from the manuscript (include quotes in quotation marks "like this" to indicate direct quotes from your manuscript), or elaborate on this item by providing additional information not in the ms, or briefly explain why the item is not applicable/relevant for your study

CONSORT table (Figure 1)

13b-i) Attrition diagram

Strongly recommended: An attrition diagram (e.g., proportion of participants still logging in or using the intervention/comparator in each group plotted over time, similar to a survival curve) or other figures or tables demonstrating usage/dose/engagement.

	1	2	3	4	5	
subitem not at all important	○	◉	○	○	○	essential

Clear selection

Does your paper address subitem 13b-i?

Copy and paste relevant sections from the manuscript or cite the figure number if applicable (include quotes in quotation marks "like this" to indicate direct quotes from your manuscript), or elaborate on this item by providing additional information not in the ms, or briefly explain why the item is not applicable/relevant for your study

N/A.

We do not have login info for participants.

14a) Dates defining the periods of recruitment and follow-up

Does your paper address CONSORT subitem 14a? *

Copy and paste relevant sections from the manuscript (include quotes in quotation marks "like this" to indicate direct quotes from your manuscript), or elaborate on this item by providing additional information not in the ms, or briefly explain why the item is not applicable/relevant for your study

Manuscript updated.

"Participants were recruited online on a rolling basis between January 2018 and March 2019 using Facebook, Craigslist, and FindParticipants. Participants were also recruited locally throughout eastern and central Massachusetts, via flyers at senior centers, libraries, cafes, and community events. Because the study required no in-person meeting, participants were recruited from locations across the continental United States."

14a-i) Indicate if critical “secular events” fell into the study period

Indicate if critical “secular events” fell into the study period, e.g., significant changes in Internet resources available or “changes in computer hardware or Internet delivery resources”

	1	2	3	4	5	
subitem not at all important	◉	○	○	○	○	essential

Clear selection

Does your paper address subitem 14a-i?

Copy and paste relevant sections from the manuscript (include quotes in quotation marks "like this" to indicate direct quotes from your manuscript), or elaborate on this item by providing additional information not in the ms, or briefly explain why the item is not applicable/relevant for your study

Not applicable

14b) Why the trial ended or was stopped (early)

Does your paper address CONSORT subitem 14b? *

Copy and paste relevant sections from the manuscript (include quotes in quotation marks "like this" to indicate direct quotes from your manuscript), or elaborate on this item by providing additional information not in the ms, or briefly explain why the item is not applicable/relevant for your study

Not applicable

15) A table showing baseline demographic and clinical characteristics for each group

NPT: When applicable, a description of care providers (case volume, qualification, expertise, etc.) and centers (volume) in each group

Does your paper address CONSORT subitem 15? *

Copy and paste relevant sections from the manuscript (include quotes in quotation marks "like this" to indicate direct quotes from your manuscript), or elaborate on this item by providing additional information not in the ms, or briefly explain why the item is not applicable/relevant for your study

Included in manuscript.

Tables 1 and 2.

15-i) Report demographics associated with digital divide issues

In ehealth trials it is particularly important to report demographics associated with digital divide issues, such as age, education, gender, social-economic status, computer/Internet/ehealth literacy of the participants, if known.

	1	2	3	4	5	
subitem not at all important	○	○	○	◉	○	essential

Clear selection

Does your paper address subitem 15-i? *

Copy and paste relevant sections from the manuscript (include quotes in quotation marks "like this" to indicate direct quotes from your manuscript), or elaborate on this item by providing additional information not in the ms, or briefly explain why the item is not applicable/relevant for your study

Table 1.

"The current analyses included adults ages 50 years old and older (Mage = 61.87, SD = 7.82). 61 of 87 participants were females (70%), and 75 out of 87 participants were white (86%). Out of the sample of 87 participants, 2 participants being Asian, 9 reported being Black or African American, and 1 did not wish to report race. Participants were well educated, with an average of 16.45 years of education (SD = 2.56). Health was 69.25 on average (SD =17.40; as reported on the SF-36 general health subscale, with a 0-100 range). In our sample, 31% of participants reported working full time, 20% reported working part-time, 39% were retired, 7% reported they were self-employed, and 3% reported being a homemaker."

16) For each group, number of participants (denominator) included in each analysis and whether the analysis was by original assigned groups

16-i) Report multiple “denominators” and provide definitions

Report multiple “denominators” and provide definitions: Report N’s (and effect sizes) “across a range of study participation [and use] thresholds” [1], e.g., N exposed, N consented, N used more than x times, N used more than y weeks, N participants “used” the intervention/comparator at specific pre-defined time points of interest (in absolute and relative numbers per group). Always clearly define “use” of the intervention.

	1	2	3	4	5	
subitem not at all important	○	○	○	◉	○	essential

Clear selection

Does your paper address subitem 16-i? *

Copy and paste relevant sections from the manuscript (include quotes in quotation marks "like this" to indicate direct quotes from your manuscript), or elaborate on this item by providing additional information not in the ms, or briefly explain why the item is not applicable/relevant for your study

Tables 1 and 2

"Participants in both groups were able to use the ‘Walk Now’ feature; however, only the treatment group was able to see their walking routes on a map, name, and save them. In the control condition, 28 participants used the ‘Walk Now’ feature, with an average of 2,363 (SD = 1,616) steps per walk. In the treatment condition, 24 participants used the ‘Walk Now’ feature, with an average of 1,939 (SD = 791) steps per walk. An independent samples t-test showed that the group difference in average steps per walk was not significant t(50) = 1.17, p = .247. Based on the usage of the walk now feature, those in the control group took an average of 9 (SD = 11) walks, while those in the intervention group took an average of 11 (SD = 24) walks over the course of the one-month study. An independent samples t-test showed that there was no significant group difference in the average number of walks taken t(79) = -0.56, p = .576.

Of the participants in the treatment group, only 5 used the schedule feature at least once; one participant used the schedule feature three times, while the other 4 used it once. Only 4 of the treatment group participants used the social feature to text friends through the app; each of these participants used it one time during the one-month intervention."

16-ii) Primary analysis should be intent-to-treat

Primary analysis should be intent-to-treat, secondary analyses could include comparing only “users”, with the appropriate caveats that this is no longer a randomized sample (see 18-i).

	1	2	3	4	5	
subitem not at all important	○	○	○	○	◉	essential

Clear selection

Does your paper address subitem 16-ii?

Copy and paste relevant sections from the manuscript (include quotes in quotation marks "like this" to indicate direct quotes from your manuscript), or elaborate on this item by providing additional information not in the ms, or briefly explain why the item is not applicable/relevant for your study

Analyses throughout the manuscript have been updated to reflect an intent to treat all participants for which we have baseline data.

"Analyses were conducted on an intent-to-treat basis; for the questionnaire data, we analyzed all participants who completed pre-test measures (n=87). For the daily step analyses, all participants with sufficient step data were included in the analyses (n=80). Sample sizes are included in all results tables for clarity."

17a) For each primary and secondary outcome, results for each group, and the estimated effect size and its precision (such as 95% confidence interval)

Does your paper address CONSORT subitem 17a? *

Copy and paste relevant sections from the manuscript (include quotes in quotation marks "like this" to indicate direct quotes from your manuscript), or elaborate on this item by providing additional information not in the ms, or briefly explain why the item is not applicable/relevant for your study

Standard errors are included for all estimates. Model fit statistics, including AIC, BIC, and -2LL are included for analyses.

17a-i) Presentation of process outcomes such as metrics of use and intensity of use

In addition to primary/secondary (clinical) outcomes, the presentation of process outcomes such as metrics of use and intensity of use (dose, exposure) and their operational definitions is critical. This does not only refer to metrics of attrition (13-b) (often a binary variable), but also to more continuous exposure metrics such as “average session length”. These must be accompanied by a technical description how a metric like a “session” is defined (e.g., timeout after idle time) [1] (report under item 6a).

	1	2	3	4	5	
subitem not at all important	○	○	○	○	◉	essential

Clear selection

Does your paper address subitem 17a-i?

Copy and paste relevant sections from the manuscript (include quotes in quotation marks "like this" to indicate direct quotes from your manuscript), or elaborate on this item by providing additional information not in the ms, or briefly explain why the item is not applicable/relevant for your study

"Participants in both groups were able to use the 'Walk Now' feature; however, only the treatment group was able to see their walking routes on a map, name, and save them. In the control condition, 28 participants used the 'Walk Now' feature, with an average of 2,363 (SD = 1,616) steps per walk. In the treatment condition, 24 participants used the 'Walk Now' feature, with an average of 1,939 (SD = 791) steps per walk. An independent samples t-test showed that the group difference in average steps per walk was not significant t(50) = 1.17, p = .247. Based on the usage of the walk now feature, those in the control group took an average of 9 (SD = 11) walks, while those in the intervention group took an average of 11 (SD = 24) walks over the course of the one-month study. An independent samples t-test showed that there was no significant group difference in the average number of walks taken t(79) = -0.56, p = .576.

Of the participants in the treatment group, only 5 used the schedule feature at least once; one participant used the schedule feature three times, while the other 4 used it once. Only 4 of the treatment group participants used the social feature to text friends through the app; each of these participants used it one time during the one-month intervention.

In terms of correlations between covariates and app usage, those who were older used the walk now feature more often (r = .26, p = .02). Age was not significantly correlated with usage of the schedule or social features. Neither sex nor education were significantly correlated with use of the walk now, schedule, or social features."

17b) For binary outcomes, presentation of both absolute and relative effect sizes is recommended

Does your paper address CONSORT subitem 17b? *

Copy and paste relevant sections from the manuscript (include quotes in quotation marks "like this" to indicate direct quotes from your manuscript), or elaborate on this item by providing additional information not in the ms, or briefly explain why the item is not applicable/relevant for your study

Not applicable

18) Results of any other analyses performed, including subgroup analyses and adjusted analyses, distinguishing pre-specified from exploratory

Does your paper address CONSORT subitem 18? *

Copy and paste relevant sections from the manuscript (include quotes in quotation marks "like this" to indicate direct quotes from your manuscript), or elaborate on this item by providing additional information not in the ms, or briefly explain why the item is not applicable/relevant for your study

"Exploratory analyses examined whether sex or age moderated these effects. In instances when significant interactions with sex were found, separate models were run with males and females to probe the interaction. When significant age interactions were found, separate models were run by using a median split of age in our sample (62 years old)."

18-i) Subgroup analysis of comparing only users

A subgroup analysis of comparing only users is not uncommon in ehealth trials, but if done, it must be stressed that this is a self-selected sample and no longer an unbiased sample from a randomized trial (see 16-iii).

	1	2	3	4	5	
subitem not at all important	(•)	○	○	○	○	essential

Clear selection

Does your paper address subitem 18-i?

Copy and paste relevant sections from the manuscript (include quotes in quotation marks "like this" to indicate direct quotes from your manuscript), or elaborate on this item by providing additional information not in the ms, or briefly explain why the item is not applicable/relevant for your study

Not applicable

19) All important harms or unintended effects in each group

(for specific guidance see CONSORT for harms)

Does your paper address CONSORT subitem 19? *

Copy and paste relevant sections from the manuscript (include quotes in quotation marks "like this" to indicate direct quotes from your manuscript), or elaborate on this item by providing additional information not in the ms, or briefly explain why the item is not applicable/relevant for your study

Not applicable

19-i) Include privacy breaches, technical problems

Include privacy breaches, technical problems. This does not only include physical “harm” to participants, but also incidents such as perceived or real privacy breaches [1], technical problems, and other unexpected/unintended incidents. “Unintended effects” also includes unintended positive effects [2].

	1	2	3	4	5	
subitem not at all important	○	○	○	○	◉	essential

Clear selection

Does your paper address subitem 19-i?

Copy and paste relevant sections from the manuscript (include quotes in quotation marks "like this" to indicate direct quotes from your manuscript), or elaborate on this item by providing additional information not in the ms, or briefly explain why the item is not applicable/relevant for your study

"Following an iPhone software update, the StepMATE app crashed and did not work properly for approximately two weeks. There were 18 participants affected, and daily step data was lost for these participants."

19-ii) Include qualitative feedback from participants or observations from staff/researchers

Include qualitative feedback from participants or observations from staff/researchers, if available, on strengths and shortcomings of the application, especially if they point to unintended/unexpected effects or uses. This includes (if available) reasons for why people did or did not use the application as intended by the developers.

	1	2	3	4	5	
subitem not at all important	○	○	○	○	◉	essential

Clear selection

Does your paper address subitem 19-ii?

Copy and paste relevant sections from the manuscript (include quotes in quotation marks "like this" to indicate direct quotes from your manuscript), or elaborate on this item by providing additional information not in the ms, or briefly explain why the item is not applicable/relevant for your study

Added to discussion It is also possible that self-monitoring and goal setting are enough to encourage increases in daily walking.

"Qualitative feedback from participants echo this notion; “I love when I look at my steps for the day and see that I get close or exceed my daily step goals! I am a person who needs to exercise more, and this app reminds me to keep it moving!” “Kept track of steps, spot on. Mood questions made me aware of steps, I am checking steps more often and more aware of reaching my goal.” These results are consistent with prior findings that goal setting is among the most successful behavior change techniques for increasing physical activity[5]."

DISCUSSION

22) Interpretation consistent with results, balancing benefits and harms, and considering other relevant evidence

NPT: In addition, take into account the choice of the comparator, lack of or partial blinding, and unequal expertise of care providers or centers in each group

22-i) Restate study questions and summarize the answers suggested by the data, starting with primary outcomes and process outcomes (use)

Restate study questions and summarize the answers suggested by the data, starting with primary outcomes and process outcomes (use).

	1	2	3	4	5	
subitem not at all important	○	○	○	○	◉	essential

Clear selection

Does your paper address subitem 22-i? *

Copy and paste relevant sections from the manuscript (include quotes in quotation marks "like this" to indicate direct quotes from your manuscript), or elaborate on this item by providing additional information not in the ms, or briefly explain why the item is not applicable/relevant for your study

"The current study was a pilot randomized control trial (RCT), which examined the effectiveness of StepMATE, a newly developed iPhone app aimed at increasing daily steps in a sample of middle aged and older adults. The app included behavioral supports, including goal setting and feedback, action planning, and social support to encourage changes in behavior. Average daily steps were significantly higher in the 4-week intervention compared to the baseline week, and these increases were maintained over the course of the study. The increase in steps, however, did not differ between the two groups.
Contrary to hypotheses, there were no differences in physical activity outcomes between the basic, pedometer-like control condition and the full behavioral strategies versions of the StepMATE app. This is likely because participants in both conditions used the features to a similar extent. Despite having different versions of the app, the treatment condition participants rarely used the additional features available to them. It is possible that extra supports may not be needed, or that the participants may have considered the extra features difficult or too time-consuming to use.
It is also possible that self-monitoring and goal setting are enough to encourage increases in daily walking, as other studies have shown[5], [6]."

"Although there was a significant increase in cognition from pre-to post-test, there were no significant changes for any of the other outcomes, including social engagement, exercise control, or exercise self-efficacy. While this is consistent with other findings that exercise is associated with improvements in cognition[38], [39], the increase in cognitive performance between pre- and post-test could be due to retest effects. The same version of the test was given on both occasions. We also tested whether change in steps between baseline and the end of the intervention were correlated with cognitive performance; no significant correlations emerged.
Also contrary to hypotheses, there were no significant improvements in sleep. Examining the Pittsburgh Sleep Quality Index (PSQI) global score at pre-test, participants in general were good sleepers, with an average sleep duration of just under 7 hours, and sleep latencies under 20 minutes, on average. It is possible that a ceiling effect could explain the lack of change in sleep over the four-week study. To determine whether sleep improved for those with poorer sleep at baseline, post-hoc analyses were conducted with a median split of PSQI global scores. There were no changes from pre-to post-test for either good sleepers (PSQI global of 4 or less), or those with scores higher than 4."

22-ii) Highlight unanswered new questions, suggest future research

Highlight unanswered new questions, suggest future research.

	1	2	3	4	5	
subitem not at all important	○	○	○	○	◉	essential

Clear selection

Does your paper address subitem 22-ii?

Copy and paste relevant sections from the manuscript (include quotes in quotation marks "like this" to indicate direct quotes from your manuscript), or elaborate on this item by providing additional information not in the ms, or briefly explain why the item is not applicable/relevant for your study

"It is also possible that because our pilot study only lasted four weeks, it was not long enough to elicit changes in our outcome measures. Future work should assess physical activity and subsequent changes in outcomes over longer time periods, with longer baseline and follow up periods. This work would allow for researchers to assess whether changes in physical activity are maintained even after the novelty of a behavior change intervention has worn off."

"Although we do have data on whether participants were working full time, part time, or retired, future work could address whether certain professions are more or less likely to engage in physical activity. This could aid in development of targeted interventions for groups who are most inactive."

"In the present study, participants also may not have set their step goals high enough to challenge themselves or encourage increases in walking. Future studies should continue to examine which behavioral supports are most successful in increasing physical activity in older adult populations and find best practices for incorporating these supports into successful physical activity interventions. "

"Future work could more closely examine sex and age differences in the relationship between walking, mood and energy. Such research could uncover which features of apps are the most successful and motivating to both men and women across adulthood and could lead to the development of large-scale technology-based interventions for increasing physical activity."

20) Trial limitations, addressing sources of potential bias, imprecision, and, if relevant, multiplicity of analyses

20-i) Typical limitations in ehealth trials

Typical limitations in ehealth trials: Participants in ehealth trials are rarely blinded. Ehealth trials often look at a multiplicity of outcomes, increasing risk for a Type I error. Discuss biases due to non-use of the intervention/usability issues, biases through informed consent procedures, unexpected events.

	1	2	3	4	5	
subitem not at all important	○	○	○	○	◉	essential

Clear selection

Does your paper address subitem 20-i? *

Copy and paste relevant sections from the manuscript (include quotes in quotation marks "like this" to indicate direct quotes from your manuscript), or elaborate on this item by providing additional information not in the ms, or briefly explain why the item is not applicable/relevant for your study

"The current study has some limitations that are worthy of consideration. The app was only available to users of iPhones with step tracking capabilities, so there may have been selection bias in only recruiting users who have a fairly new iPhone. The generalizability of the study was also limited by a relatively small sample, who were mostly white, well-educated older adults. According to the Pew research center, white individuals, those with higher education, and higher household income are more likely to be smartphone owners[43]. Of smartphone owners, iPhone owners in particular are more likely to be white, with higher education and income[44]. Although we do have data on whether participants were working full time, part time, or retired, future work could address whether certain professions are more or less likely to engage in physical activity. This could aid in development of targeted interventions for groups who are most inactive. Another limitation is that we did not assess whether participants were using fitness technology or apps prior to enrolling in our study. We specifically recruited individuals who believed they needed to increase their physical activity, so it's likely that even if participants used these devices in the past, they were not successful in changing long-term behaviors.

Because the study was conducted on a rolling basis over the course of a year in different locations, it is possible that seasonal or geographical factors may have played a role in the findings. The validity of the baseline week steps is also unclear. It is possible that participants' steps in the week prior to the intervention may not be representative of their typical daily walking. During the four-week intervention, participants were given a pouch for their phone and were specifically asked to carry their phone with them during the day. They were not explicitly instructed to do so before the intervention began. Some participants (n=11) did not have step-tracking enabled on their iPhone prior to the study, so they did not have any baseline data. These participants were still included in all analyses as they had step data during the intervention. Post-hoc sensitivity analyses revealed that results did not change if these participants were excluded. Future studies should aim to collect baseline data for longer periods of time, to obtain a more accurate estimate of normative physical activity levels prior to an intervention.

Measuring physical activity with a smartphone poses limitations. First, the accuracy of measurement may be a limitation. While some studies and meta analyses suggest that smartphones and iPhones in particular provide accurate and valid measures, especially in terms of differentiating walking from sedentary behaviors[45], [46], others suggest that iPhones may be prone to underestimating steps[47]. There could also be accuracy differences based on iPhone model. Participants may have forgotten to carry their phones with them at different points throughout the day. It is possible that participants could have given their phones to others to increase their step counts. Qualitative feedback from participants suggests that most in our sample kept their phones on their person for the majority of the day. Because participants kept their phones with them throughout the day, many of the steps may not have been taken with the intention of walking from exercise. The goal was to capture a full picture of daily activity in our study, as walking is an exercise modality that can easily be incorporated into one's regular routine throughout the day. Thus, we did not differentiate whether steps were taken for exercise or not.

The intervention was personalized by allowing participants to use the app at their

The intervention was personalized by allowing participants to use the app at their convenience and to set and change their walking goals as often as desired. This was designed to mirror what would happen if one independently downloaded a new walking app and used it on their own. It is possible that participants did not use the features of the app because they were not specifically asked to do so. In contrast, in another study that used similar behavioral features[13] participants were reminded daily to use the calendars and maps and to set goals. In the present study, participants also may not have set their step goals high enough to challenge themselves or encourage increases in walking. Future studies should continue to examine which behavioral supports are most successful in increasing physical activity in older adult populations and find best practices for incorporating these supports into successful physical activity interventions.
Finally, participants could have encountered some difficulties using the app and might have preferred a lower-tech intervention for increasing steps. Future work should compare how different age groups can be motivated to increase their activity, especially by making technology more user friendly and age appropriate. Technology has the potential to assess multiple outcomes (e.g., health data, ecological momentary assessments) in real time, such as through a smartphone, a device that most adults already carry around with them daily[48]."

21) Generalisability (external validity, applicability) of the trial findings

NPT: External validity of the trial findings according to the intervention, comparators, patients, and care providers or centers involved in the trial

21-i) Generalizability to other populations

Generalizability to other populations: In particular, discuss generalizability to a general Internet population, outside of a RCT setting, and general patient population, including applicability of the study results for other organizations

	1	2	3	4	5	
subitem not at all important	○	○	○	●	○	essential

Clear selection

Does your paper address subitem 21-i?

Copy and paste relevant sections from the manuscript (include quotes in quotation marks "like this" to indicate direct quotes from your manuscript), or elaborate on this item by providing additional information not in the ms, or briefly explain why the item is not applicable/relevant for your study

"The app was only available to users of iPhones with step tracking capabilities, so there may have been selection bias in only recruiting users who have a fairly new iPhone. The generalizability of the study was also limited by a relatively small sample, who were mostly white, well-educated older adults. According to the Pew research center, white individuals, those with higher education, and higher household income are more likely to be smartphone owners[43]. Of smartphone owners, iPhone owners in particular are more likely to be white, with higher education and income[44]."

21-ii) Discuss if there were elements in the RCT that would be different in a routine application setting

Discuss if there were elements in the RCT that would be different in a routine application setting (e.g., prompts/reminders, more human involvement, training sessions or other co-interventions) and what impact the omission of these elements could have on use, adoption, or outcomes if the intervention is applied outside of a RCT setting.

	1	2	3	4	5	
subitem not at all important	○	○	○	◉	○	essential

Clear selection

Does your paper address subitem 21-ii?

Copy and paste relevant sections from the manuscript (include quotes in quotation marks "like this" to indicate direct quotes from your manuscript), or elaborate on this item by providing additional information not in the ms, or briefly explain why the item is not applicable/relevant for your study

"The intervention was personalized by allowing participants to use the app at their convenience and to set and change their walking goals as often as desired. This was designed to mirror what would happen if one independently downloaded a new walking app and used it on their own. It is possible that participants did not use the features of the app because they were not specifically asked to do so."

OTHER INFORMATION

23) Registration number and name of trial registry

Does your paper address CONSORT subitem 23? *

Copy and paste relevant sections from the manuscript (include quotes in quotation marks "like this" to indicate direct quotes from your manuscript), or elaborate on this item by providing additional information not in the ms, or briefly explain why the item is not applicable/relevant for your study

Included in manuscript.

"ClinicalTrials.gov NCT03124537."

24) Where the full trial protocol can be accessed, if available

Does your paper address CONSORT subitem 24? *

Cite a Multimedia Appendix, other reference, or copy and paste relevant sections from the manuscript (include quotes in quotation marks "like this" to indicate direct quotes from your manuscript), or elaborate on this item by providing additional information not in the ms, or briefly explain why the item is not applicable/relevant for your study

The trial protocol is discussed in the manuscript and also at clinicaltrials.gov.

"A research assistant used Microsoft Excel for block randomization procedures. Blocks of ten consecutive ID numbers were randomly assigned to one of the two conditions. The app developer received the lists of ID numbers and associated conditions, so when an ID number was provided during the app download, the correct version of the app would install on the participant's phone. Upon meeting inclusion criteria and consenting to participate in the study, each participant was assigned an ID number which was paired to the condition generated from the block randomization. Then participants were administered a reduced version of the Brief Test of Adult Cognition by Telephone (BTACT) over the phone and completed the pre-study questionnaires online via Qualtrics, including self-assessed social engagement, exercise control, exercise self-efficacy, and sleep. Participants filled out their ID number at the beginning of the Qualtrics survey so their self-report data could easily be linked to their step data.
Researchers then scheduled a phone call to help participants download the app, setup their account including daily walking goals, and thoroughly explain the app features. Those in the control condition downloaded a version with only the daily step goals and the ability to track time, distance, and steps within a walk. The treatment condition had access to these and additional features, including schedules, maps, and social features. Participants were blinded to which condition they were assigned to receive. Although researchers were aware of the condition assignment for the purposes of helping with app downloads and troubleshooting issues, all measures with the exception of the cognitive assessments were done online without researcher involvement. Randomization was checked by comparing the covariates (age, sex, education, and general health) between conditions using independent samples t-tests. No significant differences were found between conditions for any of these variables.
Participants in both groups were asked to use the app for one month and do their best to answer the daily mood and energy questions. All participants were sent a pouch to wear around their waists and were encouraged to use it to carry their phone with them as much as possible until going to bed each night. After the first and third weeks, participants received an email letting them know how many weeks they had been in the study and how many remained. After the second week, researchers called participants to ask some open-ended feedback questions and ensure there were no problems with the app. If any problems arose during the course of the intervention, participants had access to a 'help' section within the app that included a phone number and email to contact researchers. This information was also included in the paper intervention materials that were mailed to them and attached to all email communications.
At the completion of the one-month study, participants in both groups were again administered the shortened BTACT over the phone as well as some open-ended feedback questions, and then were asked to complete the post-study questionnaires online via Qualtrics. After completing the questionnaires, participants were sent a $25 Amazon gift card via email. After the posttest, participants in the control condition were given the opportunity to download the full version of the app, and all participants were encouraged to keep and use the app for their own personal use."

25) Sources of funding and other support (such as supply of drugs), role of funders

Does your paper address CONSORT subitem 25? *

Copy and paste relevant sections from the manuscript (include quotes in quotation marks "like this" to indicate direct quotes from your manuscript), or elaborate on this item by providing additional information not in the ms, or briefly explain why the item is not applicable/relevant for your study

Included on title page.

"We acknowledge grant support from the National Institute on Aging Grant # P30 AG048785 for a Roybal Center for Translational Research on Aging. This study is registered at www.clinicaltrials.gov (No. NCT03124537)."

X27) Conflicts of Interest (not a CONSORT item)

X27-i) State the relation of the study team towards the system being evaluated

In addition to the usual declaration of interests (financial or otherwise), also state the relation of the study team towards the system being evaluated, i.e., state if the authors/evaluators are distinct from or identical with the developers/sponsors of the intervention.

	1	2	3	4	5	
subitem not at all important	○	○	○	◉	○	essential

Clear selection

Does your paper address subitem X27-i?

Copy and paste relevant sections from the manuscript (include quotes in quotation marks "like this" to indicate direct quotes from your manuscript), or elaborate on this item by providing additional information not in the ms, or briefly explain why the item is not applicable/relevant for your study

Title page modified.

"All authors declare this research was conducted in the absence of any financial conflicts of interest. "

About the CONSORT EHEALTH checklist

As a result of using this checklist, did you make changes in your manuscript? *

- (•) yes, major changes
- ( ) yes, minor changes
- ( ) no

What were the most important changes you made as a result of using this checklist?

Including intent to treat analyses in manuscript, clarifying procedures and the app, uploading codes to apply an 'open science' framework.

How much time did you spend on going through the checklist INCLUDING making changes in your manuscript *

About 25-30 hours.

As a result of using this checklist, do you think your manuscript has improved? *

- (x) yes
- ( ) no
- ( ) Other:

Would you like to become involved in the CONSORT EHEALTH group?

This would involve for example becoming involved in participating in a workshop and writing an "Explanation and Elaboration" document

- ( ) yes
- (x) no
- ( ) Other:

Clear selection

Any other comments or questions on CONSORT EHEALTH

Your answer

STOP - Save this form as PDF before you click submit

To generate a record that you filled in this form, we recommend to generate a PDF of this page (on a Mac, simply select "print" and then select "print as PDF") before you submit it.

When you submit your (revised) paper to JMIR, please upload the PDF as supplementary file.

Don't worry if some text in the textboxes is cut off, as we still have the complete information in our database. Thank you!

Final step: Click submit !

Click submit so we have your answers in our database!

Submit

Never submit passwords through Google Forms.

This content is neither created nor endorsed by Google. Report Abuse - Terms of Service - Privacy Policy

Google Forms
